# Proteotyping of biogas plant microbiomes separates biogas plants according to process temperature and reactor type

**DOI:** 10.1186/s13068-016-0572-4

**Published:** 2016-07-26

**Authors:** R. Heyer, D. Benndorf, F. Kohrs, J. De Vrieze, N. Boon, M. Hoffmann, E. Rapp, Andreas Schlüter, Alexander Sczyrba, U. Reichl

**Affiliations:** 1 Bioprocess Engineering, Max Planck Institute for Dynamics of Complex Technical Systems, Sandtorstraße 1, 39106 Magdeburg, Germany; 2 Bioprocess Engineering, Otto von Guericke University Magdeburg, Universitätsplatz 2, 39106 Magdeburg, Germany; 3 Laboratory of Microbial Technology and Ecology (LabMET), Ghent University, Coupure Links 653, 9000 Ghent, Belgium; 4 Center for Biotechnology, Genome Research of Industrial Microorganisms, Universität Bielefeld, Universitätsstraße 25, 33615 Bielefeld, Germany; 5 Center for Biotechnology, Computational Metagenomics, Universität Bielefeld, Universitätsstr. 25, 33615 Bielefeld, Germany

**Keywords:** Biogas, Biogas plant, Anaerobic digestion, Metaproteomics, Community function, Microbial resource management, MetaProteomeAnalyzer, Clustering, Principal component analysis, Network analysis machine learning

## Abstract

**Background:**

Methane yield and biogas productivity of biogas plants (BGPs) depend on microbial community structure and function, substrate supply, and general biogas process parameters. So far, however, relatively little is known about correlations between microbial community function and process parameters. To close this knowledge gap, microbial communities of 40 samples from 35 different industrial biogas plants were evaluated by a metaproteomics approach in this study.

**Results:**

Liquid chromatography coupled to tandem mass spectrometry (Orbitrap Elite™ Hybrid Ion Trap-Orbitrap Mass Spectrometer) of all 40 samples as triplicate enabled the identification of 3138 different metaproteins belonging to 162 biological processes and 75 different taxonomic orders. The respective database searches were performed against UniProtKB/Swiss-Prot and seven metagenome databases. Subsequent clustering and principal component analysis of these data allowed for the identification of four main clusters associated with mesophile and thermophile process conditions, the use of upflow anaerobic sludge blanket reactors and BGP feeding with sewage sludge. Observations confirm a previous phylogenetic study of the same BGP samples that was based on 16S rRNA gene sequencing by De Vrieze et al. (Water Res 75:312–323, 2015). In particular, we identified similar microbial key players of biogas processes, namely *Bacillales, Enterobacteriales, Bacteriodales, Clostridiales, Rhizobiales and Thermoanaerobacteriales* as well as *Methanobacteriales, Methanosarcinales* and *Methanococcales.* For the elucidation of the main biomass degradation pathways, the most abundant 1 % of metaproteins was assigned to the KEGG map 1200 representing the central carbon metabolism. Additionally, the effect of the process parameters (i) temperature, (ii) organic loading rate (OLR), (iii) total ammonia nitrogen (TAN), and (iv) sludge retention time (SRT) on these pathways was investigated. For example, high TAN correlated with hydrogenotrophic methanogens and bacterial one-carbon metabolism, indicating syntrophic acetate oxidation.

**Conclusions:**

This is the first large-scale metaproteome study of BGPs. Proteotyping of BGPs reveals general correlations between the microbial community structure and its function with process parameters. The monitoring of changes on the level of microbial key functions or even of the microbial community represents a well-directed tool for the identification of process problems and disturbances.

**Graphical abstract:**
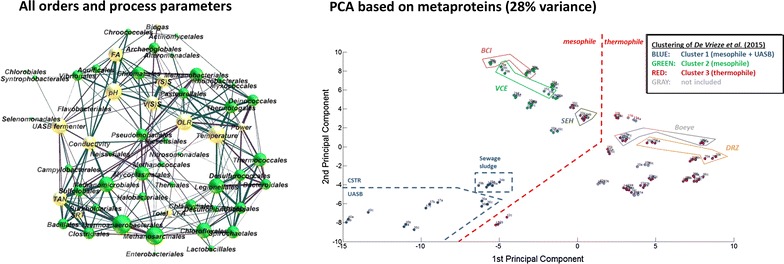
Correlation between the different orders and process parameter, as well as principle component analysis of all investigated biogas plants based on the identified metaproteins.

**Electronic supplementary material:**

The online version of this article (doi:10.1186/s13068-016-0572-4) contains supplementary material, which is available to authorized users.

## Background

The conversion of biological waste to methane in biogas plants (BGPs) is one of the main renewable energy sources in Germany. Currently, more than 8000 BGPs cover about 3 % of the total annual energy demand [2]. Each BGP has its individual operation conditions and specific process parameters due to differences in plant design, regional substrate availability, and operator’s considerations. As a consequence, cultivation conditions differ significantly between BPGs. Thus, each BGP has its own signature regarding the composition and function of the microbial community which catalyzes the conversion of complex substrates to methane and carbon dioxide.

Hydrolysis, fermentation, acetogenesis, and methanogenesis—the basic steps of anaerobic digestion—are catalyzed by different members of a microbial community interacting with each other. The interactions of the various strains in anaerobic digestion are characterized by dependencies on different trophic levels. For example, secondary fermenters are strictly dependent on hydrogenotrophic methanogens. This syntrophic interaction, characterized by interspecies hydrogen transfer [3], keeps the concentration of hydrogen sufficiently low to make secondary fermentation thermodynamically feasible. Nevertheless, in situ bioaugmentation by hydrogen producers leads to intensified biogas production indicating that hydrogen is a bottleneck in the overall process [4]. For robust biogas production with high yields, the rates of different reactions and, consequently, the occurrence and activity of the microbes in the community should be well balanced. Otherwise, undesired by-products, such as short chain organic acids, can accumulate causing unwanted acidification of the BGP. Variations in the composition of substrates and process conditions, for example temperature, pH value or ammonia concentration are challenging because the balance has to be readjusted immediately by metabolic adaptation of the actual community, and by long-term changes in the community composition. Accordingly, the composition of microbial communities of BGPs was reported to vary even after small changes in process conditions [5]. On the other hand, it may also remain at steady state over long periods of operation [6]. Thus, robustness against external factors is considered crucial for BGP operation. Several authors [5, 7, 8] correlated BGP robustness with high values of the ecological indice richness and low values of the ecological indice evenness. Hence, both highly abundant key players and less abundant species are required to achieve high performance and high process stability.

Correlations between process parameters and microbial communities cannot be inferred from analysis of a single BGP, due to individual operation conditions and specific process parameters of each BGP. Studies involving a large number of BGPs demonstrated, for example, that the composition of microbial communities was mainly correlated with the substrate, process temperature and ammonia content of the BGPs [1, 9, 10]. So far, however, most studies were conducted by sequencing of 16S rRNA genes covering exclusively community composition, but not metabolic function. Therefore, metagenomic [11], metatranscriptomic [12] or metaproteomic [13] approaches are more informative for the investigation of complex microbial communities in BGPs [14]. While metagenomics covers the genetic potential, metatranscriptomics and metaproteomics determine the actual gene expression and better represent the physiological state of the microbial communities. Interestingly, the comparison of metagenomics and metaproteomics results revealed differences in the community composition [15]. In particular, the proportion of methanogenic *Archaea* within microbial communities has been underestimated using metagenomics or 16S rRNA sequencing in earlier studies [5]. Recent studies showed higher proportions of methanogenic archaea [41, 42]. For 16S rRNA, sequencing bias could be caused by the use of polymerase chain reaction (PCR) [16, 17] whereas bias during sample extraction is a challenge for all omics approaches. Advantages and disadvantages of metaproteomics and other approaches for analysis of BGPs were extensively reviewed by Heyer et al. [13].

Up to date, the lack of corresponding metagenome data and high experimental efforts prevented a widespread application of metaproteomics for routine analysis of BGPs. However, the availability of recent high-resolution mass spectrometry (MS) increases tremendously the depth of metaproteome analyses and, therefore, renders extensive sample pre-fractionation unnecessary. Furthermore, the identification and annotation of proteins in metaproteomics can be improved by specifically adapted software solutions (e.g., MetaProteomeAnalyzer (MPA) [18]) and searching against specific metagenome databases (e.g., metagenomes of agricultural BGPs). In the following, this approach was used to investigate the functional differences of microbial communities within different biogas plant and their correlations to the process conditions for nearly the same 40 samples as used in a 16S rRNA gene-based study recently published by De Vrieze et al. [1].

In addition, the quality of metaproteomic data with respect to community composition and the classification of BGPs were evaluated against the 16S rRNA gene-based approach. Overall, the performed metaproteomics approach showed similar results concerning the taxonomic composition of the microbial communities as the study of De Vrieze et al. [1], and revealed several correlations between the process parameters and the function of the microbial community.

## Results and discussion

### Biogas plant process parameters

Forty samples from 35 different full-scale BGPs were investigated by metaproteomics concerning the taxonomic and functional composition of their microbial communities. Thirty-four samples were identical to samples previously analyzed by De Vrieze et al. [1] allowing for a comparison of the taxonomic results based on this metaproteomics approach to the published data based on 16S rRNA amplicon sequencing and real-time PCR. The samples covered different reactor types, namely continuously stirred tank reactors (CSTRs) and upflow anaerobic sludge blanket (UASB) reactor systems as well as a wide range of substrates (agricultural substrates, industrial waste, slaughter house waste, sewage sludge, municipal waste, mixed/unknown substrates) (Additional file 1: Table S1). Other differences were: (i) mesophile (33–35 °C) and thermophile (40–54 °C) process conditions, (ii) a range in the organic loading rate (OLR) from 1.5 kg_COD_/(m^3^d) to 11 kg_COD_/(m^3^d), and (iii) pH values from 7.1 to 8.6. Some process parameters were correlated, for example high temperatures and OLR with high biogas productivity $\left( {{\text{m}}_{\text{biogas}}^{3} /{\text{m}}_{\text{fermenter volume}}^{3} {\text{d}}} \right)$ (Additional file 1: Table S2).

### Protein identification

Proteins from all BGPs were successfully extracted and their quality was assessed by sodium dodecyl sulfate polyacrylamide gel electrophoresis. Subsequent LC–MS/MS based protein identification using an Orbitrap Elite™ Hybrid Ion Trap-Orbitrap MS and database search against UniProtKB/Swiss-Prot including several metagenomes [11, 15, 19, 20] using a false discovery rate of 1 % revealed up to 4000 identified spectra and 8000 proteins for each BGP. Subsequent grouping of redundant protein identifications to metaproteins by their affiliation to UniRef 50 clusters [21] reduced the number of different metaproteins to less than 900 for a single BGP and to 3138 for all BGPs. The quantity of metaproteins and the associated number of taxonomic orders and biological processes was further downsized by the application of a threshold to include only metaproteins represented by at least 1 % of the spectra in at least one BGP. In a last step, a correlation matrix of all metaproteins, taxonomic orders, biological processes, as well as process parameters was generated for further analyses. For a detailed documentation of all these steps, please refer to the Note 1 (Additional files 2, 3, 4, 5, 6, 7).

### Grouping of biogas plants based on cluster analysis and principal component analysis

**Fig. 1:**
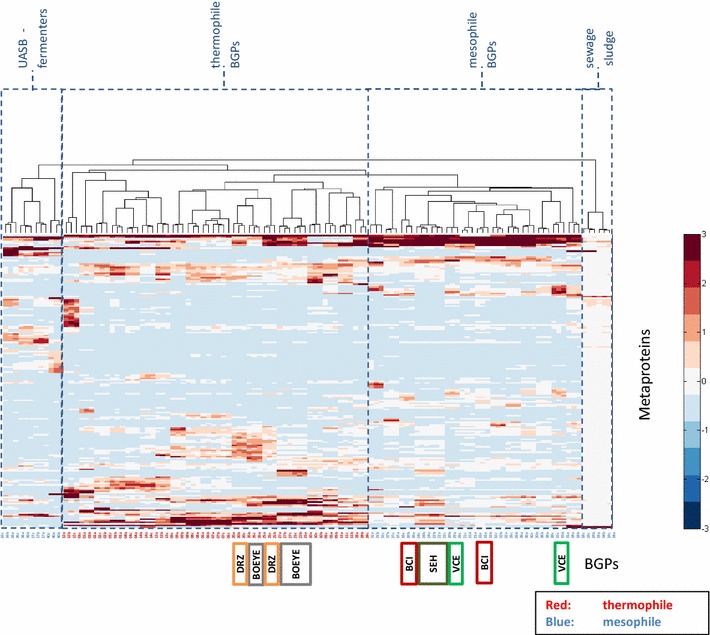
Clustered heat map of all BGPs and their metaprotein profiles generated by hierarchical cluster analysis using the Matlab function “clustergram”. The numbers of metaproteins were normalized to have a mean of 0 and a standard deviation of 1 (see also *color key right* of the figure). Names of BGPs are below the figure, whereas *blue names* refer to BGPs operating with mesophilic and *red names* to BGPs operating with thermophilic process conditions. The *colored groups* are samples from the same BGP at different time points (*dark green* SEH) parallel fermenters [Gent 22 (2012-04-04), Gent 24 (2012-04-04)]; *green* VCE [Gent 15 (2011-10-10), Gent 29 (2012-04-10)]; *gray* BOEYE [Gent 23 (2012-04-14), Gent 27 (2012-04-11), Gent 35 (2011-09-29)]; *orange* DRZ [Gent 20 (2011-09-29), Gent 25 (2012-04-04)]; *red* BCI [Gent 33 (2012-04-11), Gent 39 (2011-09-29)]. After visual assignment, four main clusters of microbial communities are proposed, namely UASB fermenters, thermophile BGPs, mesophile BGP as well as BGPs running with sewage sludge

In comparison to 16S rRNA gene sequencing, metaproteomics provides more extensive data sets for statistical analysis, in particular with respect to the function of the identified metaproteins. Thus, it is to be expected that BGPs can be separated into different clusters correlating with reactor types, substrates or process conditions. In our study, hierarchical clustering analysis based on metaproteins (Fig. 1) resulted in five to six branches which could be combined to four main groups: UASB reactors, sewage sludge as substrate, mesophile, and thermophile process conditions (*p* < 0.01 [22]).

As expected, the clustered heat map (Fig. 1) revealed a close correlation of the triplicates. BGP 06 and BGP 07 formed one branch, which may be explained by similar process parameters. Furthermore, a BGP (SEH, Gent 20/Gent22), which uses two fermenters operating in parallel clustered in one branch. Taken together, these results suggest good reproducibility of the whole workflow including sampling, protein extraction, and LC–MS/MS. BGPs represented by at least two sampling time points (BGP: VCE, BCI, BOEYE, DRZ) also showed some similarity. However, these BGPs did not cluster in linked branches as reported previously for same BGPs but different sampling time points by Heyer et al. [23] or Werner et al. [9]. This could be due to differences in process conditions influencing the community composition and the metabolic activity as described by Theuerl et al. [5]. Indeed, the volatile acid content for these samples changed drastically [from 5735 to 0 mg_COD_/L for BGP VCE (Gent 15/Gent 29) or from 5593 to 22,601 mg_COD_/L for BGP BCI (Gent 33/Gent 39)].

**Fig. 2:**
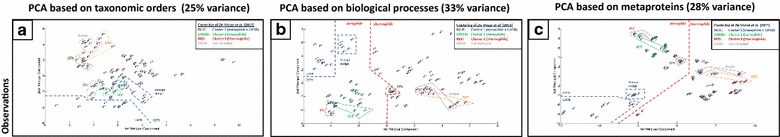
Principal component analyses of all BGPs based on taxonomic order (**a**), biological processes (**b**) and metaproteins(**c**). *Colored groups* are explained in the legend of Fig. 1. Also the clusters of De Vrieze et al. [1] are marked by *blue*, *green* or *red dots*

The four major groups of microbial communities revealed by cluster analysis were also identified by principal component analysis (PCA). However, the two main components of the PCAs for taxonomic orders (Fig. 2a), biological processes (Fig. 2b), and the metaproteins (Fig. 2c) explained only 25, 33 and 28 % of the variances, respectively. Probably, these low values are caused by the very high number of metaproteins, taxonomic orders, and biological processes considered in the statistical analysis as well as the large biological variation between individual plants. Despite this, the plots confirmed a high similarity of triplicates as well as a low sample to sample variation at different sampling time points from the same BGP. Furthermore, visual inspection of the loading plots of the PCAs (Additional file 8: Figure S1) allows for linking the four clusters identified with certain metaproteins, taxonomic orders, and biological processes. For example, more metaproteins related to methanogenesis pathways and to cellular transport were observed in mesophile BGPs, which accorded with the fact that at mesophilic temperature methane is produced by several different pathways (acetoclastic, hydrogenotroph, methylotroph) and at thermophilic conditions mainly by the hydrogenotrophic pathway [24]. In contrast, more metaproteins related to DNA recombination, DNA repair and amino acid biosynthesis were identified in thermophile BGPs. Comparing the results with a PCA based on 16S rRNA taxonomic data [1], the observed groups were also rather similar. Only the separation of mesophile and thermophile BGPs by metaproteome-based taxonomic orders was insufficient (Fig. 2c). However, taxonomic information extracted from metaproteomics experiments is not as accurate as data obtained from 16S rRNA sequencing [1] due to the shorter length of tryptic peptides in comparison to fragments of sequenced 16S rRNA genes.

### Significance of microbial community indices

Microbial community indices, such as the number of species (richness) and the equitability of these species (evenness) [25–27], are widely applied to characterize ecosystem function and stability. So far, however, the use of taxonomy-based metaproteome data was not considered for the estimation of both indices. In the following, we test whether both indices show correlations with process parameters.

**Fig. 3:**
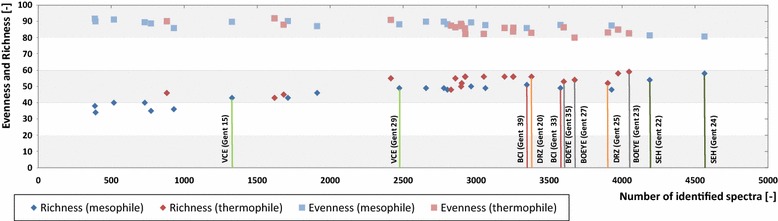
Microbial richness and evenness with increasing number of identified spectra. Evenness is computed as the Gini Index, ranging in value between 0 and 100. *Colored groups* are explained in the legend of Fig. 1

The richness index of samples from BGPs increased with the number of identified spectra from 38 to 58 taxonomic microbial orders and reached a saturation level above 4000 spectra (Fig. 3). In parallel, the evenness index slightly decreased from 92 to 81 % (Gini Index) [25–27]. Thus, a minimal number of approximately 4000 identified spectra seems to be required for the description of microbial communities of BGPs.

Comparison of richness and evenness index for different time points of similar BGPs revealed a high similarity for the BGP SHE (Gent 22, Richness/Gini Index: 34/70.1 %; Gent 24, Richness/Gini Index: 35/69.4 %) and the DRZ (Gent 20: Richness/Gini Index: 33/73.1 %; Gent 25, Richness/Gini Index: 34/73.1 %). However, both indices differed for the other BGPs, as also shown by De Vrieze et al. [1]. For the investigated BGPs, this was most likely due to changes in volatile fatty acids concentrations or process temperatures. In general, higher process temperatures resulted in higher richness and lower evenness indices. This is in contrast to the results obtained by Leven et al. [7], who reported a lower richness index at higher temperatures. This may be caused by a systematic bias of metaproteomics data, i.e., as metaproteomic approaches tend to overestimate high abundant proteins and corresponding species. Probably, the richness index is underestimated in mesophile BGPs due to the high number of taxonomic orders below detection limit. Thus, both indices have to be interpreted with care when applied for the description of microbial communities based on metaproteome data.

### Structure of the microbial community

Microbial communities in well-operating BGPs are often at steady state [6]. The structure of the community represents the taxonomic groups required for conversion of the complex substrate to biogas. Sampling a large number of BGPs might answer the question which microorganisms are essential for stable processes, thus representing a core biogas microbiome [28]. In our study, 34.2 % of the identified spectra (median of all BGPs) were assigned to *Archaea* and 67.78 % to *Bacteria* on the superkingdom level, which fitted to the results of a previous metatranscriptome study [12]. The lowest amount of *Archaea* (11.9 %) was found in BGP Gent 05 and the highest amount (77.7 %) in BGP Gent 16. In contrast, the abundance of 16S rRNA genes of *Archaea* and *Bacteria* identified by De Vrieze et al. [1] was significantly different. Only 1 % *Archaea* (median) were identified with a minimum of 0.18 % and a maximum of 48 %. Such strong differences between the abundance of *Bacteria* and *Archaea* have been observed before, and are probably caused by a methodical bias, for example, 4 % *Archaea* (metagenomics) versus 20–30 % (metaproteomics) [15] or 0.2/7 % *Archaea* (qPCR) versus 12/6 % (microscopy) [29]. Interestingly, the Anaerobic Digestion Model 1 (ADM1) predicts 30–40 % of *Archaea* in mesophile, agricultural CSTR-BGPs [30]. For some BGPs, in particular with lower numbers of identified spectra (less than 4000 identified spectra), the amount of *Archaea* seems to be overestimated, suggesting again that a minimal number of identified spectra is essential for the assignment of meaningful taxonomy profiles (see estimation of community indices above).

**Fig. 4:**
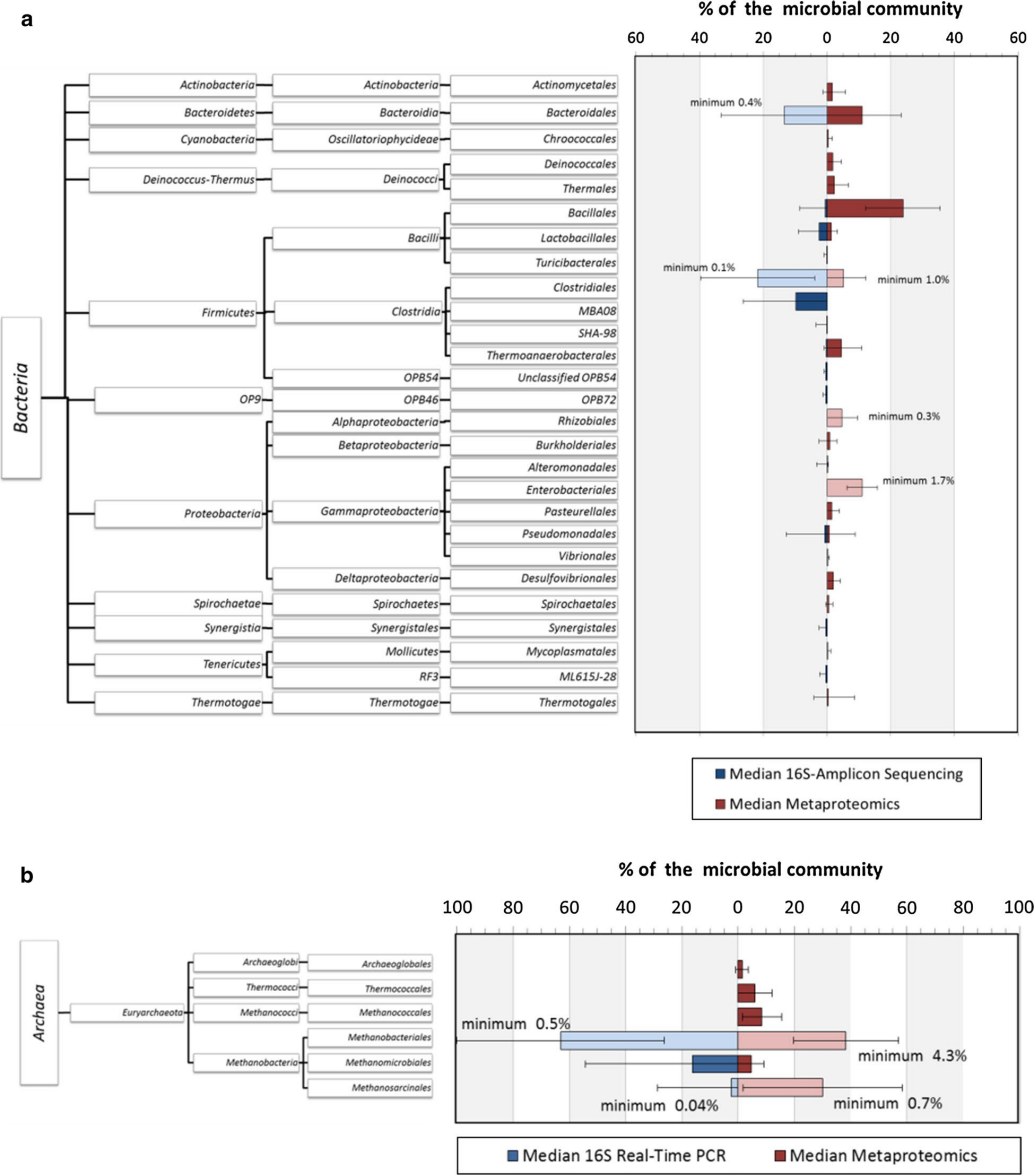
Bacterial (**a**) and archaeal (**b**) taxonomy profiles on the order level based on metaproteome data and genetic analyses of the 16S rRNA for all BGPs. Calculations of the *error bars* were carried out using the standard deviation for all taxonomic orders of all BGPs. Additionally, for all profiles, the core elements identified in all BGPs are shown in *light red* (*blue*) and labeled with the associated minimum value. Unlabeled and *dark red* (*blue*) *bars* indicate that these taxonomic orders were not observed in all BGPs

On the order level, bacterial profiles were dominated by *Bacillales (23.8* *%), Enterobacteriales (11.1* *%), Bacteriodales (11.1* *%), Clostridiales (5.1* *%), Rhizobiales (4.7* *%),* and *Thermoanaerobacteriales (4.6* *%)* (Fig. 4a), and archaeal profiles comprised *Methanobacteriales (38.3* *%), Methanosarcinales (30.1* *%)* and *Methanococcales (8.4* *%)* (Fig. 4b). The archaeal group *Methanococcales* was not observed in the work of De Vrieze et al. [1], but it was detected in other genome-based studies, for example by Stolze et al. [31]. The corresponding genome-based taxonomic profiles identified by De Vrieze et al. [1] were dominated by *Clostridia* (*Clostridiales* 21.8 %, MBA08 9.8 %) and *Bacteriodales* (13.5 %), resp. *Methanobacteriales* (63.2 %). Similar differences between the taxonomic profiles based on metagenomics and metaproteomics approaches were reported previously by Hanreich et al. [15].

The observed differences in the taxonomy profiles based on 16S rRNA amplicon sequencing and metaproteomics could be caused by methodical biases. In particular, cell lysis and the yield of extractions for genomic and proteomic approaches are different. Moreover, genetic methods might be biased by variations of primer affinities to target genes and differences in copy numbers of the 16S-RNA genes [32]. In contrast, metaproteomics-based taxonomy profiles are limited by the presence of highly conserved sequences of identified peptides that prevent the assignment of approximately 50 % of the peptides to a certain order, so far.

**Fig. 5:**
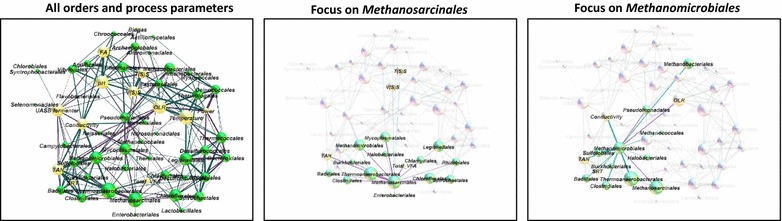
Graph network of taxonomic orders (*green nodes*) and process parameters (*yellow nodes*). Node size depends on the number of edges. *Blue edges* represent positive correlations, *red edges* negative correlation. In the second and third subfigure, only the correlations to *Methanosarcinales* and *Methanomicrobiales* are shown

To identify microbial interactions and correlations of the microorganisms with process parameters, a network of taxonomic orders and all process parameters was created using the software Gephi (Fig. 5). Therefore, all taxonomic orders and process parameters were visualized as nodes and their correlations as edges, followed by a spatial separation based on the connectivity of the nodes and the force atlas algorithm.

Most of the positive correlations between taxonomic orders can be explained by preferences of the corresponding microorganisms for similar process parameter. High temperatures seem to support the enrichment of *Thermotogales, Deinococcales, Thermococcales and Spirochaetales. S*everal positive correlations for *Methanomicrobiales* with, for example, *Clostridiales* or *Thermoanaerobacteriales* were also observed. This correlation corresponds to the role of *Methanomicrobiales* as syntrophic hydrogen consumers [33]. Surprisingly, the hydrogen-consuming order *Methanobacteriales* has only one single positive correlation with *Methanomicrobiales*, which could be explained by similar niche preferences [34]. Furthermore, *Methanosarcinales* showed exclusively negative correlations to other taxonomic orders, for example, to *Clostridiales, Thermoanaerobacteriales*, and *Spirochaetales*. This is most likely due to the fact that *Methanosarcinales* either use acetate or hydrogen for methanogenesis, which makes them independent of other species providing substrates. Instead, negative correlations to TAN and total VFA confirmed their sensitivity for high ammonia concentrations and volatile fatty acids (VFA).

Based on the assumption that independent of the BGP design and of process condition, all major steps of AD are performed; it was tried to identify a core microbiome representing taxonomic orders that are present in all BGPs. This is true for the bacterial orders *Clostridiales*, *Enterobacteriales* and *Rhizobiales* that were identified in all BGPs with at least 1.0, 1.7 and 0.3 % of all spectra, respectively. These low abundances corresponded also with the abundances of core microorganisms calculated based on 16S-RNA amplicon sequencing data, confirming the existence of at least 0.4 % of the order *Bacteroidales* and 0.1 % of the order *Clostridiales* in each BGP (light bars in Fig. 4a). Furthermore, the *archaeal* orders *Methanobacteriales* (4.3 %) and *Methanosarcinales* (0.7 %) were detected in all BGPs by metaproteomics (light bars in Fig. 4b), and also with 0.5 and 0.04 % respectively, by real-time PCR [1]. With respect to the low number of taxonomic core orders identified for all BGPs, the microbial communities were more diverse than expected, reflecting the broad range of substrates and process parameters.

In addition, as clustering and PCA showed four groups of BGPs, microbiomes were separately analyzed (Additional file 9: Table S1). The comparison revealed major differences between groups, and explains the very low number of core orders taking into account for all BGPs. The variations within the groups were smaller and several taxonomic orders could be assigned to specific types of BGPs. For instance, thermophile BGPs were linked with several thermophile orders, such as *Thermotogales* and *Thermococci*, as well as with a reduced number of methanogens. A high proportion of the order *Methanosarcinales,* responsible for acetoclastic methanogenesis, was typical for UASB reactors and BGPs with sewage sludge as substrate. *Syntrophobacteriales,* associated with syntrophic interactions, were specific for UASB reactors, whereas the order *Nitrosomonodales*, performing nitrification [35], was typical for BGPs fed with sewage sludge. On the other hand, the class *Clostridia* was a marker for mesophile BGPs, as well as the methanogenic orders *Methanobacteriales* and *Methanomicrobiales*, previously correlated with mesophile conditions by Nettmann et al. [36]. Accordingly, future attempts to define core microbiomes should focus on the analysis of groups of BGPs with comparable substrate supply and similar process conditions.

### Biological processes and functions

**Fig. 6:**
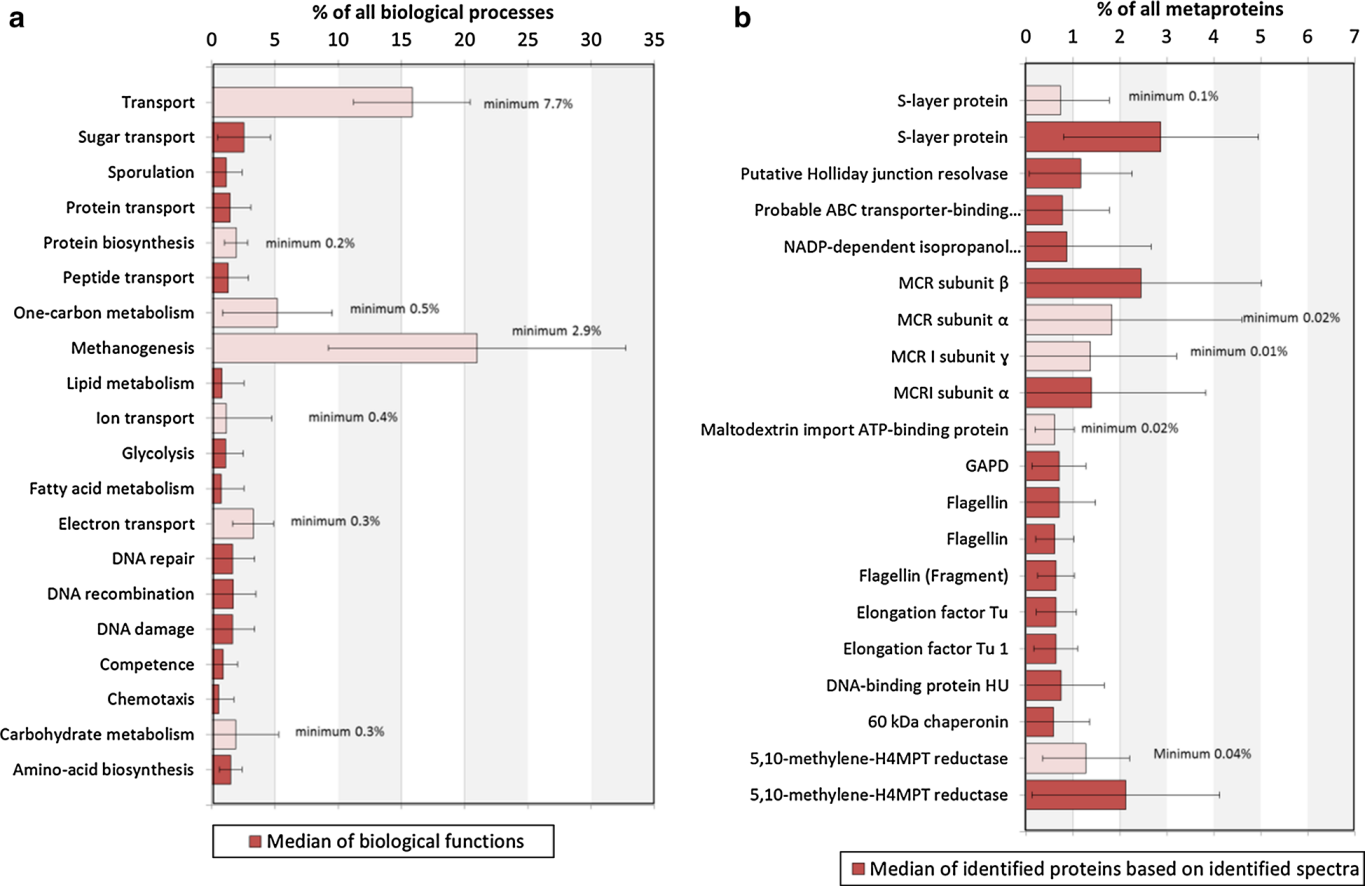
Core community. The top 20 core biological processes (**a**), as well as the top 20 core metaproteins (same UniRef50) (**b**) are shown, based on the number of identified spectra and the median over all BGPs. Calculations of the *error bars* were carried out using the standard deviation of each metaprotein (resp. biological process), calculated for all BGPs. Additionally, for all profiles, the core elements identified in all BGP are shown in *light red* and labeled with the associated minimum value. Unlabeled and *dark red bars* indicate that these biological processes resp. metaproteins were not observed in all BGPs. Two different types of S-layer protein and flagellin were identified in the samples. *GAPD* glyceraldehyde-3-phosphate dehydrogenase, *H*_*4*_ *MPT* tetrahydromethanopterin, *MCR* methyl-coenzyme *M* reductase

The growth of microbial communities as well as conversion of complex substrates to biogas requires a minimum set of biological pathways and cellular functions. These are represented by metaproteins and may be grouped into biological processes. In our study, biological processes were dominated by methanogenesis (median: 21.0 %, min: 2.9 %), followed by transport (median: 15.9 %, min 7.7 %) and one-carbon metabolism (median: 5.2 %, min: 0.5 %) (Fig. 6a). Also, the abundance of biological processes corresponded well with the most abundant metaproteins. Two of the three most abundant metaproteins belonged to methanogenesis, namely methyl-coenzyme M reductase (MCR) (median α: 1.8 + 1.4 %, β: 2.5 %, ɣ: 1.4 %, min: 0.02 %), and 5,10-methylene-tetrahydromethanopterin reductase (5,10-methylene-H_4_MPT reductase) (median: 1. 3/2.1 % min: 0.04 %) (Fig. 6b). Proteins involved in transport and methanogenesis were expressed in all BGPs, ensuring the uptake of substrates and their degradation to methane.

Different process conditions associated with the four clusters of BGPs also correlate with differences in the abundance of metaproteins and biological functions (Additional file 9: Table S2, S3). Thermophile BGPs were dominated by metaproteins for DNA recombination, DNA repair and amino acid biosynthesis, as already observed in the loading plot of the PCA (Fig. 2). Markers for mesophile BGPs were metaproteins for short chain fatty acid metabolism, lipid metabolism and one-carbon metabolism. The identification of specific core functions of BGPs treating sewage sludge or BGPs using UASB reactors was difficult, due to the low total number of metaproteins. Typical for sewage sludge as substrate was nitrate assimilation involving the uptake of inorganic nitrogen that is used as an electron acceptor. Furthermore, a digestive enzyme from human chymotrypsin-like elastase family member IIIA (P09093, K01311) was detected. Although the latter enzyme is not involved in the biogas process, it might be a valuable marker for human feces, as previously proposed by Kuhn et al. [37] and Püttker et al. [38].

**Fig. 7:**
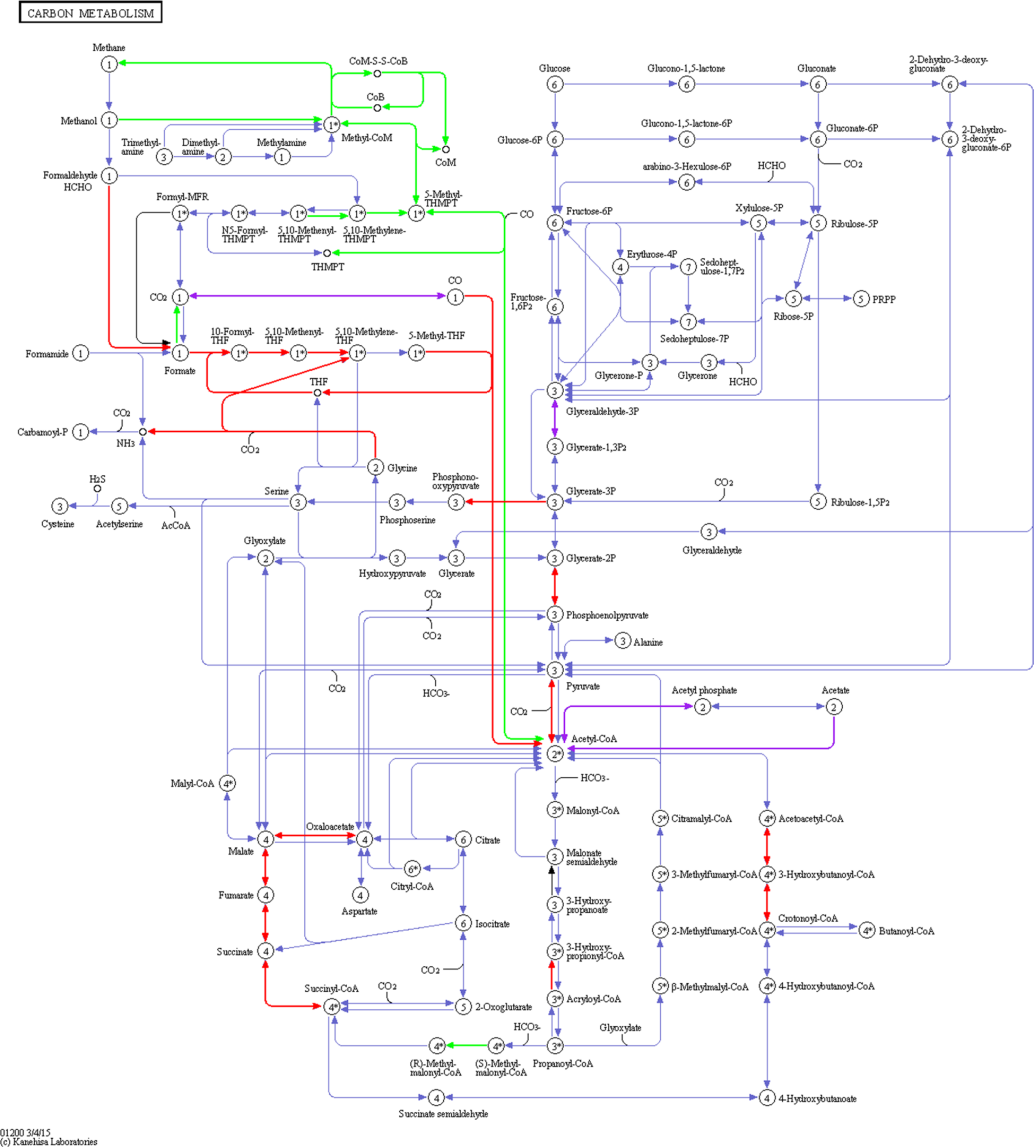
Assignment of identified microbial proteins to the KEGG map 1200 (carbon metabolism) (*green* *Archaea*, *red* *Bacteria*, *purple* metaproteins of *Archaea* and *Bacteria* where the taxonomy of metaproteins could not be assigned on superkingdom level)

Many biological functions identified were linked to cellular metabolism. Therefore, metaproteins were mapped against different metabolic pathways. Best pathway coverage was achieved using the KEGG map 1200 (carbon metabolism) (Fig. 7). Almost all steps of hydrogenotrophic and acetoclastic methanogenesis were observed and assigned to *Methanobacteriales* and *Methanosarcinales*, respectively, as previously described [24, 39–42].

Numerous bacterial folate-dependent enzymes representing one-carbon metabolism were detected that might be involved either in acetogenesis or in syntrophic acetate oxidation (SAO), as also observed by Campanaro et al. [42]. Most of these were assigned to the homoacetogenic species *Moorella thermoacetica* (*Clostridium thermoaceticum)*, which actually performs homoacetogenesis [43]. Only the enzyme formate-tetrahydrofolate ligase was assigned to a syntrophic species (*Syntrophobacteriales*). Probably, the limited genome data of syntrophic acetate oxidizers hampered a correct identification and taxonomic assignment of this functional group that is essential for thermophile BGPs.

Enzymes of the reductive TCA cycle were mainly assigned to *Proteobacteria*, in particular to *E. coli*, involved in the fermentation of pyruvate to succinate. The identification of lactate dehydrogenase (not included in KEGG map 1200 (Fig. 7) assigned to *E. coli* indicates that mixed acid fermentation was also carried out.

In addition to glycolytic enzymes that were mainly assigned to bacteria, several enzymes probably involved in butyrate fermentation were detected. The assignment to *Bacillales* and not to *Clostridiales* is surprising, and might raise questions regarding the reliability of taxonomic assignments on that taxonomic level. In *Clostridiales,* the enzymes amino methyltransferase (P54378, K00605) and glycine reductase complex component B (Q9R4G8, EC: 1.21.4.2) were identified. The first enzyme catalyzes the degradation of glycine to carbon dioxide, ammonia and methylene-THF, which could be further converted to acetate via the Wood-Ljungdahl pathway [44]. The second enzyme enables anaerobic degradation of glycine via the so-called Stickland reactions [not included in KEGG map 1200 (Fig. 7)].

### Correlation of metaproteome data to the process parameters

A main objective of this large-scale analysis of 40 BGPs was to correlate taxonomic and functional data with process parameters. In particular, the temperature, TAN, OLR and SRT showed significant correlations with selected pathways represented in the KEGG map 1200 (carbon metabolism).

Since the influence of process temperature on microbial community has been described previously [24, 45], this parameter was investigated first. High temperature (Additional file 10: Figure S1) correlated with an increased amount of glycolytic enzymes, and lower temperature with a high number of methanogenic enzymes (Additional file 11: Figure S2). The apparent increase of the metaprotein Acetyl-CoA decarbonylase/synthase at higher temperature contradicts the assumption that acetoclastic methanogenesis is not favored. However, only subunit delta 2 of this metaprotein was positively correlated with high temperatures, whereas all others observed subunits of the enzyme remained unchanged.

In addition, enzymes involved in butyrate and propionate fermentation [acyl-CoA dehydrogenase (UniRef50_O32176, EC: 1.3.99.-); 3-ketoacyl-CoA thiolase (UniRef50_O32177, K00632); 3-hydroxyacyl-CoA dehydrogenase (O32178, EC: 1.1.1.35)] were correlated with lower temperature.

High TAN concentrations that are known as a stress factor for BGPs [46] are correlated with increased bacterial one-carbon metabolism as well as enhanced hydrogenotrophic methanogenesis by the families *Methanobacteriaceae* and *Methanosarcinaceae* (Additional file 12: Figure S3) [47]. Both of these biological functions hint to syntrophic acetate oxidation (SAO). The taxonomic orders *Clostridiales*, *Thermoanaerobacteriales* and *Methanomicrobiales* were described as key microorganisms involved in SAO [42, 46]. The high abundance of key enzymes involved in hydrogenotrophic methanogenesis (5,10-methylenetetrahydromethanopterin reductase; *Euryarchaeota*) and one-carbon metabolism (Q3Z8K3 formate-tetrahydrofolate ligase; *Syntrophobacter fumaroxidans*) confirmed this hypothesis. The latter enzyme might also function in reverse direction towards homoacetogenesis, but the low number of *Methanosarcinales* identified and the presence of a key enzyme of the acetoclastic methanogenesis (P26692 Acetyl-CoA decarbonylase/synthase complex subunit alpha, *Methanosaeta concilii*) at high TAN indicate that SAO is the preferred reaction. Finally, with decreasing TAN, acetoclastic methanogenesis by *Methanosaeta* seems to become more abundant (Additional file 13: Figure S4).

High OLRs were positively correlated with acetoclastic methanogenesis (*Methanosarcinales*) and bacterial glycolysis (Additional file 14: Figure S5). In contrast, at low OLR (Additional file 13: Figure S4) hydrogenotrophic methanogenesis and bacterial one-carbon metabolism were increased.

Similar to the low OLR (Additional file 15: Figure S6), a high SRT was linked to hydrogenotrophic methanogenesis and bacterial one-carbon metabolism (Additional file 16: Figure S7). In contrast, a low SRT was correlated with acetoclastic methanogenesis (Additional file 17: Figure S8). Both high SRT and low OLR are supporting SAO, which is in accordance with low growth rates of bacteria carrying out SAO. However, in case of opposite conditions these bacteria seem to be washed out from BGP [48].

Finally, the available data were used to identify single parameters as markers for certain process conditions. Using decision tree learning, (Additional file 18: Table S1), three potential markers were identified: (i) 5,10-methylenetetrahydromethanopterin reductase (Q8TXY4 *Euryarchaeota*) for high TAN, (ii) the order *Thermotogales* for high process temperature [49], and (iii) a decrease of MCR [P07962 MCR subunit alpha (*Methanosarcina barkeri str. Fusaro*)] for high OLR. The latter enzyme has been already proposed previously as a marker for methanogenesis [23, 50]. However, the lack of markers for many other process conditions indicates that more BGPs should be sampled or additional data (e.g., higher depth of metaproteome analysis) should be included in the future studies. Furthermore, the applied classification thresholds for the individual process parameters strongly influenced the identification of the markers. In most cases, classification thresholds were taken from the literature [51–53]; otherwise, medians of the process parameters were applied. Unfortunately, threshold values of classifications reported in the literature often vary significantly. For instance, for the classification of BGPs with regard to TAN, Chen et al. [52] applied a threshold of 4200 mg/L whereas Schnürer et al. [53] used a threshold of 3000 mg/L. Using this lower threshold for classification, the reliability of 5,10-methylenetetrahydromethanopterin reductase (Q8TXY4 *Euryarchaeota*) as an indicator for high TAN was increased, as indicated by the lower error rate for the classification (data not shown).

## Conclusions

In this study, the first large-scale proteotyping of 40 BGP samples was conducted. The optimized workflow established for the investigation of microbial communities in BGPs did not require extensive pre-fractionation of samples, but achieved a high coverage of proteins by applying sensitive Orbitrap-MS, and searching spectra against BGP metagenomes using a comprehensive bioinformatics platform.

The results of the proteotyping enabled the clustering of data of biogas processes to identify (i) UASB fermenters, (ii) feeding of sewage sludge as substrate, (iii) mesophile, and (iv) thermophile process conditions, as previously reported by De Vrieze et al. [1] using a 16S rRNA gene sequencing approach. Based on functional analysis, TAN, SRT, OLR, and temperature were identified as key parameters determining the composition and function of microbial communities.

Although observed correlations (e.g., for high TAN and hydrogenotrophic methanogenesis) were mainly related to SAO and methanogenesis, metaproteome analysis has the potential for answering major ecological questions and for monitoring of the health of BGPs. Proteotyping BGPs in the follow-up studies should include the analysis of (i) healthy BGPs over longer periods, (ii) similar BGPs with different process conditions (e.g., feed composition, OLR) and (iii) ‘sick’ BGPs with severe process disturbances (e.g., acidification, foaming). The approach could filter out more specific core taxonomies and core functions than presented in this paper. Based on these systematic studies, metaproteins or taxonomies could be identified as biomarkers. If the abundance of these biomarkers is rapidly changing or behave contradictory to selected process parameters, this might be a sign of (future) process failure.

## Methods

The complete workflow included experimental and computational steps (Additional file 19: Figure S1). All chemicals used for the different methods were of analysis grade or higher, and for LC–MS/MS measurements only MS grade chemicals were used.

### Sampling and analytics

With the exception of four samples (BIE2, Oss2, Oss3, BCI3), the same samples of the BGP used in the study of De Vrieze et al. [1] were analyzed. However, samples from additional BGPs were taken into account resulting in a total number of 40 BGP samples. The pH values were measured directly after sampling. The TAN, volatile solids (VS) and total solids (TS), and the conductivity were measured after storage at 4 °C, and VFA concentration after storage at −20 °C [1]. The plant operators provided information concerning the OLR, SRT, biogas production, temperature, reactor type (CSTR/UASB) and volume, as well as on the substrate composition (Additional file 1: Table S1, S2).

### Metaproteomics

Protein extraction from digestate stored at −20 °C was carried out by phenol extraction as described by Heyer et al. [23]. Proteins were dissolved in a solution containing 7 M urea, 2 M thiourea as well as 0.01 g/mL 1,4-dithiothreitol, and quantified with an amido black assay [54]. For each sample, 200 µg of protein was precipitated by acetone, and separated by SDS-PAGE [55]. To pre-purify samples for MS/MS, 200 µg of proteins was loaded onto a SDS-PAGE but the separation was stopped after the proteins entered approximately 5 mm into the separation gel. The gel slices containing the entered proteins were digested with trypsin to obtain peptides [24].

Peptides were analyzed by LC–MS/MS using an UltiMate 3000 RSLCnano splitless liquid chromatography system, coupled online to an Orbitrap Elite™ Hybrid Ion Trap-Orbitrap MS (both from Thermo Fisher Scientific, Bremen, Germany). After injection, peptides were loaded isocratically on a trap column (Dionex Acclaim, nano trap column, 100 μm i.d. × 2 cm, PepMap100 C18, 5 μm, 100 Å, nanoViper) with a flow rate of 7 μL/min chromatographic liquid phase A (98 % LC–MS Water, 2 % ACN, 0.05 % TFA) for desalting and concentration.

Chromatographic separation was performed on a Dionex Acclaim PepMap C18 RSLC nano reversed phase column (2 μm particle size, 100 Å pore size, 75 μm inner diameter and 250 mm length) at 40 °C column temperature. A flow rate of 300 nL/min was applied using a binary A/B-solvent gradient (solvent A: 98 % LC–MS Water, 2 % acetonitrile, 0.1 % formic acid; solvent B: 80 % acetonitrile, 10 % LC–MS Water, 10 % trifluorethanol, 0.1 % formic acid) starting with 4 % B for 4 min, continuing with a linear increase to 55 % B within 120 min, followed by a column wash with 90 % B for 5 min and a re-adjusted with 4 % B for 25 min. For MS acquisition, a data-dependent MS/MS method was chosen. For the conducted measurements, MS was operated in positive ion mode, and precursor ions were acquired in the orbital trap of the hybrid MS at a resolution of 30,000 and an m/z range of 350–2000. Subsequently, the fragment ion scan was proceeded in the linear ion trap of the hybrid MS with a mass range and a scan rate with “normal” parameter settings for the top 20 most intense precursors selected for collision-induced dissociation.

### Data handling

The MS results were processed by the Proteome Discoverer Software 1.4 (Thermo Fisher Scientific, Bremen, Germany), and were exported as Mascot generic format *(.mgf)*. For data storing and database search with the MASCOT 2.5 software (Matrix Science, London, England) [56], the *mgf*-files were imported into the ProteinScape software (Bruker Daltonics, Bremen, Deutschland, version 3.1.3.461). The following search parameters were applied: trypsin, one missed cleavage, monoisotopic mass, carbamidomethylation (cysteine) and oxidation (methionine) as variable modifications, ±10 ppm precursor and ±0.5 Da MS/MS fragment tolerance, 1^13^C and +2/+ 3 charged peptide ions, 1 % FDR (resp. Mascot Score of 40 for Fig. 2). As protein database UniProtKB/Swiss-Prot (version: 23.10.2014) [57] extended by seven metagenomes [11, 15, 19, 20] was used. The results of database search were submitted to PRIDE [58] with the accession number PXD003526.

Mascot result files (.*dat*-*files*) were uploaded into an extended version of the MPA Software [18] (https://www.code.google.com/p/meta-proteome-analyzer/, version 1.0.9) to add meta-information from the UniProtKB/Swiss-Prot database concerning taxonomy and function (UniProtKB gs: biological process, Enzyme Commission numbers (EC-number) [59] and KEGG Orthology (KO) [60]). The extended version of the MPA implements a BLAST search (NCBI-Blast-version 2.2.31 [61]) against the UniProtKB/Swiss-Prot database for non-annotated sequences from metagenomes. The UniProtKB accession numbers of first hits (*e* value better than 10^−4) were assigned to the hits from the metagenomes. Redundant protein identifications were grouped by the UniRef50 Clusters [21] to the so-called “metaproteins”. The taxonomy of each metaprotein was redefined to the common ancestor taxonomy of all peptides grouped to this metaprotein. The metaproteins, taxonomy profiles on order level and microbial biological process profiles (UniProtKB keywords) of each BGP and their spectral abundance were exported as comma separated files.

### Biostatistics

The software Matlab [The MathWorks GmbH, Ismaning, Germany, version 8.3.0.532 (R2014a)] was used with the statistic toolbox to identify correlations as well as patterns in the microbial community and its biological functions (Additional files 20, 21, 22). First, fusion matrices of the metaproteins resp. taxonomic orders or biological processes of all BGPs were generated. Second, unknown hits and contaminant keratin hits were excluded. Third, the abundances of spectra of metaproteins from each BGP were normalized to 100 % of the total number of spectra of that BGP. Finally, all matrices were filtered for entries, which were present in at least one BGP sample with at least 1 % of the spectra.

These matrices were used to investigate the similarity of the BGPs based on a hierarchical clustering analysis. The results are shown as clustered heat map (Matlab: “clustergram” (“Bioinformatics Toolbox”), distance “average”, linkage “euclidian” + Matlab “PermTest_cluster_comparison”, number of replications “1000”, for bootstrapping [22]) and as PCA [Matlab: “pca” (“Statistics and machine learning toolbox”)].

The correlations of all variables with each other and with the process parameters were analyzed by the “corr”-function (Matlab “Statistics Toolbox”) applying the Spearman’s rank and *p* values of 0.05 and 0.01, respectively. For visualization of correlations, a graph network was created based on correlations between the taxonomic orders and process parameters using Gephi (version: 0.8.2.beta) [62] and the force atlas algorithm (autostab strength “2000”, repulsion strength “1000”, attraction strength “1”, gravity “100”, attraction distrib. “checked”).

For the search of potential markers for high and low process parameters on the level of metaproteins, taxonomic orders or biological processes, decision tree learning was applied [Matlab: “classregtree” (“Statistics and machine learning toolbox”)]. Therefore, the BGPs were classified in BGPs with high resp. low values of each process parameter and the decision tree algorithm was used to propose marker metaproteins, taxonomic orders or biological processes which explained the classification of the BGPs. Finally, the performance of the decision tree learning was investigated by randomly splitting the dataset into two subsets for training (60 %) and testing (40 %).

The community indices evenness and richness were calculated based on 1 % order profiles with in-house excel sheets (Additional file 23) [25–27].

## Additional files

10.1186/s13068-016-0572-4 S1: Process parameters of the analyzed BGPs. Different substrate types marked by the different colors (green: agricultural substrates, orange: industrial waste, purple: slaughter house waste, blue: sewage sludge, red: municipal waste, grey: mixed/unknown substrates. The column labeled with “Name*” corresponds to the BGP names in De Vrieze et al. [1]. S2: Correlations between process parameters visualized as generalized plot matrix [63].
10.1186/s13068-016-0572-4 12 % SDS-PAGE of all 40 BGPs loaded with 200 µg of proteins and stained with colloidal coomassie.
10.1186/s13068-016-0572-4 Number of identified spectra for each BGP (average of triplicates). Each sample was searched against UniProtKB/Swiss-Prot and UniProtKB/Swiss-Prot including several metagenomes, applying a Mascot score of 40 and a FDR of 1 %.
10.1186/s13068-016-0572-4 Application of different cut-offs for metaprotein, taxonomic order and biological process matrices. In addition the average cumulative sum of identified spectra is shown for different cut-offs.
10.1186/s13068-016-0572-4 Detailed description of protein identifications and list of all metaproteins, orders and biological processes.
10.1186/s13068-016-0572-4 S1: List of all identified metaproteins. S2: List of all identified taxonomic orders. S3: List of all identified biological processes.
10.1186/s13068-016-0572-4 S1: Matrix of all correlations with a cut-off of 0.01. S2: Matrix of all correlations with a cut-off of 0.05.
10.1186/s13068-016-0572-4 Loading Plots for the PCAs shown in Fig. 2.
10.1186/s13068-016-0572-4 S1: Microbial orders for the four main groups of BGPs, namely mesophile and thermophile BGPs, UASB reactors and sewage sludge BGPs. S2: Biological functions of the four main groups of BGPs, namely mesophile and thermophile BGPs, UASB reactors and sewage sludge BGPs. S3: Metaproteins of the four main groups of BGPs, namely mesophile and thermophile BGPs, UASB reactors and sewage sludge BGPs.
10.1186/s13068-016-0572-4 Carbon metabolism at high temperatures. Assignment of identified microbial proteins to the KEGG map 1200 (carbon metabolism) positively correlated with high temperature (green: *Archaea*, red: *Bacteria*, purple: *Archaea* or *Bacteria*).
10.1186/s13068-016-0572-4 Carbon metabolism at low temperatures. Assignment of identified microbial proteins to the KEGG map 1200 (carbon metabolism) negatively correlated with high temperature (green: *Archaea*, red: *Bacteria*, purple: *Archaea* or *Bacteria*).
10.1186/s13068-016-0572-4 Carbon metabolism at high TAN. Assignment of identified microbial proteins to the KEGG map 1200 (carbon metabolism) positively correlated with high TAN (green: *Archaea*, red: *Bacteria*, purple: *Archaea* or *Bacteria*).
10.1186/s13068-016-0572-4 Carbon metabolism at low TAN. Assignment of identified microbial proteins to the KEGG map 1200 (carbon metabolism) negatively correlated with high TAN (green: *Archaea*, red: *Bacteria*, purple: *Archaea* or *Bacteria*).
10.1186/s13068-016-0572-4 Carbon metabolism at high OLR. Assignment of identified microbial proteins to the KEGG map 1200 (carbon metabolism) positively correlated with high OLR (green: *Archaea*, red: *Bacteria*, purple: *Archaea* or *Bacteria*).
10.1186/s13068-016-0572-4 Carbon metabolism at low OLR. Assignment of identified microbial proteins to the KEGG map 1200 (carbon metabolism) negatively correlated with high OLR (green: *Archaea*, red: *Bacteria*, purple: *Archaea* or *Bacteria*).
10.1186/s13068-016-0572-4 Carbon metabolism at high SRT. Assignment of identified microbial proteins to the KEGG map 1200 (carbon metabolism positively correlated with high SRT (green: *Archaea*, red: *Bacteria*, purple: *Archaea* or *Bacteria*).
10.1186/s13068-016-0572-4 Carbon metabolism at low SRT. Assignment of identified microbial proteins to the KEGG map 1200 (carbon metabolism) negatively correlated with high SRT (green: *Archaea*, red: *Bacteria*, purple: *Archaea* or *Bacteria*).
10.1186/s13068-016-0572-4 Potential markers of process conditions identified by decision tree learning. The classification errors as well as the correlations to process parameters are shown (cut-off 5 %, Spearman’s rank, *p* value 0.01).
10.1186/s13068-016-0572-4 Workflow.
10.1186/s13068-016-0572-4 Matlab files for the statistical data evaluation. In order to execute the statistical data evaluation run the file “Additional_File_20_Start_Data_Analysis.m”. After that you have to select subsequently all files for the taxonomic orders (“Additional_Files_20_Taxonomic_Order_UniRef50_allSpecies_GentXXx.csv”), biological processes (“Additional_Files_20_Gent_Biological_Process_UniRef50_allSpecies_GentXXx.csv”) as well metaproteins (“Additional_Files_20_Gent_Metaproteins_UniRef50_allSpecies_GentXXx.m”).
10.1186/s13068-016-0572-4 Analogous, decision tree learning can be execute by running the file “Additional_File_21_Start_DecissionTrees.m” and selecting the desired taxonomy matrix (“Additional_Files_21_Taxonomic_Order_Learning”), biological process matrix (“Additional_Files_21_Biological_Processes_Learning”) or metaprotein matrix (“Additional_Files_21_Metaprotein_Learning”).
10.1186/s13068-016-0572-4 Summary.
10.1186/s13068-016-0572-4 Calculations sheet for richness and evenness.

## Abbreviations

BGP(s): biogas plant(s)
CID: collision induced dissociation
COD: chemical oxygen demand
CSTR: continuously stirred tank reactor
FDR: false discovery rate
EC-number: Enzyme Commission number
FA: free ammonia
GAPD: glyceraldehyde-3-phosphate dehydrogenase
H_4_MPT: tetrahydromethanopterin
KO: KEGG orthology
LC: liquid chromatography
MCR: methyl-coenzyme M reductase
*mgf*: mascotgeneric file
MPA: MetaProteomeAnalyzer
MS: mass spectrometry/mass spectrometer
MS/MS: tandem mass spectrometry/tandem mass spectrometer
PCA: principal component analysis
PCR: polymerase chain reaction
OLR: organic loading rate
SAO: syntrophic acetate oxidation
SDS-PAGE: sodium dodecyl sulfate polyacrylamide gel electrophoresis
SRT: sludge retention time
TAN: total ammonia nitrogen
TS: total solids
UASB: upflow anaerobic sludge blanket reactor
VFA: volatile fatty acids
VS: volatile solids

## References

[1] De Vrieze J, Saunders AM, He Y, Fang J, Nielsen PH, Verstraete W (2015). Ammonia and temperature determine potential clustering in the anaerobic digestion microbiome. Water Res.

[2] Fachagentur Nachwachsende Rohstoffe e.V. Basisdaten Bioenergie. 2015. https://mediathek.fnr.de/basisdaten-bioenergie.html. Accessed 15 Jan 2016.

[3] Stams AJM, Plugge CM (2009). Electron transfer in syntrophic communities of anaerobic bacteria and archaea. Nat Rev Microbiol.

[4] Bagi Z, Acs N, Balint B, Horvath L, Dobo K, Perei KR (2007). Biotechnological intensification of biogas production. Appl Microbiol Biotechnol.

[5] Theuerl S, Kohrs F, Benndorf D, Maus I, Wibberg D, Schluter A (2015). Community shifts in a well-operating agricultural biogas plant: how process variations are handled by the microbiome. Appl Microbial Biotechnol.

[6] Lucas R, Kuchenbuch A, Fetzer I, Harms H, Kleinsteuber S (2015). Long-term monitoring reveals stable and remarkably similar microbial communities in parallel full-scale biogas reactors digesting energy crops. FEMS Microbiol Ecol.

[7] Leven L, Eriksson ARB, Schnurer A (2007). Effect of process temperature on bacterial and archaeal communities in two methanogenic bioreactors treating organic household waste. FEMS Microbiol Ecol.

[8] De Vrieze J, Verstraete W, Boon N (2013). Repeated pulse feeding induces functional stability in anaerobic digestion. Microb Biotechnol.

[9] Werner JJ, Knights D, Garcia ML, Scalfone NB, Smith S, Yarasheski K (2011). Bacterial community structures are unique and resilient in full-scale bioenergy systems. Proc Natl Acad Sci USA.

[10] Ziganshin AM, Liebetrau J, Proter J, Kleinsteuber S (2013). Microbial community structure and dynamics during anaerobic digestion of various agricultural waste materials. Appl Microbiol Biotechnol.

[11] Schlüter A, Bekel T, Diaz NN, Dondrup M, Eichenlaub R, Gartemann KH (2008). The metagenome of a biogas-producing microbial community of a production-scale biogas plant fermenter analysed by the 454-pyrosequencing technology. J Biotechnol.

[12] Zakrzewski M, Goesmann A, Jaenicke S, Junemann S, Eikmeyer F, Szczepanowski R (2012). Profiling of the metabolically active community from a production-scale biogas plant by means of high-throughput metatranscriptome sequencing. J Biotechnol.

[13] Heyer R, Kohrs F, Reichl U, Benndorf D (2015). Metaproteomics of complex microbial communities in biogas plants. Microb Biotechnol.

[14] Vanwonterghem I, Jensen PD, Ho DP, Batstone DJ, Tyson GW (2014). Linking microbial community structure, interactions and function in anaerobic digesters using new molecular techniques. Curr Opin Biotechnol.

[15] Hanreich A, Schimpf U, Zakrzewski M, Schlüter A, Benndorf D, Heyer R (2013). Metagenome and metaproteome analyses of microbial communities in mesophilic biogas-producing anaerobic batch fermentations indicate concerted plant carbohydrate degradation. Syst Appl Microbiol.

[16] Ong SH, Kukkillaya VU, Wilm A, Lay C, Ho EX, Low L (2013). Species identification and profiling of complex microbial communities using shotgun illumina sequencing of 16S rRNA amplicon sequences. PLoS One.

[17] Gonzalez JM, Portillo MC, Belda-Ferre P, Mira A (2012). Amplification by PCR artificially reduces the proportion of the rare biosphere in microbial communities. PLoS One.

[18] Muth T, Behne A, Heyer R, Kohrs F, Benndorf D, Hoffmann M (2015). The MetaProteomeAnalyzer: a powerful open-source software suite for metaproteomics data analysis and interpretation. J Proteome Res.

[19] Joint Genome Institute PI. http://genome.jgi.doe.gov/BioPla1DNA1/BioPla1DNA1.info.html. Proposal ID 1053. 2015. Accessed 15 Jan 2016.

[20] Rademacher A, Zakrzewski M, Schlüter A, Schonberg M, Szczepanowski R, Goesmann A (2012). Characterization of microbial biofilms in a thermophilic biogas system by high-throughput metagenome sequencing. FEMS Microbiol Ecol.

[21] Suzek BE, Huang HZ, McGarvey P, Mazumder R, Wu CH (2007). UniRef: comprehensive and non-redundant UniProt reference clusters. Bioinformatics.

[22] Pinelli C, Rastogi RK, Scandurra A, Jadhao AG, Aria M, D’Aniello B (2014). A comparative cluster analysis of nicotinamide adenine dinucleotide phosphate (NADPH)-diaphorase histochemistry in the brains of amphibians. J Comp Neurol.

[23] Heyer R, Kohrs F, Benndorf D, Rapp E, Kausmann R, Heiermann M (2013). Metaproteome analysis of the microbial communities in agricultural biogas plants. N Biotechnol.

[24] Kohrs F, Heyer R, Magnussen A, Benndorf D, Muth T, Behne A (2014). Sample prefractionation with liquid isoelectric focusing enables in depth microbial metaproteome analysis of mesophilic and thermophilic biogas plants. Anaerobe.

[25] Marzorati M, Wittebolle L, Boon N, Daffonchio D, Verstraete W (2008). How to get more out of molecular fingerprints: practical tools for microbial ecology. Environ Microbiol.

[26] Mertens B, Boon N, Verstraete W (2005). Stereospecific effect of hexachlorocyclohexane on activity and structure of soil methanotrophic communities. Environ Microbiol.

[27] Wittebolle L, Marzorati M, Clement L, Balloi A, Daffonchio D, Heylen K (2009). Initial community evenness favours functionality under selective stress. Nature.

[28] Shade A, Handelsman J (2012). Beyond the Venn diagram: the hunt for a core microbiome. Environ Microbiol.

[29] Kim YS, Westerholm M, Scherer P (2014). Dual investigation of methanogenic processes by quantitative PCR and quantitative microscopic fingerprinting. FEMS Microbiol Lett.

[30] Batstone DJ, Keller J, Angelidaki I, Kalyuzhnyi SV, Pavlostathis SG, Rozzi A (2002). The IWA anaerobic digestion model no 1 (ADM1). Water Sci Technol.

[31] Stolze Y, Zakrzewski M, Maus I, Eikmeyer F, Jaenicke S, Rottmann N (2015). Comparative metagenomics of biogas-producing microbial communities from production-scale biogas plants operating under wet or dry fermentation conditions. Biotechnol Biofuels.

[32] Acinas SG, Marcelino LA, Klepac-Ceraj V, Polz MF (2004). Divergence and redundancy of 16S rRNA sequences in genomes with multiple rrn operons. J Bacteriol.

[33] Karakashev D, Batstone DJ, Trably E, Angelidaki I (2006). Acetate oxidation is the dominant methanogenic pathway from acetate in the absence of Methanosaetaceae. Appl Environ Microbiol.

[34] Liu YC, Whitman WB (2008). Metabolic, phylogenetic, and ecological diversity of the methanogenic archaea. Ann NY Acad Sci.

[35] Brenner D, Krieg N, Staley J. Bergey’s manual® of systematic bacteriology. The proteobacteria part C: the alpha-, beta-, delta-, and epsilonproteobacteria, vol 2. US: Springer ; 2005.

[36] Nettmann E, Bergmann I, Pramschufer S, Mundt K, Plogsties V, Herrmann C (2010). Polyphasic analyses of methanogenic archaeal communities in agricultural biogas plants. Appl Environ Microbiol.

[37] Kuhn R, Benndorf D, Rapp E, Reichl U, Palese LL, Pollice A (2011). Metaproteome analysis of sewage sludge from membrane bioreactors. Proteomics.

[38] Püttker S, Kohrs F, Benndorf D, Heyer R, Rapp E, Reichl U (2015). Metaproteomics of activated sludge from a wastewater treatment plant—a pilot study. Proteomics.

[39] Kohrs F, Wolter S, Benndorf D, Heyer R, Hoffmann M, Rapp E (2015). Fractionation of biogas plant sludge material improves metaproteomic characterization to investigate metabolic activity of microbial communities. Proteomics.

[40] Lu F, Bize A, Guillot A, Monnet V, Madigou C, Chapleur O (2014). Metaproteomics of cellulose methanisation under thermophilic conditions reveals a surprisingly high proteolytic activity. ISME J.

[41] Bremges A, Maus I, Belmann P, Eikmeyer F, Winkler A, Albersmeier A (2015). Deeply sequenced metagenome and metatranscriptome of a biogas-producing microbial community from an agricultural production-scale biogas plant. Gigascience.

[42] Campanaro S, Treu L, Kougias PG, De Francisci D, Valle G, Angelidaki I (2016). Metagenomic analysis and functional characterization of the biogas microbiome using high throughput shotgun sequencing and a novel binning strategy. Biotechnol Biofuels.

[43] Pierce E, Xie G, Barabote RD, Saunders E, Han CS, Detter JC (2008). The complete genome sequence of Moorella thermoacetica (f. *Clostridium thermoaceticum*). Environ Microbiol.

[44] Okamura-Ikeda K, Fujiwara K, Motokawa Y (1987). Mechanism of the glycine cleavage reaction. Properties of the reverse reaction catalyzed by T-protein. J Biol Chem.

[45] Gunnigle E, Siggins A, Botting CH, Fuszard M, O’Flaherty V, Abram F (2015). Low-temperature anaerobic digestion is associated with differential methanogenic protein expression. FEMS Microbiol Lett.

[46] Müller B, Sun L, Schnurer A (2013). First insights into the syntrophic acetate-oxidizing bacteria—a genetic study. Microbiologyopen.

[47] Kovacs E, Wirth R, Maroti G, Bagi Z, Rakhely G, Kovacs KL (2013). Biogas production from protein-rich biomass: fed-batch anaerobic fermentation of casein and of pig blood and associated changes in microbial community composition. PLoS One.

[48] Archer DB, Powell GE (1985). Dependence of the specific growth rate of methanogenic mutualistic cocultures on the methanogen. Arch Microbiol.

[49] Huber R, Hannig M, Dworkin M, Falkow S, Rosenberg E, Schleifer KH, Stackebrandt E (2006). Thermotogales. The prokaryotes.

[50] Munk B, Bauer C, Gronauer A, Lebuhn M (2012). A metabolic quotient for methanogenic Archaea. Water Sci Technol.

[51] Weiland P (2010). Biogas production: current state and perspectives. Appl Microbiol Biotechnol.

[52] Chen Y, Cheng JJ, Creamer KS (2008). Inhibition of anaerobic digestion process: a review. Bioresour Technol.

[53] Schnürer A, Nordberg A (2008). Ammonia, a selective agent for methane production by syntrophic acetate oxidation at mesophilic temperature. Water Sci Technol.

[54] Popov N, Schmitt M, Schulzeck S, Matthies H (1975). Eine Störungsfreie Mikromethode zur Bestimmung des Proteingehaltes in Gewebehomogenaten. Acta biologica et medica Germanica.

[55] Laemmli UK (1970). Cleavage of structural proteins during the assembly of the head of bacteriophage T4. Nature.

[56] Perkins DN, Pappin DJC, Creasy DM, Cottrell JS (1999). Probability-based protein identification by searching sequence databases using mass spectrometry data. Electrophoresis.

[57] UniProt Consortium (2012). Reorganizing the protein space at the Universal Protein Resource (UniProt). Nucleic Acids Res.

[58] Vizcaino JA, Cote RG, Csordas A, Dianes JA, Fabregat A, Foster JM (2013). The proteomics identifications (PRIDE) database and associated tools: status in 2013. Nucleic Acids Res.

[59] Bairoch A (2000). The ENZYME database in 2000. Nucleic Acids Res.

[60] Kanehisa M, Goto S (2000). KEGG: kyoto encyclopedia of genes and genomes. Nucleic Acids Res.

[61] Shevchenko A, Sunyaev S, Loboda A, Shevehenko A, Bork P, Ens W (2001). Charting the proteomes of organisms with unsequenced genomes by MALDI-quadrupole time of flight mass spectrometry and BLAST homology searching. Anal Chem.

[62] Bastian M, Heymann S, Jacomy M. ICWSM. https://gephi.org/publications/gephi-bastian-feb09.pdf. 2009. Accessed 15 Jan 2016.

[63] Im JF, McGuffin MJ, Leung R (2013). GPLOM: the generalized plot matrix for visualizing multidimensional multivariate data. IEEE Trans Vis Comput Graph.

