# Supplementary figures and images for: Proteotyping of biogas plant microbiomes separates biogas plants according to process temperature and reactor type
Source: Biotechnol Biofuels. 2016 Jul 26;9:155. doi: 10.1186/s13068-016-0572-4 (PMC4960849; doi:10.1186/s13068-016-0572-4)

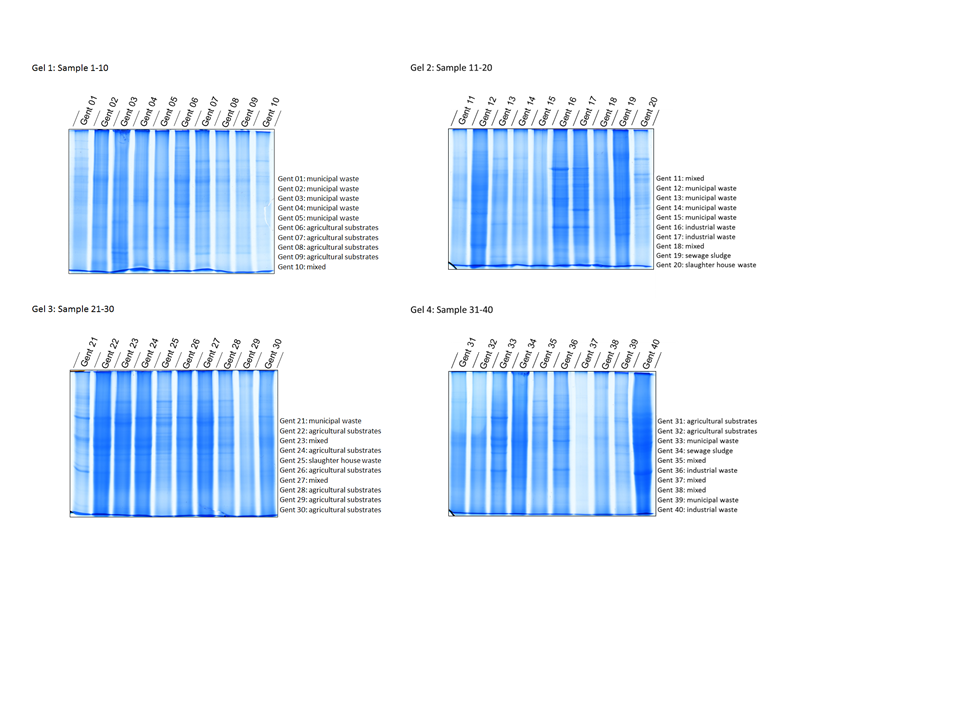

Supplement: Supplementary file 2 — 10.1186/s13068-016-0572-4 12 % SDS-PAGE of all 40 BGPs loaded with 200 µg of proteins and stained with colloidal coomassie. [file 13068_2016_572_MOESM2_ESM.png]

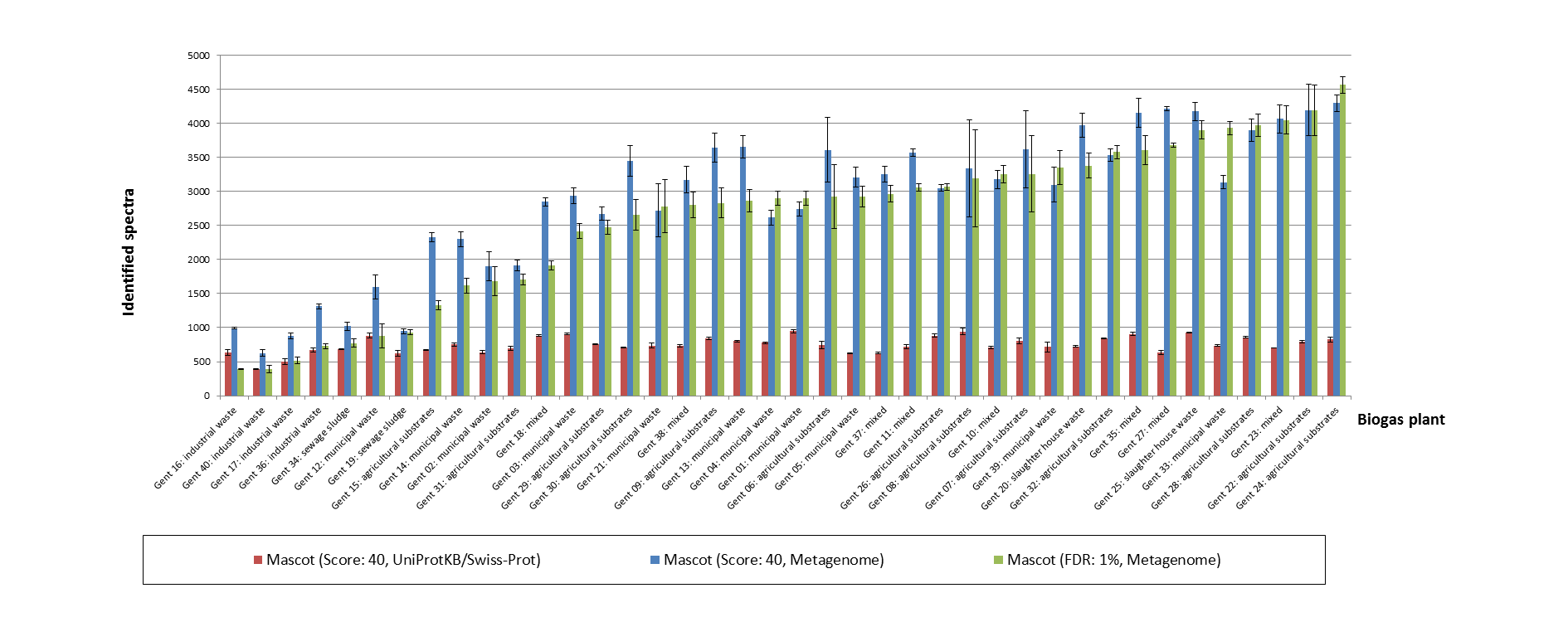

Supplement: Supplementary file 3 — 10.1186/s13068-016-0572-4 Number of identified spectra for each BGP (average of triplicates). Each sample was searched against UniProtKB/Swiss-Prot and UniProtKB/Swiss-Prot including several metagenomes, applying a Mascot score of 40 and a FDR of 1 %. [file 13068_2016_572_MOESM3_ESM.png]

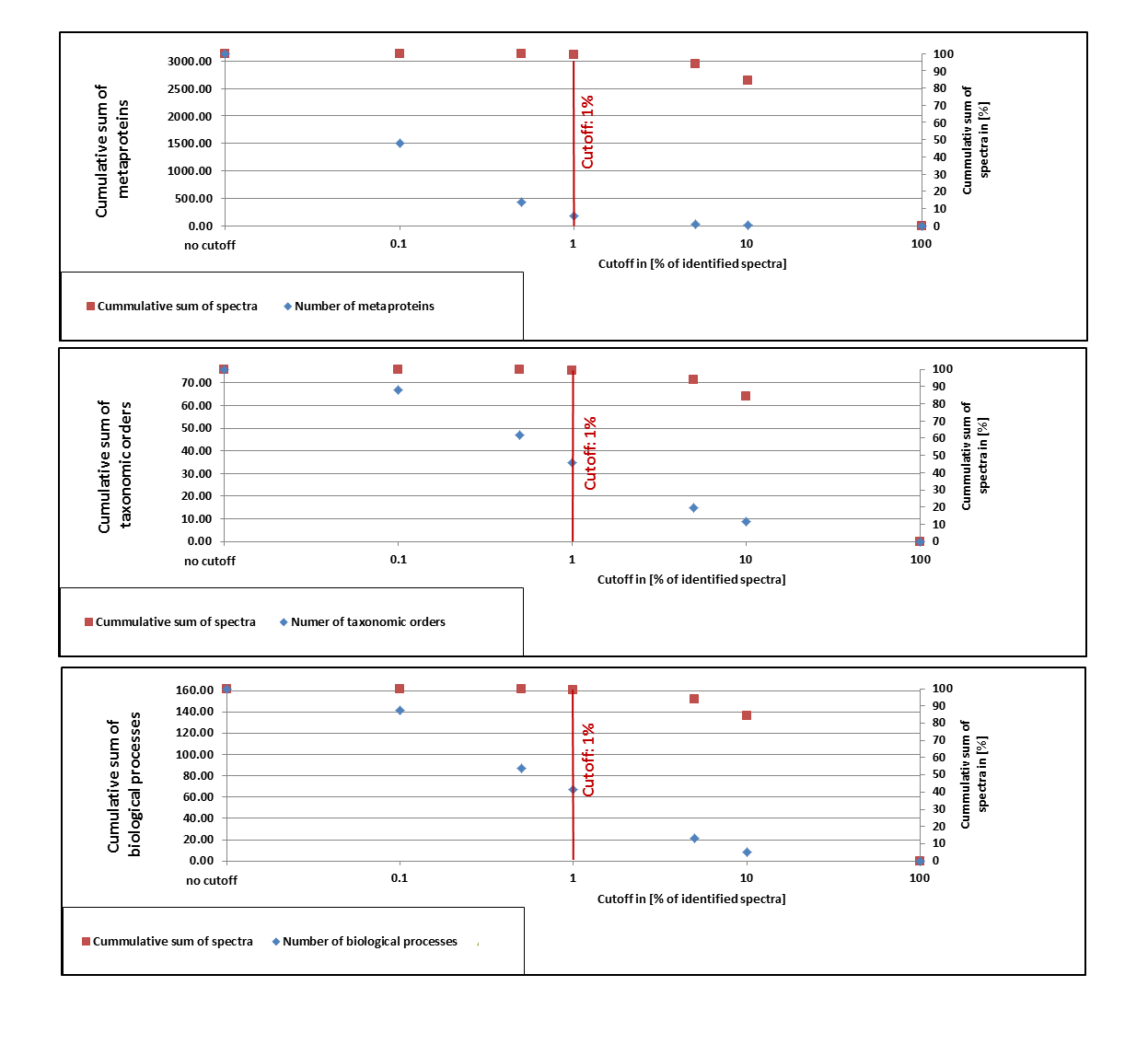

Supplement: Supplementary file 4 — 10.1186/s13068-016-0572-4 Application of different cut-offs for metaprotein, taxonomic order and biological process matrices. In addition the average cumulative sum of identified spectra is shown for different cut-offs. [file 13068_2016_572_MOESM4_ESM.png]

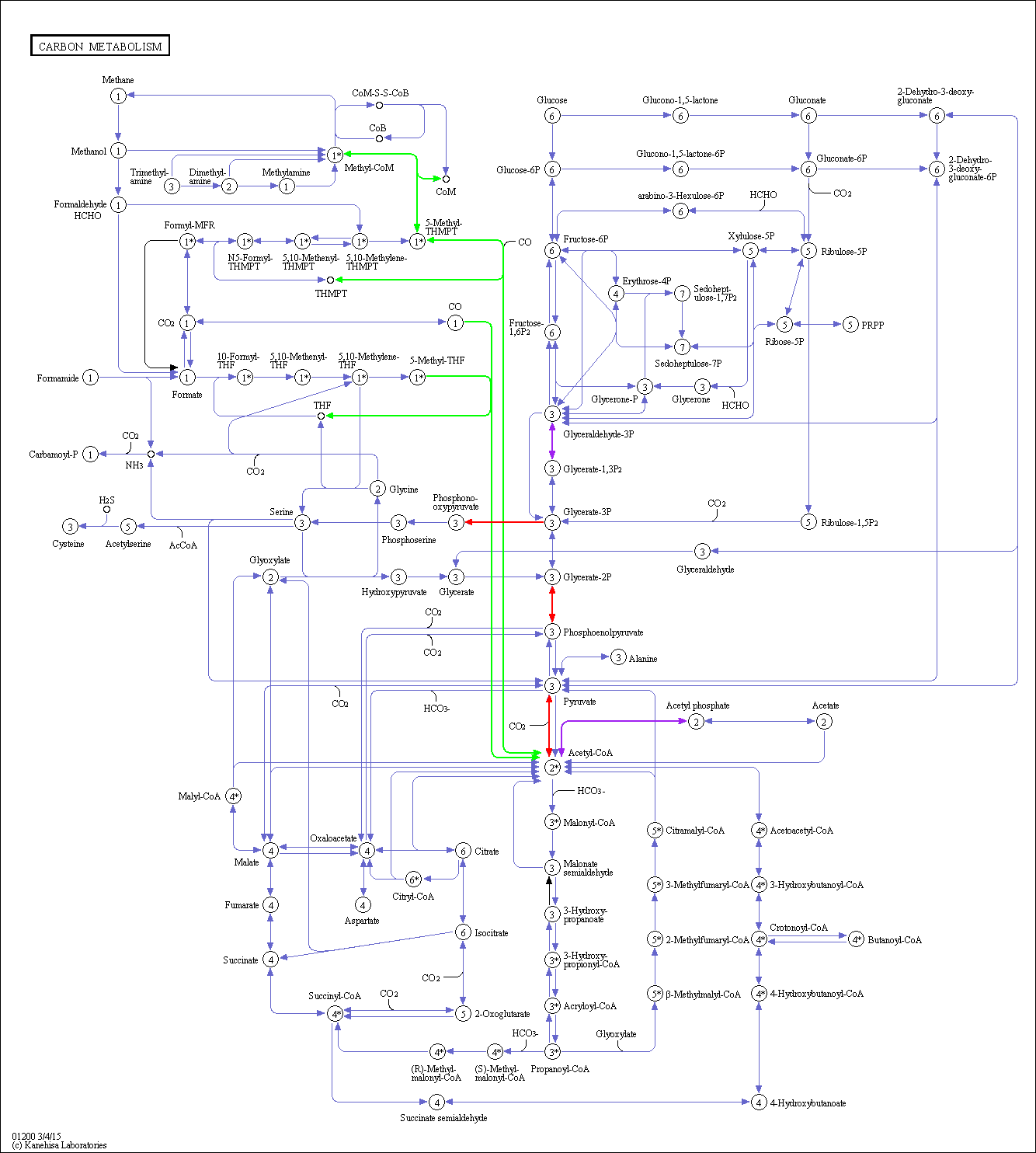

Supplement: Supplementary file 10 — 10.1186/s13068-016-0572-4 Carbon metabolism at high temperatures. Assignment of identified microbial proteins to the KEGG map 1200 (carbon metabolism) positively correlated with high temperature (green: Archaea, red: Bacteria, purple: Archaea or Bacteria). [file 13068_2016_572_MOESM10_ESM.png]

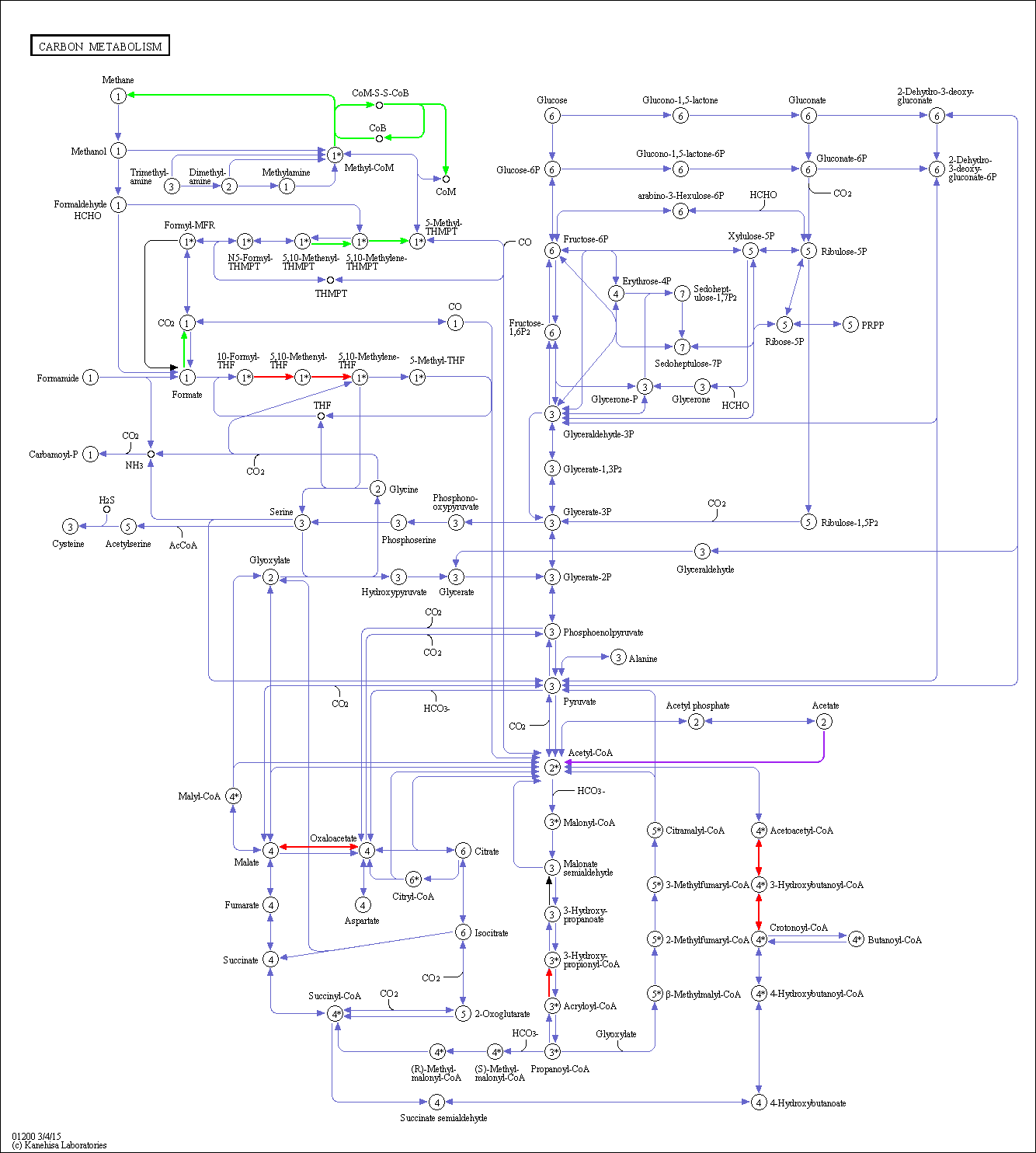

Supplement: Supplementary file 11 — 10.1186/s13068-016-0572-4 Carbon metabolism at low temperatures. Assignment of identified microbial proteins to the KEGG map 1200 (carbon metabolism) negatively correlated with high temperature (green: Archaea, red: Bacteria, purple: Archaea or Bacteria). [file 13068_2016_572_MOESM11_ESM.png]

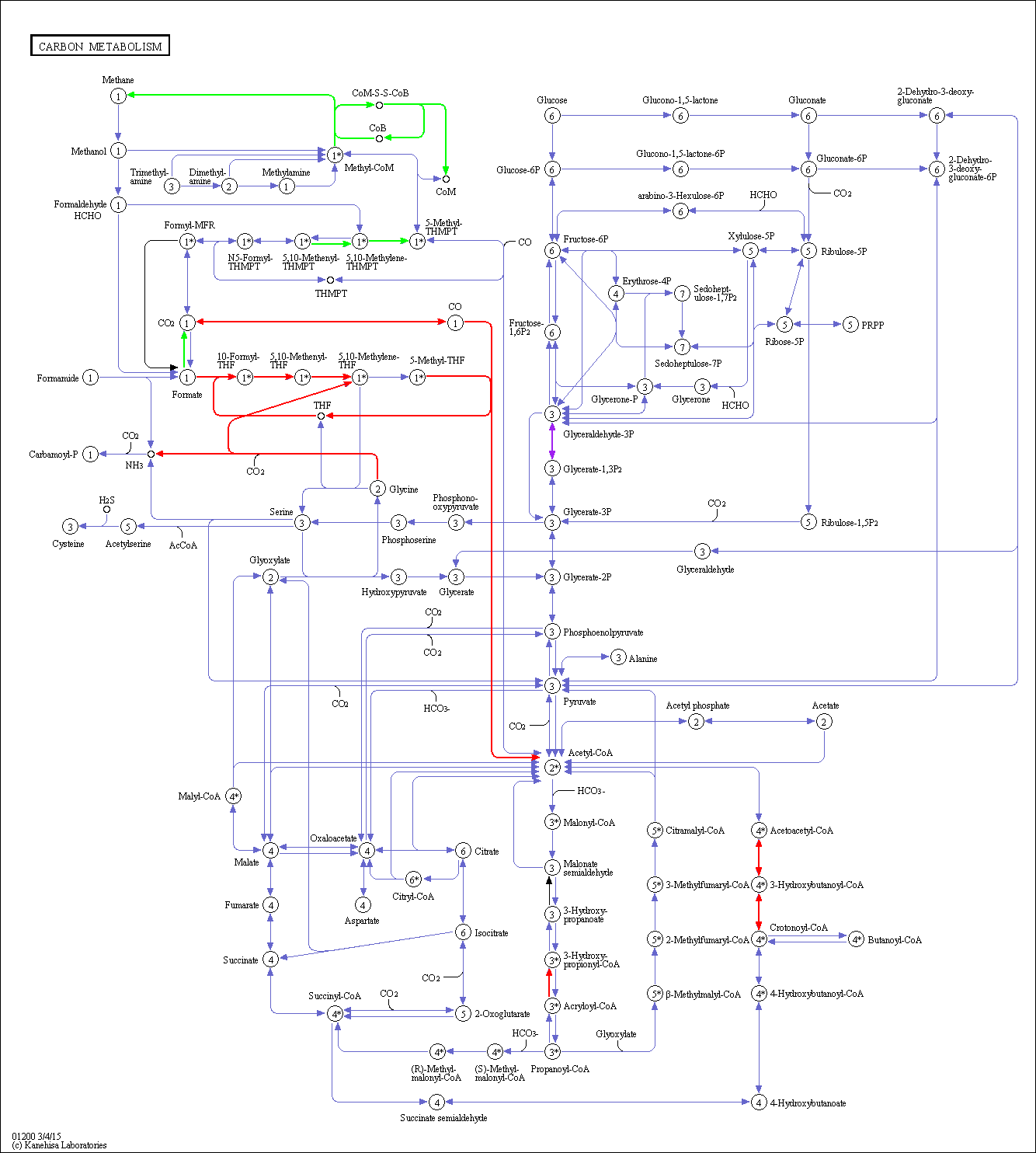

Supplement: Supplementary file 12 — 10.1186/s13068-016-0572-4 Carbon metabolism at high TAN. Assignment of identified microbial proteins to the KEGG map 1200 (carbon metabolism) positively correlated with high TAN (green: Archaea, red: Bacteria, purple: Archaea or Bacteria). [file 13068_2016_572_MOESM12_ESM.png]

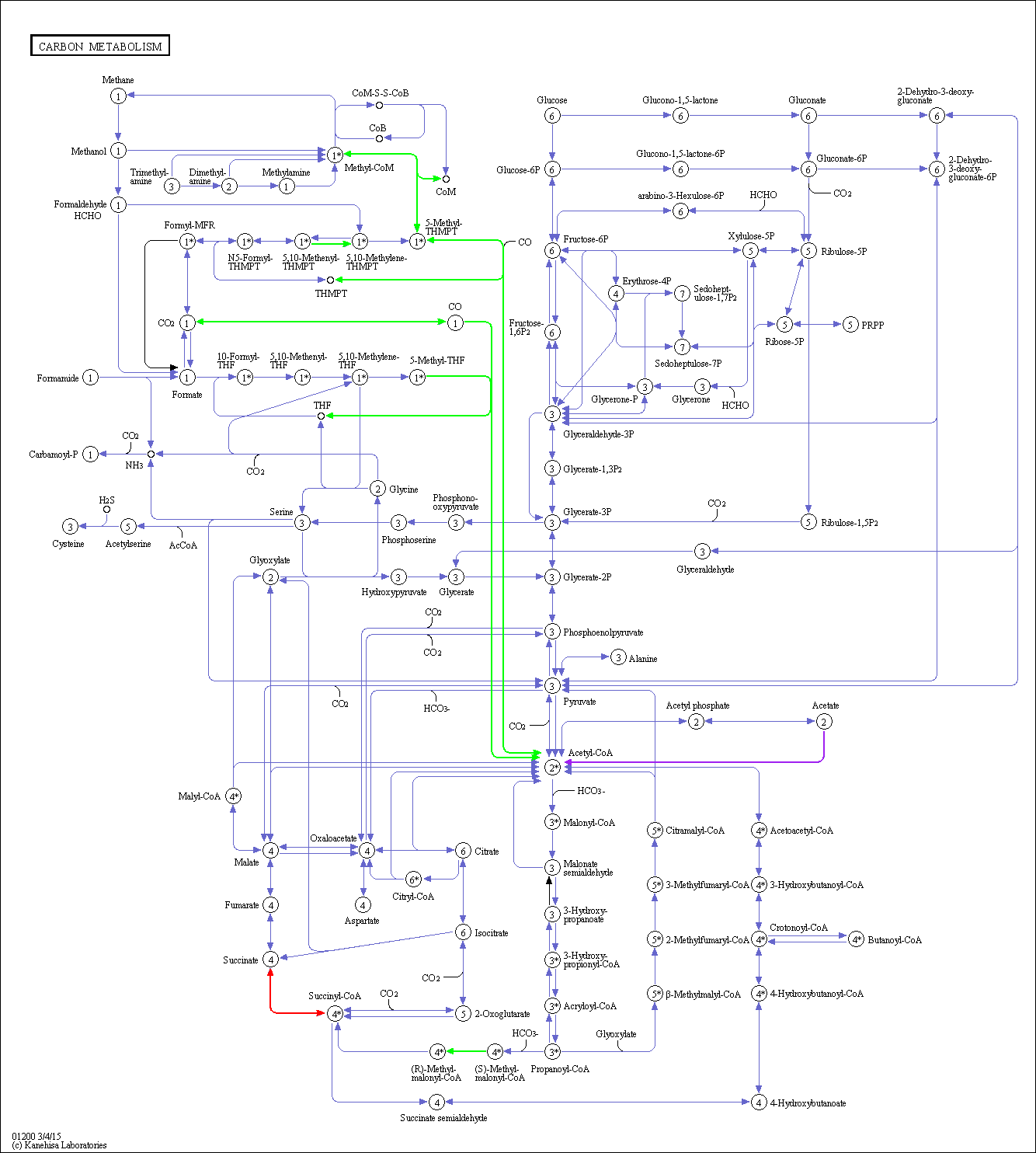

Supplement: Supplementary file 13 — 10.1186/s13068-016-0572-4 Carbon metabolism at low TAN. Assignment of identified microbial proteins to the KEGG map 1200 (carbon metabolism) negatively correlated with high TAN (green: Archaea, red: Bacteria, purple: Archaea or Bacteria). [file 13068_2016_572_MOESM13_ESM.png]

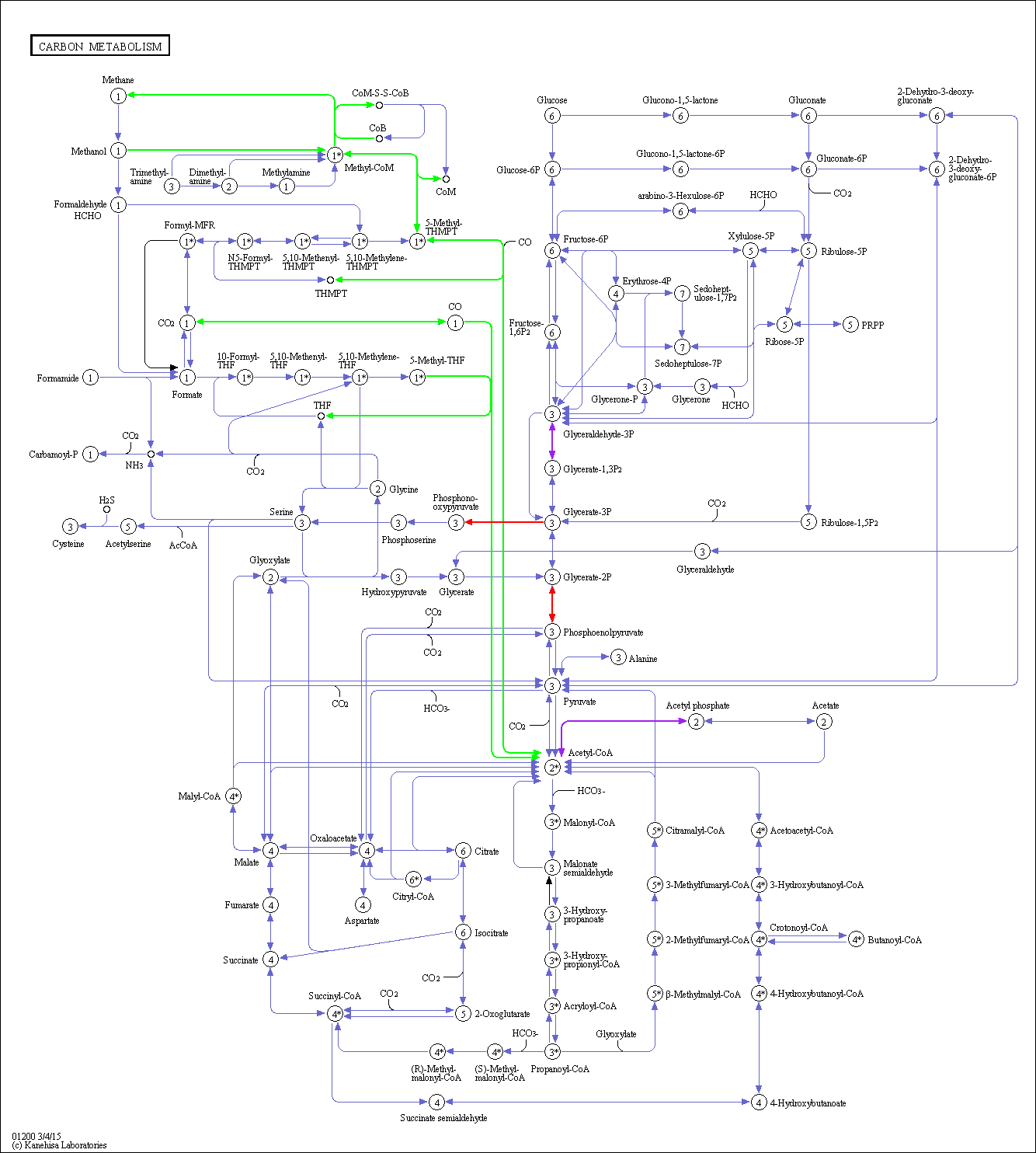

Supplement: Supplementary file 14 — 10.1186/s13068-016-0572-4 Carbon metabolism at high OLR. Assignment of identified microbial proteins to the KEGG map 1200 (carbon metabolism) positively correlated with high OLR (green: Archaea, red: Bacteria, purple: Archaea or Bacteria). [file 13068_2016_572_MOESM14_ESM.png]

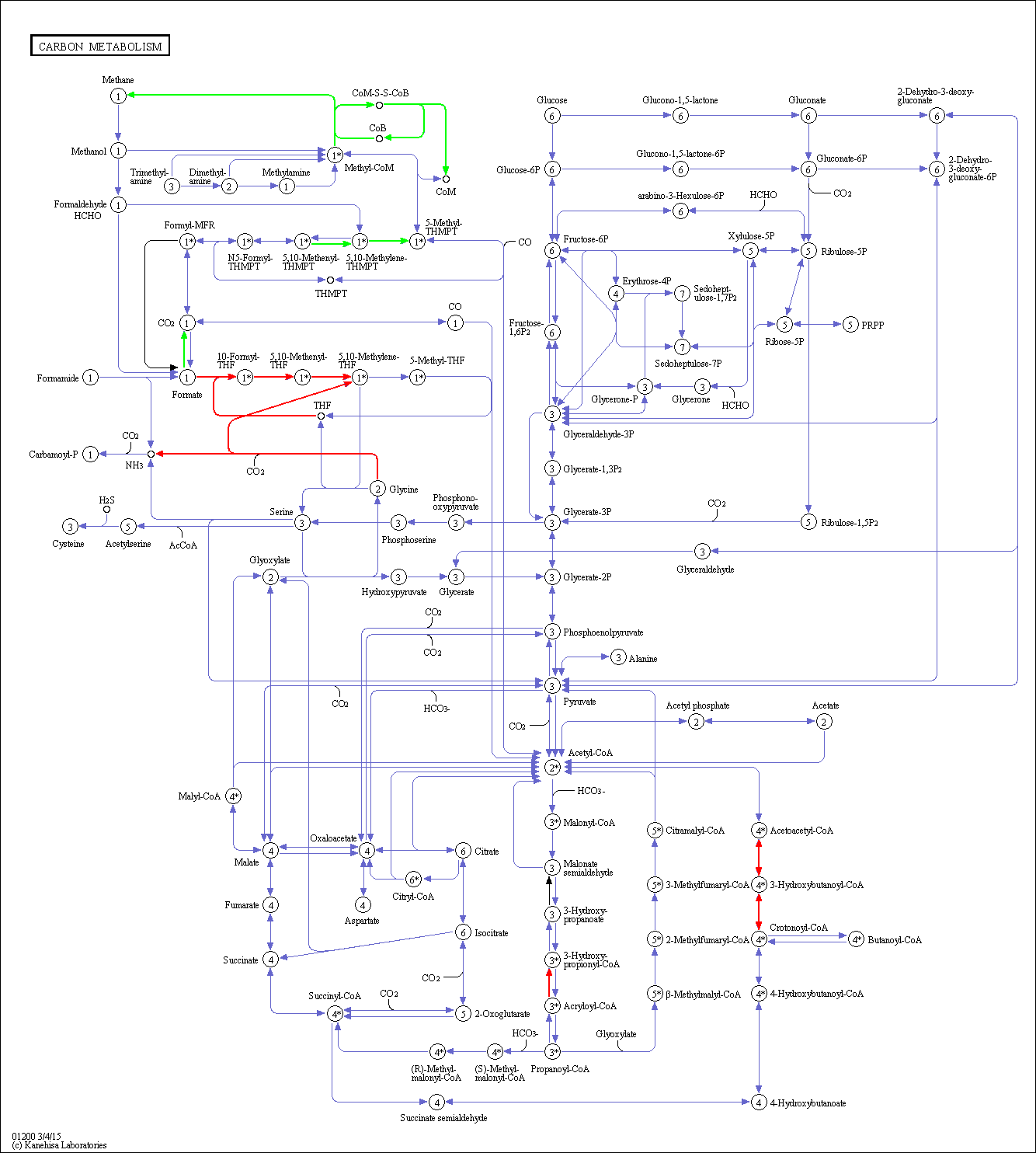

Supplement: Supplementary file 15 — 10.1186/s13068-016-0572-4 Carbon metabolism at low OLR. Assignment of identified microbial proteins to the KEGG map 1200 (carbon metabolism) negatively correlated with high OLR (green: Archaea, red: Bacteria, purple: Archaea or Bacteria). [file 13068_2016_572_MOESM15_ESM.png]

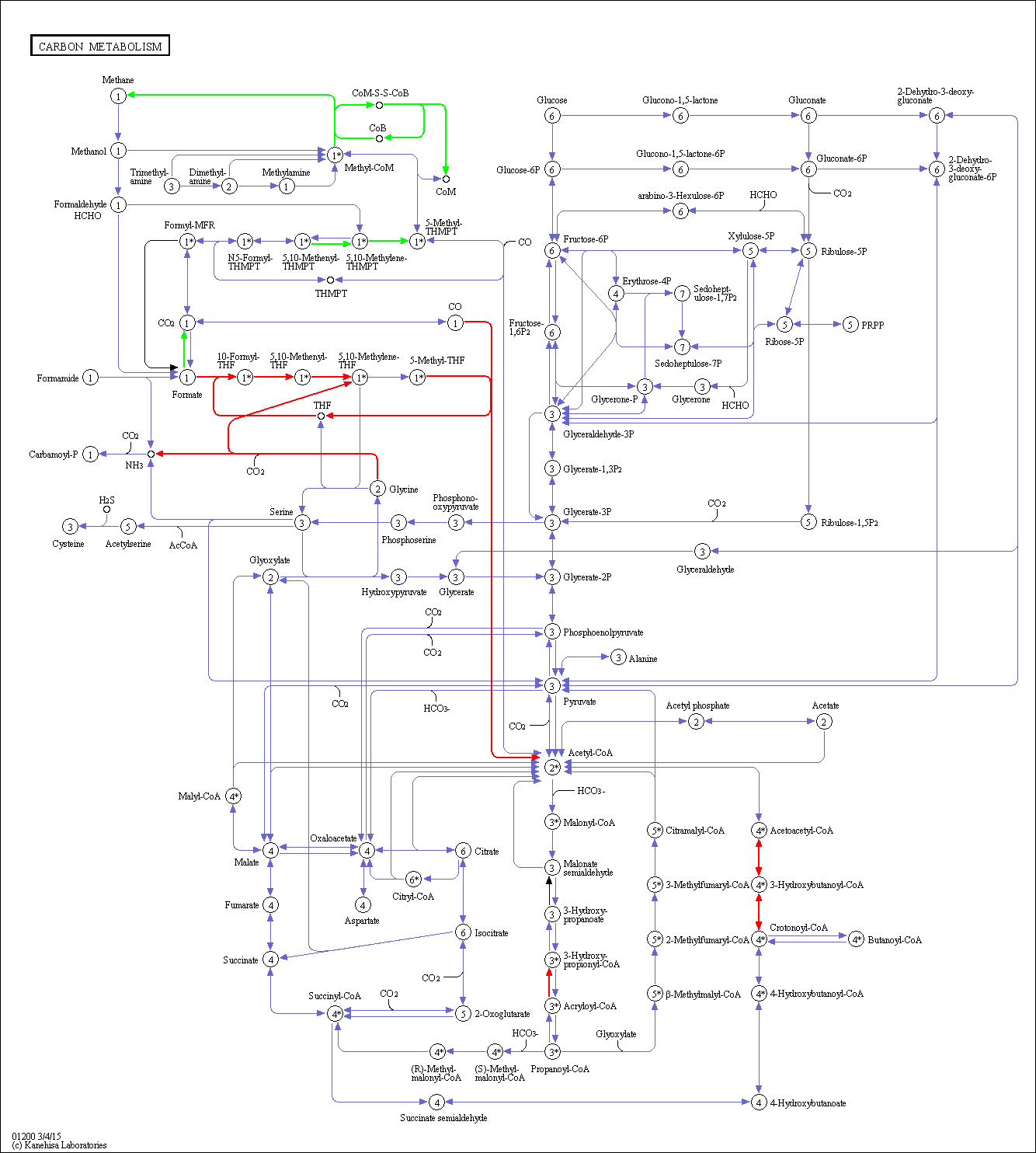

Supplement: Supplementary file 16 — 10.1186/s13068-016-0572-4 Carbon metabolism at high SRT. Assignment of identified microbial proteins to the KEGG map 1200 (carbon metabolism positively correlated with high SRT (green: Archaea, red: Bacteria, purple: Archaea or Bacteria). [file 13068_2016_572_MOESM16_ESM.png]

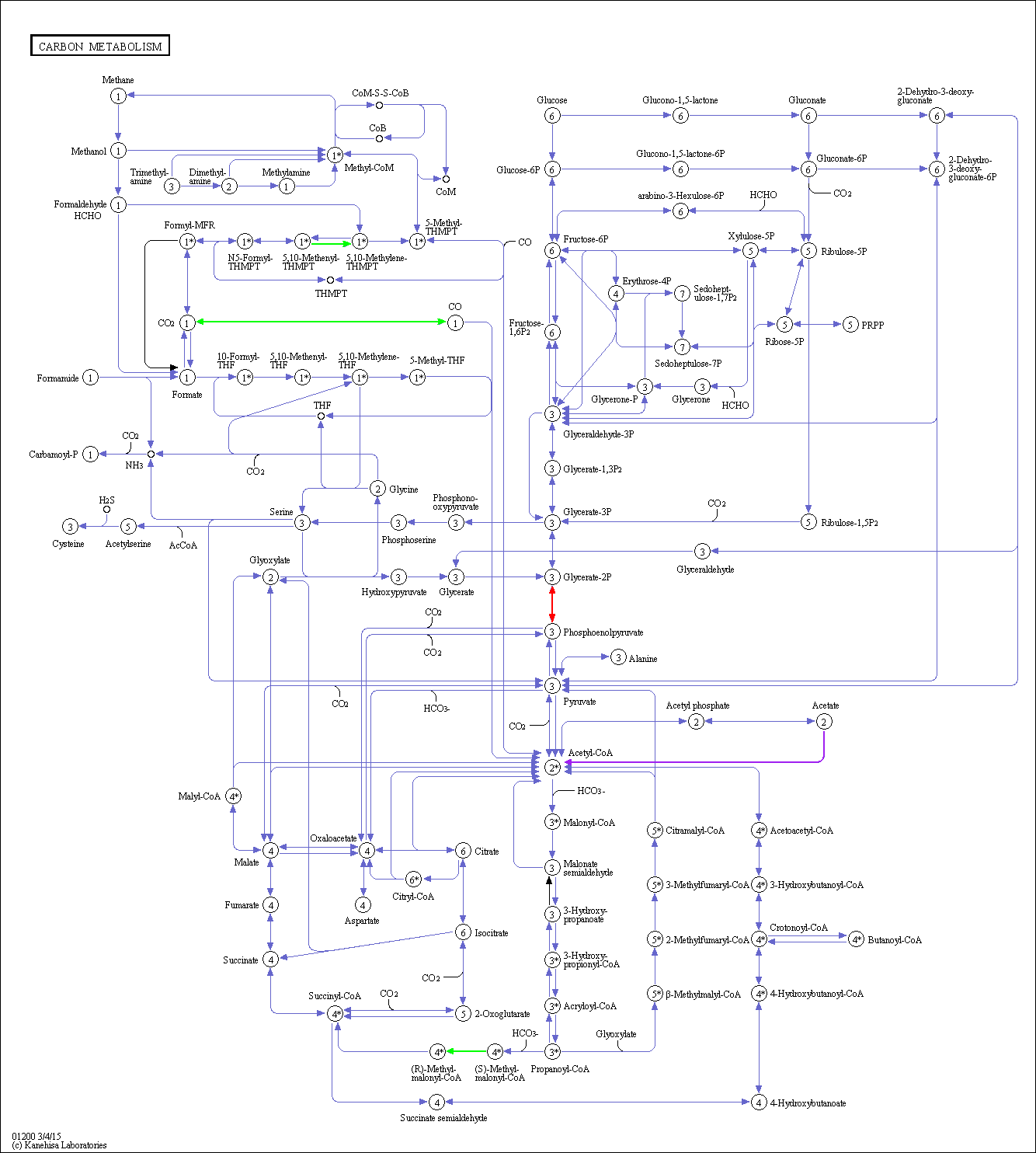

Supplement: Supplementary file 17 — 10.1186/s13068-016-0572-4 Carbon metabolism at low SRT. Assignment of identified microbial proteins to the KEGG map 1200 (carbon metabolism) negatively correlated with high SRT (green: Archaea, red: Bacteria, purple: Archaea or Bacteria). [file 13068_2016_572_MOESM17_ESM.png]

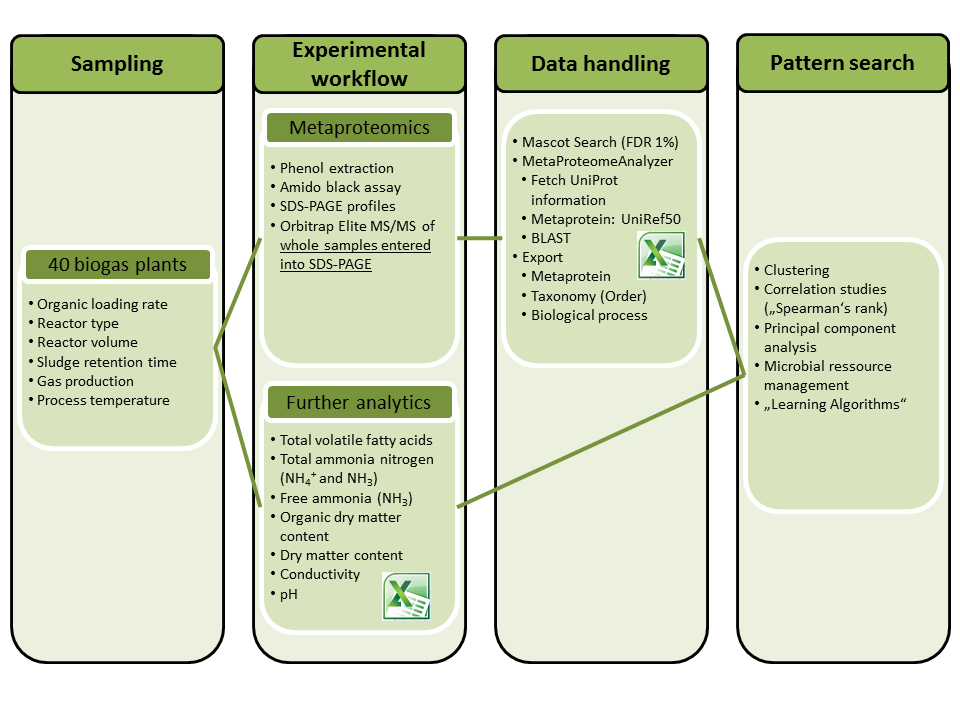

Supplement: Supplementary file 19 — 10.1186/s13068-016-0572-4 Workflow. [file 13068_2016_572_MOESM19_ESM.png]
