# Supplementary material for: Proteotyping of biogas plant microbiomes separates biogas plants according to process temperature and reactor type
Source: Biotechnol Biofuels. 2016 Jul 26;9:155. doi: 10.1186/s13068-016-0572-4 (PMC4960849; doi:10.1186/s13068-016-0572-4)
Supplement: Supplementary file 5 — 10.1186/s13068-016-0572-4 Detailed description of protein identifications and list of all metaproteins, orders and biological processes. [file 13068_2016_572_MOESM5_ESM.docx]

Additional File 5: Note 1

Protein identification from different biogas plants – a detailed consideration

**Preliminary evaluation of extracted proteins**

Evaluation of protein extraction by sodium dodecyl sulfate polyacrylamide gel electrophoresis (SDS-PAGE) revealed the presence of proteins covering the whole molecular weight range [Additional file 2: Figure S1]. Several dominating bands were observed in nearly all lanes. Two lanes were smeared (Gent 15 and Gent 27) and one lane showed only a weak intensity (Gent 37). The low quality of these three lanes might be caused by humic substances interfering with protein separation and quantification [1]. Some bands were present in nearly all lanes, whereas others were unique for certain BGPs, indicating both common and specific functions of the microbial communities [2].

**Protein identification**

Reasonable experimental effort, in particular regarding the measurement time for MS, is one precondition for high throughput metaproteomic analysis of samples from BGPs. Accordingly, samples were not pre-fractionated but measured in triplicates to meet requirements with respect to quantification in state-of-the-art proteome studies [1]. Approximately 40,000 spectra were measured per single run of which about 500-1,000 spectra were identified searching with Mascot and a score threshold of 40 [3] against UniProtKB/Swiss-Prot [Additional file 3: Figure S2]. The number of proteins identified with the sensitive Orbitrap MS exceeded the numbers previously detected after pre-fractionation of proteins by SDS-PAGE and subsequent use of standard ion trap MS of tryptic peptides (2D-approach) [4]. However, it failed to reach the sensitivity of a more extensive 3D-approach, which included liquid isoelectric focusing before performing a SDS-PAGE [4]. To improve protein identification in this study, several metagenomes [5-7] were added to UniProtKB/Swiss-Prot database. In consequence, the number of identified spectra [Additional file 3: Figure S2] raised from 500 to 4,000 for the agricultural BGPs. However, the number of additionally identified spectra by the metagenomes was significantly lower for BGPs operating with industrial waste or sewage sludge as well as for UASB reactors. This is most likely due to the fact that the metagenomes sequenced from agricultural CSTR-BGPs [5-7] do not represent the genetic information of these microbial communities. According to state of the art requirements for proteomics, a false discovery rate (FDR) of 1% [8] was applied for all following steps of the data evaluation. For most BGPs, a similar number of spectra was identified. For BGPs poorly represented by the added metagenomes, the total number of identified spectra was even lower than the number of spectra identified against UniProtKB/Swiss-Prot alone.

Identification of spectra resulted in about 90% of redundant proteins. Therefore, metaproteome results were loaded into the MPA software and redundant proteins were consolidated by grouping the corresponding identifications to metaproteins based on their affiliation to UniRef50 clusters. In a next step, the data of all BGPs were exported from MPA and combined to fusion matrices containing 3138 identified metaproteins [Additional file 6: Table S1], 75 taxonomic orders [Additional file 6: Table S2] and 162 biological processes [Additional file 6: Table S3].

Untargeted LC-MS/MS-based proteomic approaches using data-dependent acquisition for MS/MS suffer from variations of low abundant protein identifications, due to small differences between the measurements [9]. Therefore, technical triplicates were measured and mean abundances of the number of identified spectra were calculated in this study. Non-significant metaproteins, taxonomies and biological processes were removed by applying different cut-offs for abundances [Additional file 4: Figure S3]. Applying a cut-off of 1% minimal abundance of identified spectra in at least one BGP still covered over 99% of the total identified spectra in all BGPs, and resulted in a reduction of the data to 187 metaproteins (6% of 3138 metaproteins), 35 taxonomic orders (46% of 75 taxonomic orders) and 65 biological processes (40% of 162 biological processes). The high coverage of corresponding spectra showed that some metaproteins and, to a lesser extent, also functions and taxonomies were highly abundant.

One major objective of this study was to identify correlations between process parameters and the metaproteins, taxonomic orders as well as biological processes. Therefore, a correlation matrix of these data based on the abundance of their spectra (Spearman’s rank correlation, p-value: 0.05) was created to explore possible interactions. [Additional file 7: Table S1]. A correlation analysis of all these 350 parameters revealed more than 14,000 correlations. Due to this huge number of correlations, its number was decreased by a decrease of the p-value to 0.01 and an increase of the cut-off for the minimal spectral abundance to 5%.

This resulted in 744 correlations for the remaining 77 parameters [Additional file 7: Table S2].

**References**

1. Heyer R, Kohrs F, Reichl U, Benndorf D. Metaproteomics of complex microbial communities in biogas plants. Microb Biotechnol. 2015;8(5):749-63. doi:10.1111/1751-7915.12276.

2. Heyer R, Kohrs F, Benndorf D, Rapp E, Kausmann R, Heiermann M et al. Metaproteome analysis of the microbial communities in agricultural biogas plants. New Biotechnology. 2013;30(6):614-22. doi:10.1016/j.nbt.2013.01.002.

3. Perkins DN, Pappin DJC, Creasy DM, Cottrell JS. Probability-based protein identification by searching sequence databases using mass spectrometry data. Electrophoresis. 1999;20(18):3551-67. doi:Doi 10.1002/(Sici)1522-2683(19991201)20:18<3551::Aid-Elps3551>3.0.Co;2-2.

4. Kohrs F, Heyer R, Magnussen A, Benndorf D, Muth T, Behne A et al. Sample prefractionation with liquid isoelectric focusing enables in depth microbial metaproteome analysis of mesophilic and thermophilic biogas plants. Anaerobe. 2014;29:59-67. doi:10.1016/j.anaerobe.2013.11.009.

5. Hanreich A, Schimpf U, Zakrzewski M, Schlüter A, Benndorf D, Heyer R et al. Metagenome and metaproteome analyses of microbial communities in mesophilic biogas-producing anaerobic batch fermentations indicate concerted plant carbohydrate degradation. Systematic and Applied Microbiology. 2013;36(5):330-8. doi:10.1016/j.syapm.2013.03.006.

6. Rademacher A, Zakrzewski M, Schlüter A, Schonberg M, Szczepanowski R, Goesmann A et al. Characterization of microbial biofilms in a thermophilic biogas system by high-throughput metagenome sequencing. Fems Microbiol Ecol. 2012;79(3):785-99. doi:10.1111/j.1574-6941.2011.01265.x.

7. Schlüter A, Bekel T, Diaz NN, Dondrup M, Eichenlaub R, Gartemann KH et al. The metagenome of a biogas-producing microbial community of a production-scale biogas plant fermenter analysed by the 454-pyrosequencing technology. Journal of Biotechnology. 2008;136(1-2):77-90. doi:10.1016/j.jbiotec.2008.05.008.

8. Barnouin K. Guidelines for experimental design and data analysis of proteomic mass spectrometry-based experiments. Amino Acids. 2011;40(2):259-60. doi:10.1007/s00726-010-0750-9.

9. Tabb DL, Vega-Montoto L, Rudnick PA, Variyath AM, Ham AJL, Bunk DM et al. Repeatability and Reproducibility in Proteomic Identifications by Liquid Chromatography-Tandem Mass Spectrometry. Journal of Proteome Research. 2010;9(2):761-76. doi:10.1021/pr9006365.
