# Supplementary material for: Proteotyping of biogas plant microbiomes separates biogas plants according to process temperature and reactor type
Source: Biotechnol Biofuels. 2016 Jul 26;9:155. doi: 10.1186/s13068-016-0572-4 (PMC4960849; doi:10.1186/s13068-016-0572-4)

Loadings

PCA based on taxonomic orders (25% variance)

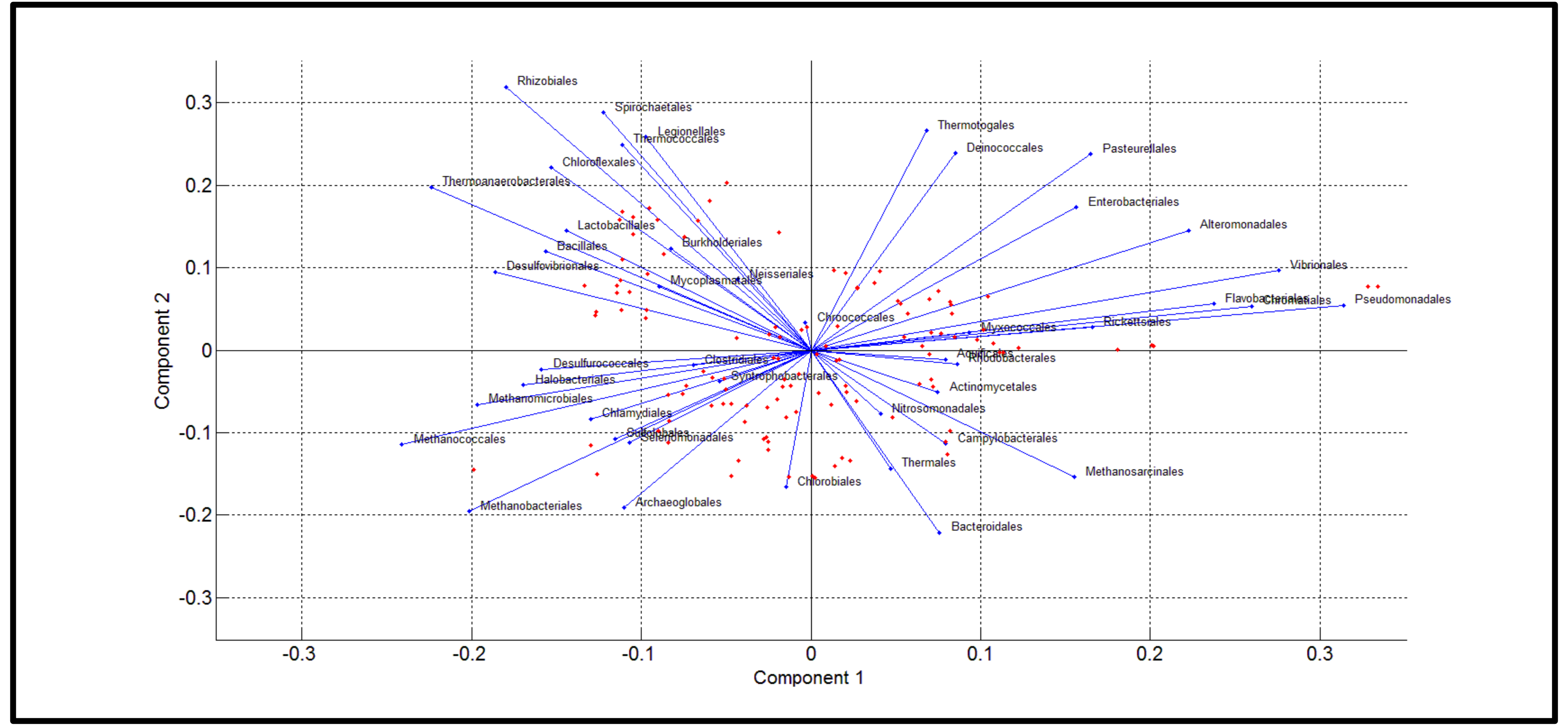

PCA based on biological processes (33% variance)

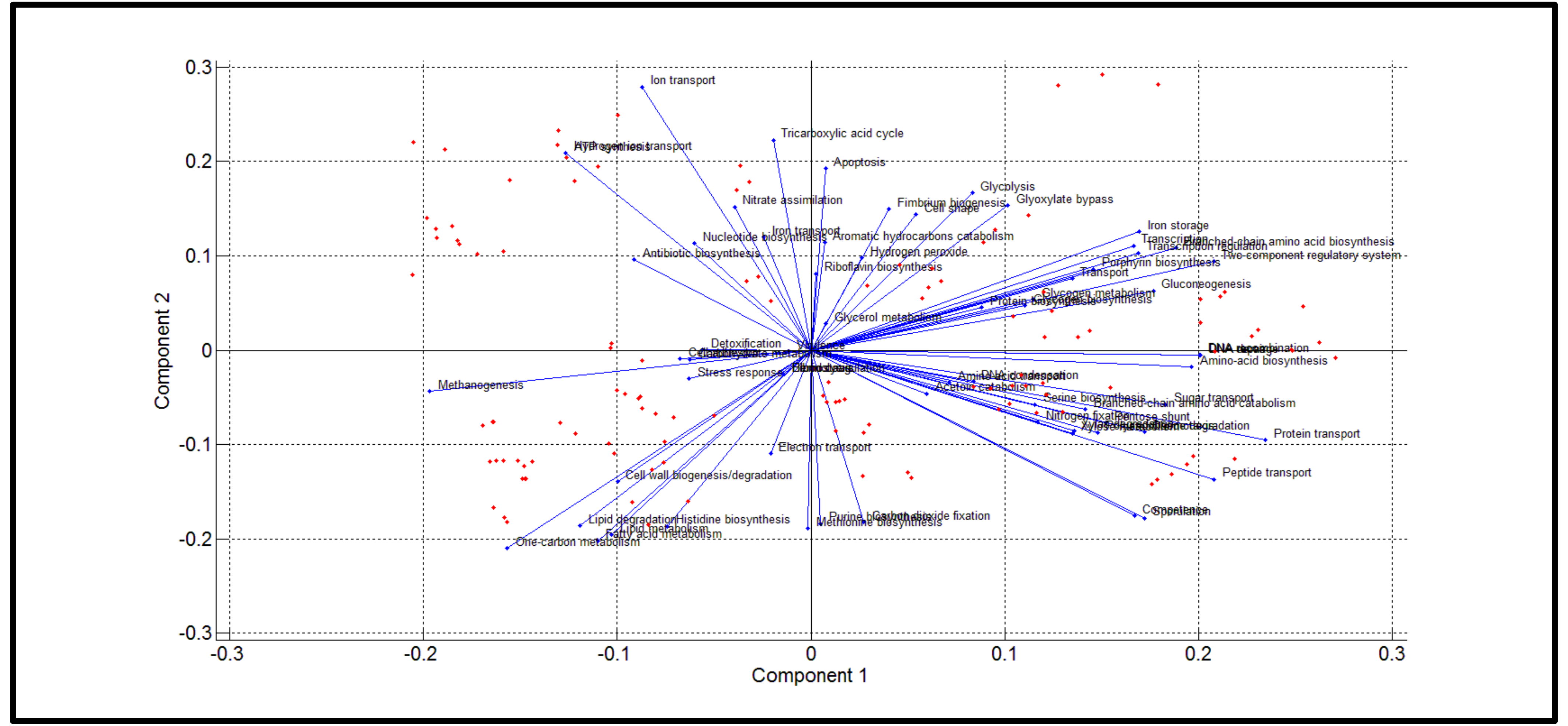

PCA based on metaproteins (28% variance)

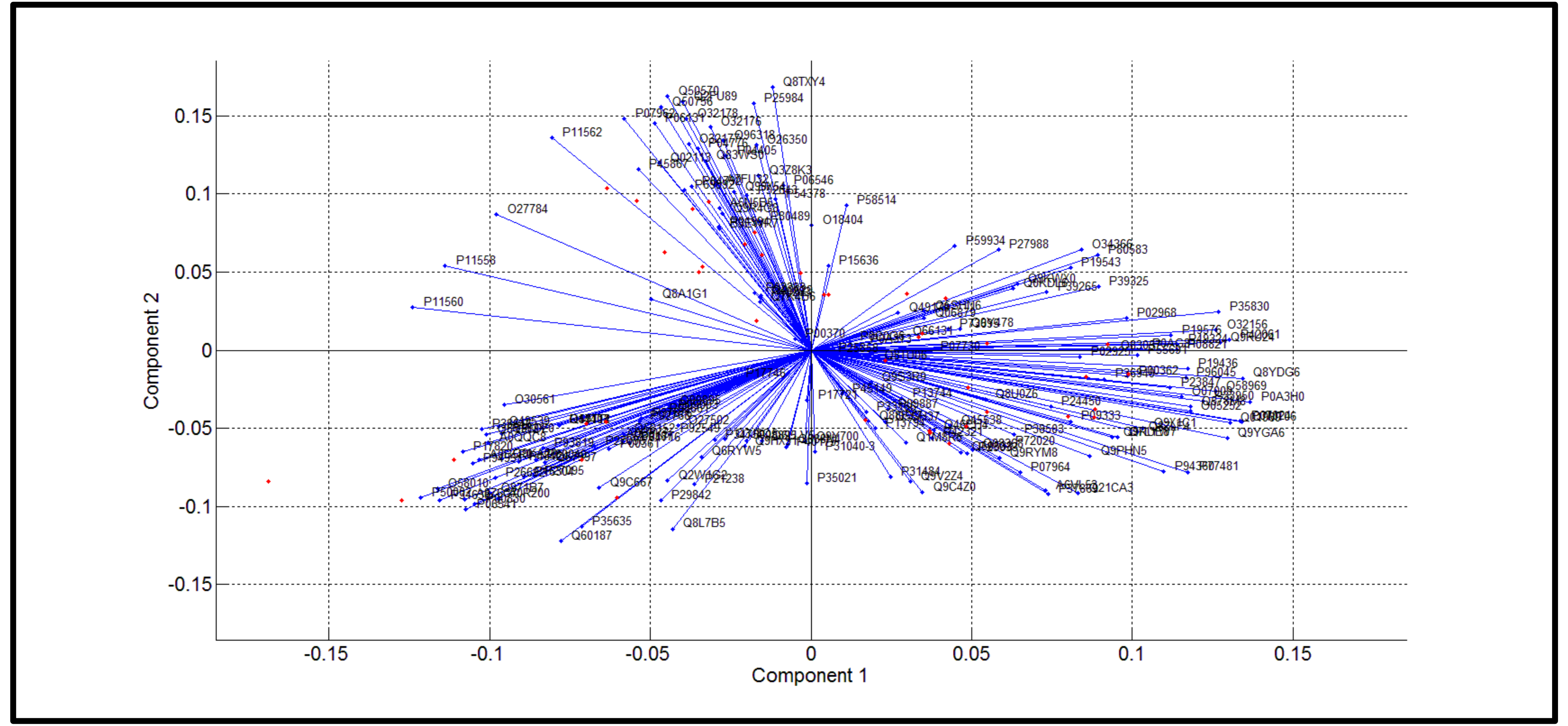

Supplement: Supplementary file 8 — 10.1186/s13068-016-0572-4 Loading Plots for the PCAs shown in Fig. 2. [file 13068_2016_572_MOESM8_ESM.pdf]
