# Supplementary material for: Proteotyping of biogas plant microbiomes separates biogas plants according to process temperature and reactor type
Source: Biotechnol Biofuels. 2016 Jul 26;9:155. doi: 10.1186/s13068-016-0572-4 (PMC4960849; doi:10.1186/s13068-016-0572-4)
Supplement: Supplementary file 22 — 10.1186/s13068-016-0572-4 Summary. [file 13068_2016_572_MOESM22_ESM.pdf]

Main class for investigation of metaproteome data

Name: Start.m Author: Robert Heyer Date : 01.06.2015 Version: 03

Contents

- Clean everything
- Load classifications
- Taxonomy Data
- Process data
- Biological function Ontology
- Metaprotein Data
- Perform mixed correlations necessary that both matrixes has the same number of entries
- FINISH

Clean everything

```
clc; clear all; close all;
```

Load classifications

C.reactor, C.pH, C.tan, C.power,C.biogas, C.vfa, C.olr, C.srt, C.temp , C.fa, C.biogasPerOlr

```
C  = LoadClassification();
```

Taxonomy Data

1. Read taxonomy data

```
[tax_matrix_original, TaxMatrixPlusDescription] = ReadTaxFiles();
% Sort by taxonomy profile

% 2. Remove root entry
for root = 1: size(tax_matrix_original, 1)
   if (strcmp(tax_matrix_original(root,1),  'root'))
       tax_matrix_original(root,:) = [];
       break
   end
end
clearvars root;

% 3. Contracting the length of the sample names
for entry = 2: size(tax_matrix_original, 2)
    str = cell2mat(tax_matrix_original(1, entry));
    tax_matrix_original(1, entry) = {regexprep(str,'RH_Tax_Order_UniRef50_allSpecies_Gent',''
end
clearvars str, entry;

% Remove "Unknown"-entry
for unknown = 1: size(tax_matrix_original, 1)
    if (strcmp(tax_matrix_original(unknown,1),  'unknown'))
        tax_matrix_original(unknown,:) = [];
        break;       % 'UNKNOWN" is only once in the dataset
    end
end
clearvars unknown;
```

```
end
clearvars unknown;

% 4. Filtering and normalizing of the "tax_matrix_original" with at least
% one entry, were the taxonomy represents at least 1% of the spectra
[tax_matrix_filtered, specAverageBgp] = FilterAndNormalize(tax_matrix_original, 0.01);
% 4b. Sort after the taxTree
tax_matrix_filtered_desc = cell(size(tax_matrix_filtered,1), size(tax_matrix_filtered,2)+5);
tax_matrix_filtered_desc(:,1) = tax_matrix_filtered(:,1);
tax_matrix_filtered_desc(:,7:end) = tax_matrix_filtered(:,2:end);
%  Add description to the taxonomy matrix
for tax = 1: size(tax_matrix_filtered,1)
   for entries = 1 : size(TaxMatrixPlusDescription, 1)
       if (isequal(tax_matrix_filtered(tax,1), TaxMatrixPlusDescription(entries,1)) )
           tax_matrix_filtered_desc(tax,2) = TaxMatrixPlusDescription(entries,2); % Superking
           tax_matrix_filtered_desc(tax,3) = TaxMatrixPlusDescription(entries,3); % Kingdom
           tax_matrix_filtered_desc(tax,4) = TaxMatrixPlusDescription(entries,4); % Phylum
           tax_matrix_filtered_desc(tax,5) = TaxMatrixPlusDescription(entries,5); % Class
           tax_matrix_filtered_desc(tax,6) = TaxMatrixPlusDescription(entries,6); % Order
       end
   end
end
clearvars tax entries;

[tax_average_matrix_filtered]= AverageMatrix(tax_matrix_filtered);
% 5. Create the correlation matrix
[tax_correlationMatrix2, pValues] = CalculateCorrelation_V2(tax_average_matrix_filtered, tax_
%  Add description to the correlation matrix
tax_correlationMatrix_desc = cell(size(tax_correlationMatrix2,1), size(tax_correlationMatrix2
tax_correlationMatrix_desc(:,1:6) = tax_matrix_filtered_desc(:,1:6);
tax_correlationMatrix_desc(:,7:end) = tax_correlationMatrix2(:,2:end);

% 6. Perform PCA
[coeff,Scores,latent] =PerformPCA(tax_matrix_filtered, tax_matrix_filtered(1, 2:end),'Tax');
PerformPCA(tax_average_matrix_filtered, tax_average_matrix_filtered(1, 2:end),'Tax_Norm');
```

```
IMPORTED: RH_Tax_Order_UniRef50_allSpecies_Gent01a
IMPORTED: RH_Tax_Order_UniRef50_allSpecies_Gent01b
IMPORTED: RH_Tax_Order_UniRef50_allSpecies_Gent01c
IMPORTED: RH_Tax_Order_UniRef50_allSpecies_Gent02a
IMPORTED: RH_Tax_Order_UniRef50_allSpecies_Gent02b
IMPORTED: RH_Tax_Order_UniRef50_allSpecies_Gent02c
IMPORTED: RH_Tax_Order_UniRef50_allSpecies_Gent03a
IMPORTED: RH_Tax_Order_UniRef50_allSpecies_Gent03b
IMPORTED: RH_Tax_Order_UniRef50_allSpecies_Gent03c
IMPORTED: RH_Tax_Order_UniRef50_allSpecies_Gent04a
IMPORTED: RH_Tax_Order_UniRef50_allSpecies_Gent04b
IMPORTED: RH_Tax_Order_UniRef50_allSpecies_Gent04c
IMPORTED: RH_Tax_Order_UniRef50_allSpecies_Gent05a
IMPORTED: RH_Tax_Order_UniRef50_allSpecies_Gent05b
IMPORTED: RH_Tax_Order_UniRef50_allSpecies_Gent05c
IMPORTED: RH_Tax_Order_UniRef50_allSpecies_Gent06a
IMPORTED: RH_Tax_Order_UniRef50_allSpecies_Gent06b
IMPORTED: RH_Tax_Order_UniRef50_allSpecies_Gent06c
IMPORTED: RH_Tax_Order_UniRef50_allSpecies_Gent07a
IMPORTED: RH_Tax_Order_UniRef50_allSpecies_Gent07b
IMPORTED: RH_Tax_Order_UniRef50_allSpecies_Gent07c
IMPORTED: RH_Tax_Order_UniRef50_allSpecies_Gent08a
IMPORTED: RH_Tax_Order_UniRef50_allSpecies_Gent08b
IMPORTED: RH_Tax_Order_UniRef50_allSpecies_Gent08c
IMPORTED: RH_Tax_Order_UniRef50_allSpecies_Gent09a
IMPORTED: RH_Tax_Order_UniRef50_allSpecies_Gent09b
[illegible]
```

```
IMPORTED: RH_Tax_Order_UniRef50_allSpecies_Gent08c
IMPORTED: RH_Tax_Order_UniRef50_allSpecies_Gent09a
IMPORTED: RH_Tax_Order_UniRef50_allSpecies_Gent09b
IMPORTED: RH_Tax_Order_UniRef50_allSpecies_Gent09c
IMPORTED: RH_Tax_Order_UniRef50_allSpecies_Gent10a
IMPORTED: RH_Tax_Order_UniRef50_allSpecies_Gent10b
IMPORTED: RH_Tax_Order_UniRef50_allSpecies_Gent10c
IMPORTED: RH_Tax_Order_UniRef50_allSpecies_Gent11a
IMPORTED: RH_Tax_Order_UniRef50_allSpecies_Gent11b
IMPORTED: RH_Tax_Order_UniRef50_allSpecies_Gent11c
IMPORTED: RH_Tax_Order_UniRef50_allSpecies_Gent12a
IMPORTED: RH_Tax_Order_UniRef50_allSpecies_Gent12b
IMPORTED: RH_Tax_Order_UniRef50_allSpecies_Gent12c
IMPORTED: RH_Tax_Order_UniRef50_allSpecies_Gent13a
IMPORTED: RH_Tax_Order_UniRef50_allSpecies_Gent13b
IMPORTED: RH_Tax_Order_UniRef50_allSpecies_Gent13c
IMPORTED: RH_Tax_Order_UniRef50_allSpecies_Gent14a
IMPORTED: RH_Tax_Order_UniRef50_allSpecies_Gent14b
IMPORTED: RH_Tax_Order_UniRef50_allSpecies_Gent14c
IMPORTED: RH_Tax_Order_UniRef50_allSpecies_Gent15a
IMPORTED: RH_Tax_Order_UniRef50_allSpecies_Gent15b
IMPORTED: RH_Tax_Order_UniRef50_allSpecies_Gent15c
IMPORTED: RH_Tax_Order_UniRef50_allSpecies_Gent16a
IMPORTED: RH_Tax_Order_UniRef50_allSpecies_Gent16b
IMPORTED: RH_Tax_Order_UniRef50_allSpecies_Gent16c
IMPORTED: RH_Tax_Order_UniRef50_allSpecies_Gent17a
IMPORTED: RH_Tax_Order_UniRef50_allSpecies_Gent17b
IMPORTED: RH_Tax_Order_UniRef50_allSpecies_Gent17c
IMPORTED: RH_Tax_Order_UniRef50_allSpecies_Gent18a
IMPORTED: RH_Tax_Order_UniRef50_allSpecies_Gent18b
IMPORTED: RH_Tax_Order_UniRef50_allSpecies_Gent18c
IMPORTED: RH_Tax_Order_UniRef50_allSpecies_Gent19a
IMPORTED: RH_Tax_Order_UniRef50_allSpecies_Gent19b
IMPORTED: RH_Tax_Order_UniRef50_allSpecies_Gent19c
IMPORTED: RH_Tax_Order_UniRef50_allSpecies_Gent20a
IMPORTED: RH_Tax_Order_UniRef50_allSpecies_Gent20b
IMPORTED: RH_Tax_Order_UniRef50_allSpecies_Gent20c
IMPORTED: RH_Tax_Order_UniRef50_allSpecies_Gent21a
IMPORTED: RH_Tax_Order_UniRef50_allSpecies_Gent21c
IMPORTED: RH_Tax_Order_UniRef50_allSpecies_Gent21d
IMPORTED: RH_Tax_Order_UniRef50_allSpecies_Gent22a
IMPORTED: RH_Tax_Order_UniRef50_allSpecies_Gent22b
IMPORTED: RH_Tax_Order_UniRef50_allSpecies_Gent22c
IMPORTED: RH_Tax_Order_UniRef50_allSpecies_Gent23a
IMPORTED: RH_Tax_Order_UniRef50_allSpecies_Gent23b
IMPORTED: RH_Tax_Order_UniRef50_allSpecies_Gent23c
IMPORTED: RH_Tax_Order_UniRef50_allSpecies_Gent24a
IMPORTED: RH_Tax_Order_UniRef50_allSpecies_Gent24b
IMPORTED: RH_Tax_Order_UniRef50_allSpecies_Gent24c
IMPORTED: RH_Tax_Order_UniRef50_allSpecies_Gent25a
IMPORTED: RH_Tax_Order_UniRef50_allSpecies_Gent25b
IMPORTED: RH_Tax_Order_UniRef50_allSpecies_Gent25c
IMPORTED: RH_Tax_Order_UniRef50_allSpecies_Gent26a
IMPORTED: RH_Tax_Order_UniRef50_allSpecies_Gent26b
IMPORTED: RH_Tax_Order_UniRef50_allSpecies_Gent26c
IMPORTED: RH_Tax_Order_UniRef50_allSpecies_Gent27a
IMPORTED: RH_Tax_Order_UniRef50_allSpecies_Gent27b
IMPORTED: RH_Tax_Order_UniRef50_allSpecies_Gent27c
IMPORTED: RH_Tax_Order_UniRef50_allSpecies_Gent28a
IMPORTED: RH_Tax_Order_UniRef50_allSpecies_Gent28b
IMPORTED: RH_Tax_Order_UniRef50_allSpecies_Gent28c
IMPORTED: RH_Tax_Order_UniRef50_allSpecies_Gent29a
IMPORTED: RH_Tax_Order_UniRef50_allSpecies_Gent29b
IMPORTED: RH_Tax_Order_UniRef50_allSpecies_Gent29c
IMPORTED: RH_Tax_Order_UniRef50_allSpecies_Gent30a
```

```
IMPORTED: RH_Tax_Order_UniRef50_allSpecies_Gent29a
IMPORTED: RH_Tax_Order_UniRef50_allSpecies_Gent29b
IMPORTED: RH_Tax_Order_UniRef50_allSpecies_Gent29c
IMPORTED: RH_Tax_Order_UniRef50_allSpecies_Gent30a
IMPORTED: RH_Tax_Order_UniRef50_allSpecies_Gent30b
IMPORTED: RH_Tax_Order_UniRef50_allSpecies_Gent30c
IMPORTED: RH_Tax_Order_UniRef50_allSpecies_Gent31a
IMPORTED: RH_Tax_Order_UniRef50_allSpecies_Gent31b
IMPORTED: RH_Tax_Order_UniRef50_allSpecies_Gent31c
IMPORTED: RH_Tax_Order_UniRef50_allSpecies_Gent32a
IMPORTED: RH_Tax_Order_UniRef50_allSpecies_Gent32b
IMPORTED: RH_Tax_Order_UniRef50_allSpecies_Gent32c
IMPORTED: RH_Tax_Order_UniRef50_allSpecies_Gent33a
IMPORTED: RH_Tax_Order_UniRef50_allSpecies_Gent33b
IMPORTED: RH_Tax_Order_UniRef50_allSpecies_Gent33c
IMPORTED: RH_Tax_Order_UniRef50_allSpecies_Gent34a
IMPORTED: RH_Tax_Order_UniRef50_allSpecies_Gent34b
IMPORTED: RH_Tax_Order_UniRef50_allSpecies_Gent34c
IMPORTED: RH_Tax_Order_UniRef50_allSpecies_Gent35a
IMPORTED: RH_Tax_Order_UniRef50_allSpecies_Gent35b
IMPORTED: RH_Tax_Order_UniRef50_allSpecies_Gent35c
IMPORTED: RH_Tax_Order_UniRef50_allSpecies_Gent36a
IMPORTED: RH_Tax_Order_UniRef50_allSpecies_Gent36b
IMPORTED: RH_Tax_Order_UniRef50_allSpecies_Gent36c
IMPORTED: RH_Tax_Order_UniRef50_allSpecies_Gent37a
IMPORTED: RH_Tax_Order_UniRef50_allSpecies_Gent37b
IMPORTED: RH_Tax_Order_UniRef50_allSpecies_Gent37c
IMPORTED: RH_Tax_Order_UniRef50_allSpecies_Gent38a
IMPORTED: RH_Tax_Order_UniRef50_allSpecies_Gent38b
IMPORTED: RH_Tax_Order_UniRef50_allSpecies_Gent38c
IMPORTED: RH_Tax_Order_UniRef50_allSpecies_Gent39a
IMPORTED: RH_Tax_Order_UniRef50_allSpecies_Gent39b
IMPORTED: RH_Tax_Order_UniRef50_allSpecies_Gent39c
IMPORTED: RH_Tax_Order_UniRef50_allSpecies_Gent40a
IMPORTED: RH_Tax_Order_UniRef50_allSpecies_Gent40b
IMPORTED: RH_Tax_Order_UniRef50_allSpecies_Gent40c
READ: RH_Tax_Order_UniRef50_allSpecies_Gent01a
READ: RH_Tax_Order_UniRef50_allSpecies_Gent01b
READ: RH_Tax_Order_UniRef50_allSpecies_Gent01c
READ: RH_Tax_Order_UniRef50_allSpecies_Gent02a
READ: RH_Tax_Order_UniRef50_allSpecies_Gent02b
READ: RH_Tax_Order_UniRef50_allSpecies_Gent02c
READ: RH_Tax_Order_UniRef50_allSpecies_Gent03a
READ: RH_Tax_Order_UniRef50_allSpecies_Gent03b
READ: RH_Tax_Order_UniRef50_allSpecies_Gent03c
READ: RH_Tax_Order_UniRef50_allSpecies_Gent04a
READ: RH_Tax_Order_UniRef50_allSpecies_Gent04b
READ: RH_Tax_Order_UniRef50_allSpecies_Gent04c
READ: RH_Tax_Order_UniRef50_allSpecies_Gent05a
READ: RH_Tax_Order_UniRef50_allSpecies_Gent05b
READ: RH_Tax_Order_UniRef50_allSpecies_Gent05c
READ: RH_Tax_Order_UniRef50_allSpecies_Gent06a
READ: RH_Tax_Order_UniRef50_allSpecies_Gent06b
READ: RH_Tax_Order_UniRef50_allSpecies_Gent06c
READ: RH_Tax_Order_UniRef50_allSpecies_Gent07a
READ: RH_Tax_Order_UniRef50_allSpecies_Gent07b
READ: RH_Tax_Order_UniRef50_allSpecies_Gent07c
READ: RH_Tax_Order_UniRef50_allSpecies_Gent08a
READ: RH_Tax_Order_UniRef50_allSpecies_Gent08b
READ: RH_Tax_Order_UniRef50_allSpecies_Gent08c
READ: RH_Tax_Order_UniRef50_allSpecies_Gent09a
READ: RH_Tax_Order_UniRef50_allSpecies_Gent09b
READ: RH_Tax_Order_UniRef50_allSpecies_Gent09c
READ: RH_Tax_Order_UniRef50_allSpecies_Gent10a
READ: RH_Tax_Order_UniRef50_allSpecies_Gent10b
```

```
READ: RH_Tax_Order_UniRef50_allSpecies_Gent09b
READ: RH_Tax_Order_UniRef50_allSpecies_Gent09c
READ: RH_Tax_Order_UniRef50_allSpecies_Gent10a
READ: RH_Tax_Order_UniRef50_allSpecies_Gent10b
READ: RH_Tax_Order_UniRef50_allSpecies_Gent10c
READ: RH_Tax_Order_UniRef50_allSpecies_Gent11a
READ: RH_Tax_Order_UniRef50_allSpecies_Gent11b
READ: RH_Tax_Order_UniRef50_allSpecies_Gent11c
READ: RH_Tax_Order_UniRef50_allSpecies_Gent12a
READ: RH_Tax_Order_UniRef50_allSpecies_Gent12b
READ: RH_Tax_Order_UniRef50_allSpecies_Gent12c
READ: RH_Tax_Order_UniRef50_allSpecies_Gent13a
READ: RH_Tax_Order_UniRef50_allSpecies_Gent13b
READ: RH_Tax_Order_UniRef50_allSpecies_Gent13c
READ: RH_Tax_Order_UniRef50_allSpecies_Gent14a
READ: RH_Tax_Order_UniRef50_allSpecies_Gent14b
READ: RH_Tax_Order_UniRef50_allSpecies_Gent14c
READ: RH_Tax_Order_UniRef50_allSpecies_Gent15a
READ: RH_Tax_Order_UniRef50_allSpecies_Gent15b
READ: RH_Tax_Order_UniRef50_allSpecies_Gent15c
READ: RH_Tax_Order_UniRef50_allSpecies_Gent16a
READ: RH_Tax_Order_UniRef50_allSpecies_Gent16b
READ: RH_Tax_Order_UniRef50_allSpecies_Gent16c
READ: RH_Tax_Order_UniRef50_allSpecies_Gent17a
READ: RH_Tax_Order_UniRef50_allSpecies_Gent17b
READ: RH_Tax_Order_UniRef50_allSpecies_Gent17c
READ: RH_Tax_Order_UniRef50_allSpecies_Gent18a
READ: RH_Tax_Order_UniRef50_allSpecies_Gent18b
READ: RH_Tax_Order_UniRef50_allSpecies_Gent18c
READ: RH_Tax_Order_UniRef50_allSpecies_Gent19a
READ: RH_Tax_Order_UniRef50_allSpecies_Gent19b
READ: RH_Tax_Order_UniRef50_allSpecies_Gent19c
READ: RH_Tax_Order_UniRef50_allSpecies_Gent20a
READ: RH_Tax_Order_UniRef50_allSpecies_Gent20b
READ: RH_Tax_Order_UniRef50_allSpecies_Gent20c
READ: RH_Tax_Order_UniRef50_allSpecies_Gent21a
READ: RH_Tax_Order_UniRef50_allSpecies_Gent21c
READ: RH_Tax_Order_UniRef50_allSpecies_Gent21d
READ: RH_Tax_Order_UniRef50_allSpecies_Gent22a
READ: RH_Tax_Order_UniRef50_allSpecies_Gent22b
READ: RH_Tax_Order_UniRef50_allSpecies_Gent22c
READ: RH_Tax_Order_UniRef50_allSpecies_Gent23a
READ: RH_Tax_Order_UniRef50_allSpecies_Gent23b
READ: RH_Tax_Order_UniRef50_allSpecies_Gent23c
READ: RH_Tax_Order_UniRef50_allSpecies_Gent24a
READ: RH_Tax_Order_UniRef50_allSpecies_Gent24b
READ: RH_Tax_Order_UniRef50_allSpecies_Gent24c
READ: RH_Tax_Order_UniRef50_allSpecies_Gent25a
READ: RH_Tax_Order_UniRef50_allSpecies_Gent25b
READ: RH_Tax_Order_UniRef50_allSpecies_Gent25c
READ: RH_Tax_Order_UniRef50_allSpecies_Gent26a
READ: RH_Tax_Order_UniRef50_allSpecies_Gent26b
READ: RH_Tax_Order_UniRef50_allSpecies_Gent26c
READ: RH_Tax_Order_UniRef50_allSpecies_Gent27a
READ: RH_Tax_Order_UniRef50_allSpecies_Gent27b
READ: RH_Tax_Order_UniRef50_allSpecies_Gent27c
READ: RH_Tax_Order_UniRef50_allSpecies_Gent28a
READ: RH_Tax_Order_UniRef50_allSpecies_Gent28b
READ: RH_Tax_Order_UniRef50_allSpecies_Gent28c
READ: RH_Tax_Order_UniRef50_allSpecies_Gent29a
READ: RH_Tax_Order_UniRef50_allSpecies_Gent29b
READ: RH_Tax_Order_UniRef50_allSpecies_Gent29c
READ: RH_Tax_Order_UniRef50_allSpecies_Gent30a
READ: RH_Tax_Order_UniRef50_allSpecies_Gent30b
READ: RH_Tax_Order_UniRef50_allSpecies_Gent30c
```

```
[illegible]
READ: RH_Tax_Order_UniRef50_allSpecies_Gent30a
READ: RH_Tax_Order_UniRef50_allSpecies_Gent30b
READ: RH_Tax_Order_UniRef50_allSpecies_Gent30c
READ: RH_Tax_Order_UniRef50_allSpecies_Gent31a
READ: RH_Tax_Order_UniRef50_allSpecies_Gent31b
READ: RH_Tax_Order_UniRef50_allSpecies_Gent31c
READ: RH_Tax_Order_UniRef50_allSpecies_Gent32a
READ: RH_Tax_Order_UniRef50_allSpecies_Gent32b
READ: RH_Tax_Order_UniRef50_allSpecies_Gent32c
READ: RH_Tax_Order_UniRef50_allSpecies_Gent33a
READ: RH_Tax_Order_UniRef50_allSpecies_Gent33b
READ: RH_Tax_Order_UniRef50_allSpecies_Gent33c
READ: RH_Tax_Order_UniRef50_allSpecies_Gent34a
READ: RH_Tax_Order_UniRef50_allSpecies_Gent34b
READ: RH_Tax_Order_UniRef50_allSpecies_Gent34c
READ: RH_Tax_Order_UniRef50_allSpecies_Gent35a
READ: RH_Tax_Order_UniRef50_allSpecies_Gent35b
READ: RH_Tax_Order_UniRef50_allSpecies_Gent35c
READ: RH_Tax_Order_UniRef50_allSpecies_Gent36a
READ: RH_Tax_Order_UniRef50_allSpecies_Gent36b
READ: RH_Tax_Order_UniRef50_allSpecies_Gent36c
READ: RH_Tax_Order_UniRef50_allSpecies_Gent37a
READ: RH_Tax_Order_UniRef50_allSpecies_Gent37b
READ: RH_Tax_Order_UniRef50_allSpecies_Gent37c
READ: RH_Tax_Order_UniRef50_allSpecies_Gent38a
READ: RH_Tax_Order_UniRef50_allSpecies_Gent38b
READ: RH_Tax_Order_UniRef50_allSpecies_Gent38c
READ: RH_Tax_Order_UniRef50_allSpecies_Gent39a
READ: RH_Tax_Order_UniRef50_allSpecies_Gent39b
READ: RH_Tax_Order_UniRef50_allSpecies_Gent39c
READ: RH_Tax_Order_UniRef50_allSpecies_Gent40a
READ: RH_Tax_Order_UniRef50_allSpecies_Gent40b
READ: RH_Tax_Order_UniRef50_allSpecies_Gent40c
Taxonomy files successfully read:
Matrix was normalized and filtered by threshold 0.01 successfully
CrossCorrelation with Spearman matrix successfully calculated
```

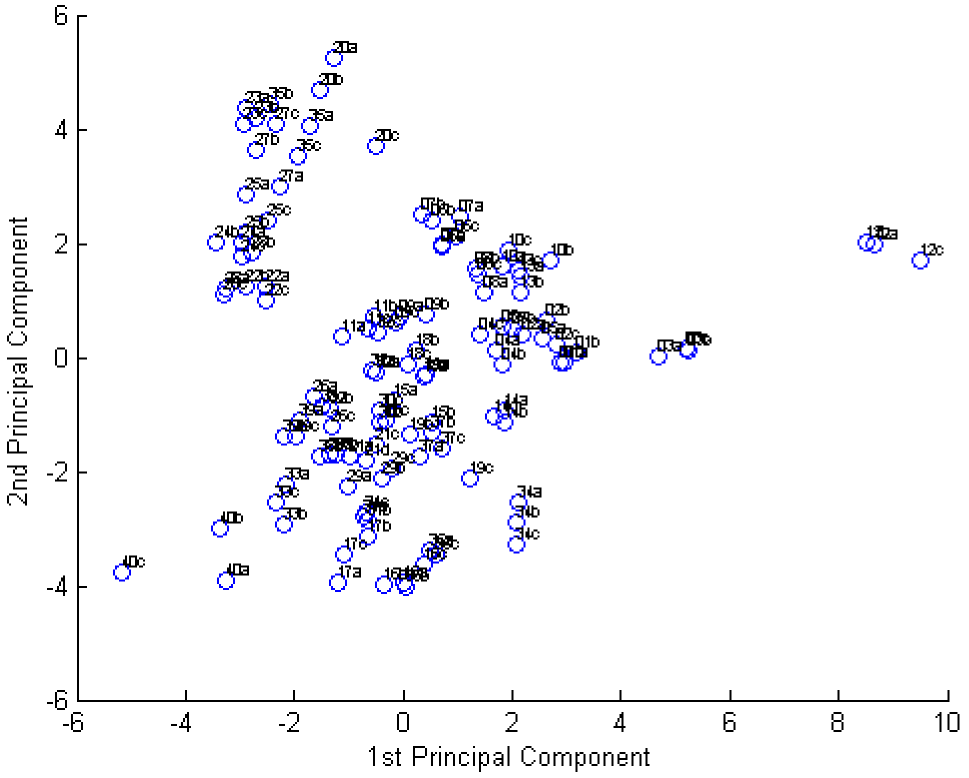

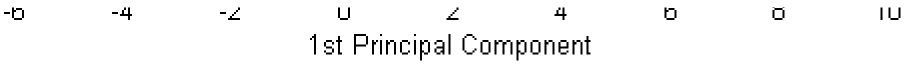
-6
-4
-2
0
2
4
6
8
10
1st Principal Component

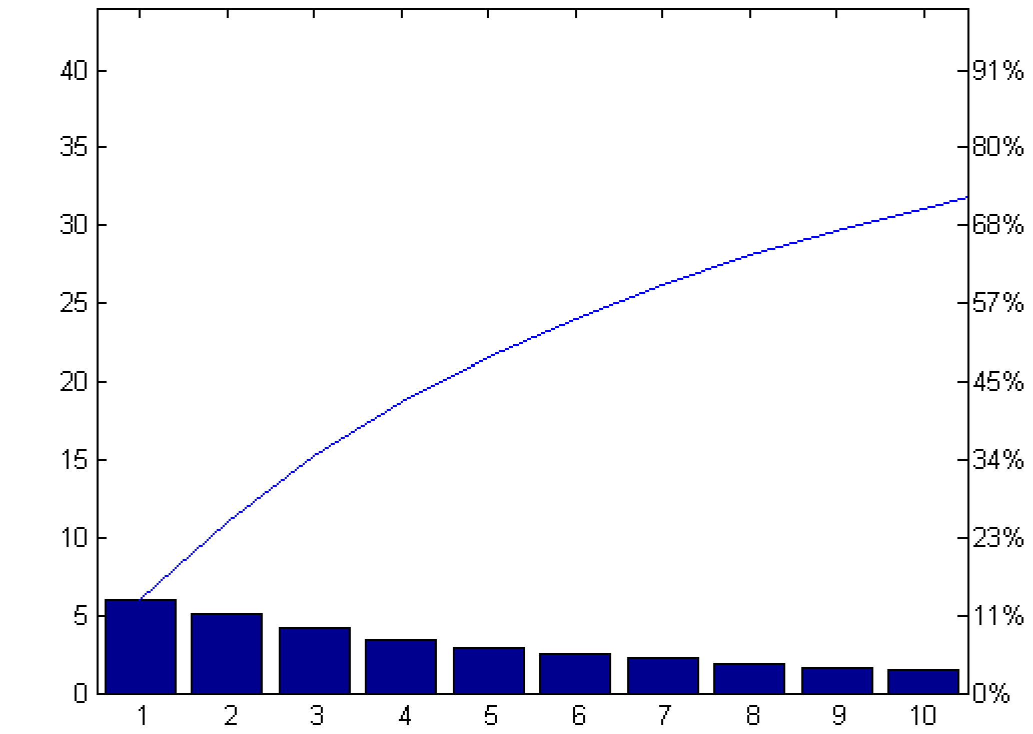
40
35
30
25
20
15
10
5
0
91%
80%
68%
57%
45%
34%
23%
11%
0%
1
2
3
4
5
6
7
8
9
10

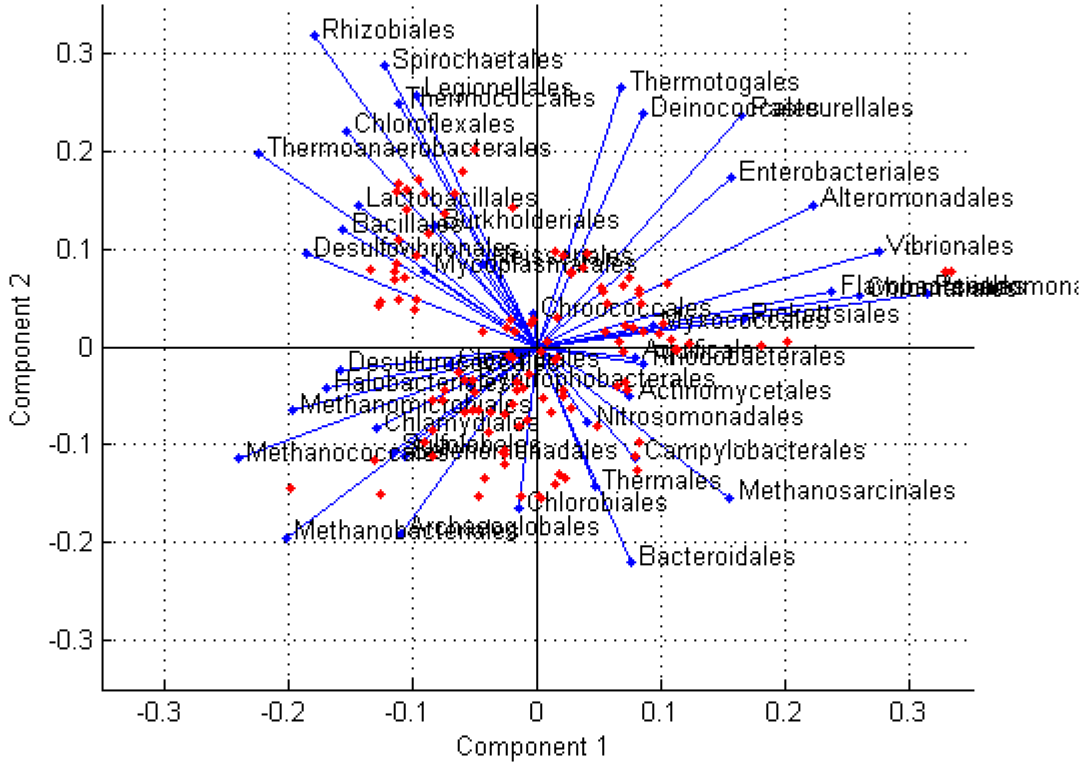
Rhizobiales
Spirochaetales
Legionellales
Thermococcales
Chloroflexales
Thermoanaerobacterales
Lactobacillales
Bacillales
Burkholderiales
Desulfovibrionales
Mycoplasmatales
Thermotogales
Deinococcales
Pasteurellales
Enterobacteriales
Alteromonadales
Vibrionales
Chroococcales
Myxococcales
Rhodobacterales
Actinomycetales
Nitrosomonadales
Campylobacterales
Thermales
Methanosarcinales
Chlorobiales
Bacteroidales
Halobacteriales
Methanomicrobiales
Chlamydiales
Methanococcales
Methanobacteriales
Archaeoglobales
Component 1
Component 2
0.3
0.2
0.1
0
-0.1
-0.2
-0.3

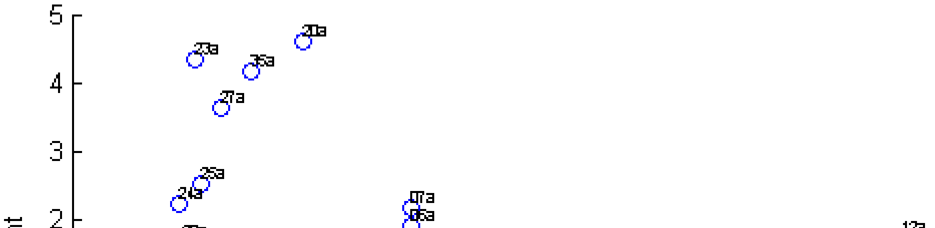
5
4
3
2
23a
20a
35a
27a
25a
24a
07a
06a

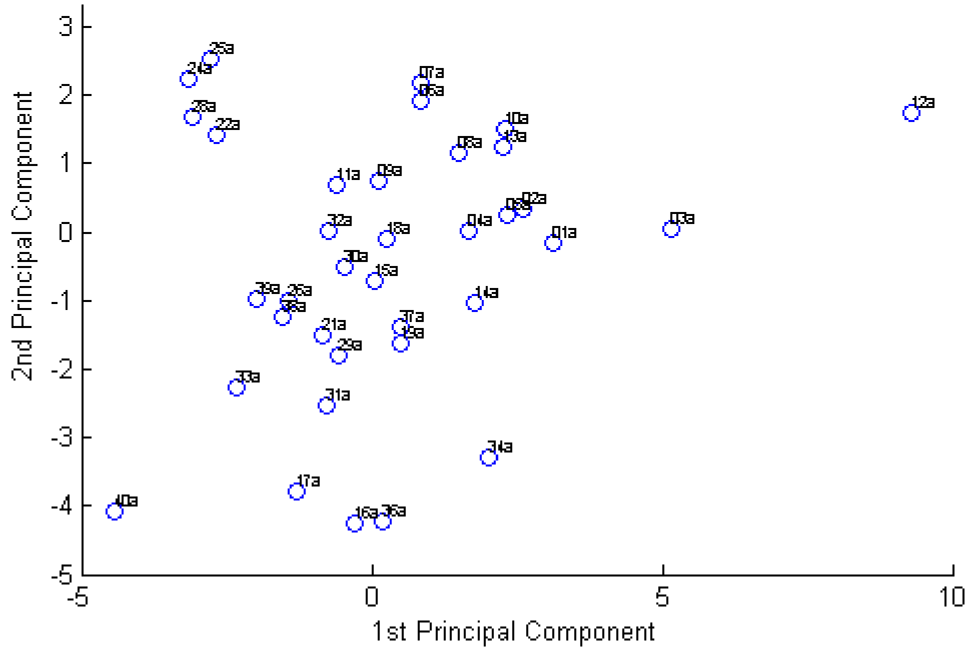
2nd Principal Component
1st Principal Component
3
2
1
0
-1
-2
-3
-4
-5
-5
0
5
10
25a
24a
23a
22a
07a
10a
13a
08a
11a
09a
02a
03a
32a
18a
04a
01a
30a
15a
39a
26a
21a
37a
29a
14a
33a
31a
34a
17a
40a
16a
35a
12a

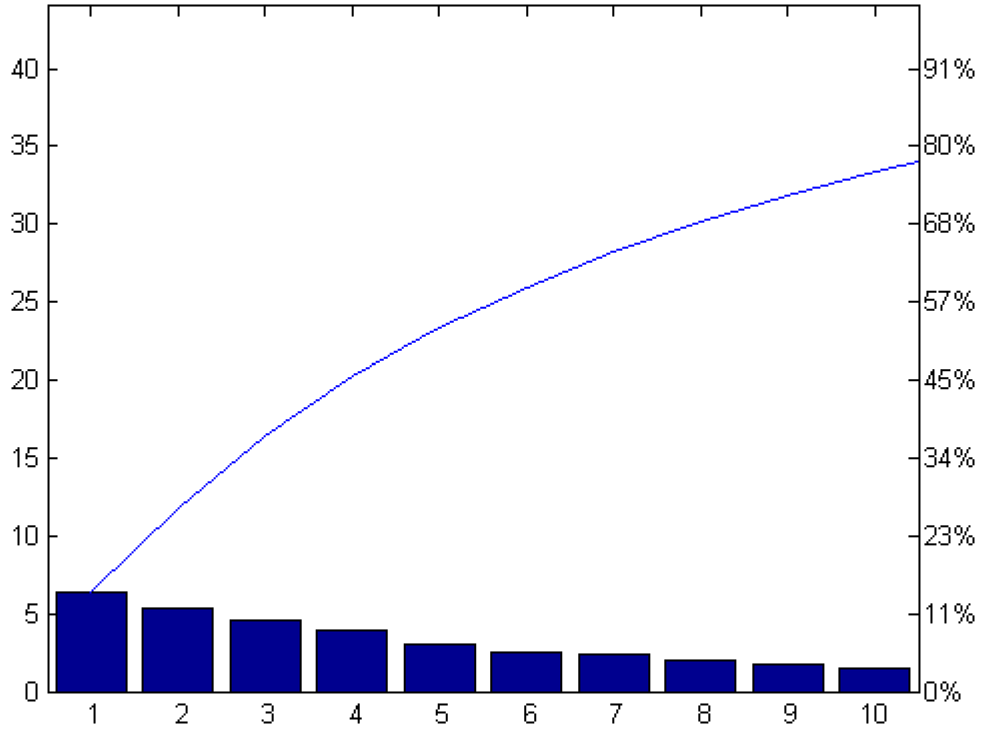
40
35
30
25
20
15
10
5
0
91%
80%
68%
57%
45%
34%
23%
11%
0%
1
2
3
4
5
6
7
8
9
10

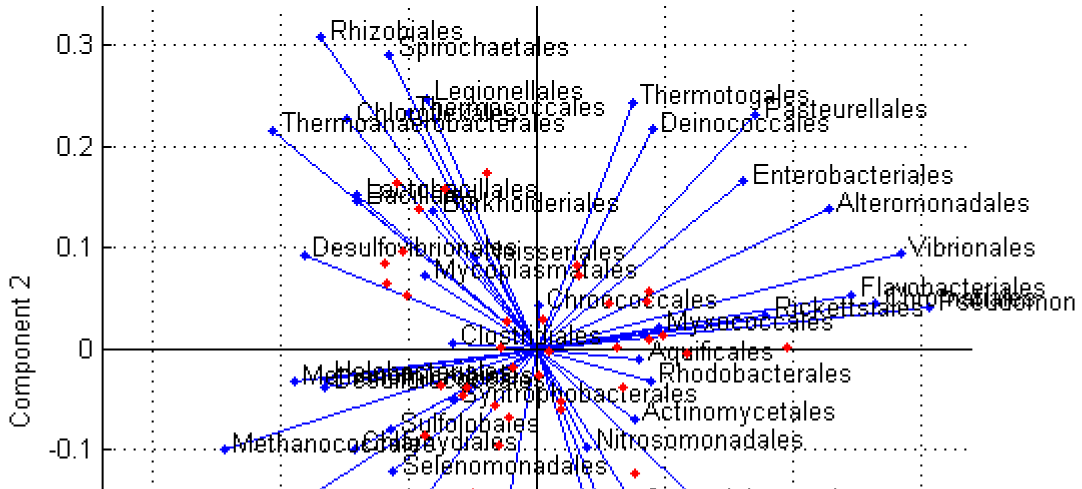
Component 2
0.3
0.2
0.1
0
-0.1
Rhizobiales
Spirochaetales
Legionellales
Thermotogales
Pasteurellales
Thermoanaerobacterales
Deinococcales
Enterobacteriales
Burkholderiales
Alteromonadales
Vibrionales
Neisseriales
Mycoplasmatales
Flavobacteriales
Chroococcales
Rickettsiales
Myxococcales
Clostridiales
Aquificales
Rhodobacterales
Syntrophobacterales
Actinomycetales
Sulfolobales
Nitrosomonadales
Selenomonadales
Campylobacterales

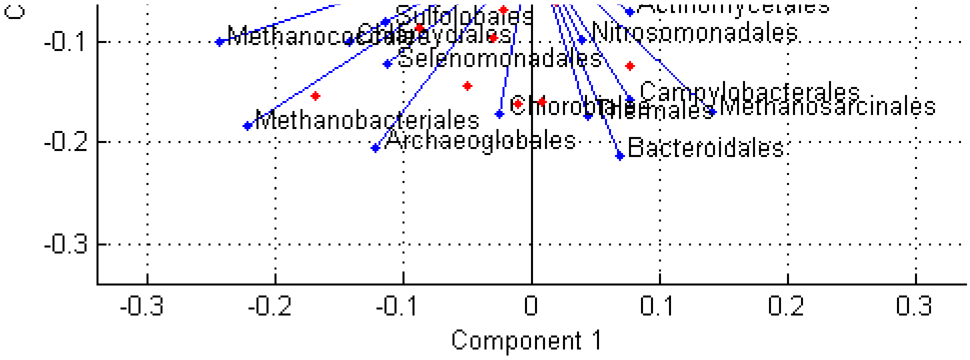

Process data

1. Read process data rows Variables, cols: BGPs (original matrix is transposed)

```
 [meta_matrix] = ReadMetaData();

% 3. Calculate Cross Correlations
[meta_correlationMatrix] = CalculateCorrelation_V2(meta_matrix, meta_matrix);
```

```
CrossCorrelation with Spearman matrix successfully calculated
```

Biological function Ontology

```
% 1. Read biological functions
 [onto_matrix_original] = ReadBiolFunctFiles();

% 2. Shorten the entry in the type list
for entry = 2: size(onto_matrix_original, 2)
    str = cell2mat(onto_matrix_original(1, entry));
    onto_matrix_original(1, entry) = {regexprep(str,'Gent_Onto_BiolFunction_UniRef50_allSpeci
end
clearvars str, entry;

% 3. Filter and normalize the onto_matrix
[onto_matrix_filtered] = FilterAndNormalize(onto_matrix_original, 0.01);

% 4. Create average matrix
[onto_average_matrix_filtered]= AverageMatrix(onto_matrix_filtered);

% 5. The correlation matrix
[ onto_correlationMatrix2 ] = CalculateCorrelation_V2(onto_average_matrix_filtered, onto_aver

% 6. Perform PCA
[coeff,Scores,latent]= PerformPCA(onto_matrix_filtered, onto_matrix_filtered(1, 2:end),'Onto_
PerformPCA(onto_average_matrix_filtered, onto_average_matrix_filtered(1, 2:end),'Onto_Norm');
```

```
IMPORTED: Gent_Onto_BiolFunction_UniRef50_allSpecies_Gent01a
IMPORTED: Gent_Onto_BiolFunction_UniRef50_allSpecies_Gent01b
IMPORTED: Gent_Onto_BiolFunction_UniRef50_allSpecies_Gent01c
IMPORTED: Gent_Onto_BiolFunction_UniRef50_allSpecies_Gent02a
IMPORTED: Gent_Onto_BiolFunction_UniRef50_allSpecies_Gent02b
IMPORTED: Gent_Onto_BiolFunction_UniRef50_allSpecies_Gent02c
IMPORTED: Gent_Onto_BiolFunction_UniRef50_allSpecies_Gent03a
IMPORTED: Gent_Onto_BiolFunction_UniRef50_allSpecies_Gent03b
IMPORTED: Gent_Onto_BiolFunction_UniRef50_allSpecies_Gent03c
IMPORTED: Gent_Onto_BiolFunction_UniRef50_allSpecies_Gent04a
IMPORTED: Gent_Onto_BiolFunction_UniRef50_allSpecies_Gent04b
```

```
IMPORTED: Gent_Onto_BiolFunction_UniRef50_allSpecies_Gent03c
IMPORTED: Gent_Onto_BiolFunction_UniRef50_allSpecies_Gent04a
IMPORTED: Gent_Onto_BiolFunction_UniRef50_allSpecies_Gent04b
IMPORTED: Gent_Onto_BiolFunction_UniRef50_allSpecies_Gent04c
IMPORTED: Gent_Onto_BiolFunction_UniRef50_allSpecies_Gent05a
IMPORTED: Gent_Onto_BiolFunction_UniRef50_allSpecies_Gent05b
IMPORTED: Gent_Onto_BiolFunction_UniRef50_allSpecies_Gent05c
IMPORTED: Gent_Onto_BiolFunction_UniRef50_allSpecies_Gent06a
IMPORTED: Gent_Onto_BiolFunction_UniRef50_allSpecies_Gent06b
IMPORTED: Gent_Onto_BiolFunction_UniRef50_allSpecies_Gent06c
IMPORTED: Gent_Onto_BiolFunction_UniRef50_allSpecies_Gent07a
IMPORTED: Gent_Onto_BiolFunction_UniRef50_allSpecies_Gent07b
IMPORTED: Gent_Onto_BiolFunction_UniRef50_allSpecies_Gent07c
IMPORTED: Gent_Onto_BiolFunction_UniRef50_allSpecies_Gent08a
IMPORTED: Gent_Onto_BiolFunction_UniRef50_allSpecies_Gent08b
IMPORTED: Gent_Onto_BiolFunction_UniRef50_allSpecies_Gent08c
IMPORTED: Gent_Onto_BiolFunction_UniRef50_allSpecies_Gent09a
IMPORTED: Gent_Onto_BiolFunction_UniRef50_allSpecies_Gent09b
IMPORTED: Gent_Onto_BiolFunction_UniRef50_allSpecies_Gent09c
IMPORTED: Gent_Onto_BiolFunction_UniRef50_allSpecies_Gent10a
IMPORTED: Gent_Onto_BiolFunction_UniRef50_allSpecies_Gent10b
IMPORTED: Gent_Onto_BiolFunction_UniRef50_allSpecies_Gent10c
IMPORTED: Gent_Onto_BiolFunction_UniRef50_allSpecies_Gent11a
IMPORTED: Gent_Onto_BiolFunction_UniRef50_allSpecies_Gent11b
IMPORTED: Gent_Onto_BiolFunction_UniRef50_allSpecies_Gent11c
IMPORTED: Gent_Onto_BiolFunction_UniRef50_allSpecies_Gent12a
IMPORTED: Gent_Onto_BiolFunction_UniRef50_allSpecies_Gent12b
IMPORTED: Gent_Onto_BiolFunction_UniRef50_allSpecies_Gent12c
IMPORTED: Gent_Onto_BiolFunction_UniRef50_allSpecies_Gent13a
IMPORTED: Gent_Onto_BiolFunction_UniRef50_allSpecies_Gent13b
IMPORTED: Gent_Onto_BiolFunction_UniRef50_allSpecies_Gent13c
IMPORTED: Gent_Onto_BiolFunction_UniRef50_allSpecies_Gent14a
IMPORTED: Gent_Onto_BiolFunction_UniRef50_allSpecies_Gent14b
IMPORTED: Gent_Onto_BiolFunction_UniRef50_allSpecies_Gent14c
IMPORTED: Gent_Onto_BiolFunction_UniRef50_allSpecies_Gent15a
IMPORTED: Gent_Onto_BiolFunction_UniRef50_allSpecies_Gent15b
IMPORTED: Gent_Onto_BiolFunction_UniRef50_allSpecies_Gent15c
IMPORTED: Gent_Onto_BiolFunction_UniRef50_allSpecies_Gent16a
IMPORTED: Gent_Onto_BiolFunction_UniRef50_allSpecies_Gent16b
IMPORTED: Gent_Onto_BiolFunction_UniRef50_allSpecies_Gent16c
IMPORTED: Gent_Onto_BiolFunction_UniRef50_allSpecies_Gent17a
IMPORTED: Gent_Onto_BiolFunction_UniRef50_allSpecies_Gent17b
IMPORTED: Gent_Onto_BiolFunction_UniRef50_allSpecies_Gent17c
IMPORTED: Gent_Onto_BiolFunction_UniRef50_allSpecies_Gent18a
IMPORTED: Gent_Onto_BiolFunction_UniRef50_allSpecies_Gent18b
IMPORTED: Gent_Onto_BiolFunction_UniRef50_allSpecies_Gent18c
IMPORTED: Gent_Onto_BiolFunction_UniRef50_allSpecies_Gent19a
IMPORTED: Gent_Onto_BiolFunction_UniRef50_allSpecies_Gent19b
IMPORTED: Gent_Onto_BiolFunction_UniRef50_allSpecies_Gent19c
IMPORTED: Gent_Onto_BiolFunction_UniRef50_allSpecies_Gent20a
IMPORTED: Gent_Onto_BiolFunction_UniRef50_allSpecies_Gent20b
IMPORTED: Gent_Onto_BiolFunction_UniRef50_allSpecies_Gent20c
IMPORTED: Gent_Onto_BiolFunction_UniRef50_allSpecies_Gent21a
IMPORTED: Gent_Onto_BiolFunction_UniRef50_allSpecies_Gent21c
IMPORTED: Gent_Onto_BiolFunction_UniRef50_allSpecies_Gent21d
IMPORTED: Gent_Onto_BiolFunction_UniRef50_allSpecies_Gent22a
IMPORTED: Gent_Onto_BiolFunction_UniRef50_allSpecies_Gent22b
IMPORTED: Gent_Onto_BiolFunction_UniRef50_allSpecies_Gent22c
IMPORTED: Gent_Onto_BiolFunction_UniRef50_allSpecies_Gent23a
IMPORTED: Gent_Onto_BiolFunction_UniRef50_allSpecies_Gent23b
IMPORTED: Gent_Onto_BiolFunction_UniRef50_allSpecies_Gent23c
IMPORTED: Gent_Onto_BiolFunction_UniRef50_allSpecies_Gent24a
IMPORTED: Gent_Onto_BiolFunction_UniRef50_allSpecies_Gent24b
IMPORTED: Gent_Onto_BiolFunction_UniRef50_allSpecies_Gent24c
[illegible]
```

```
IMPORTED: Gent_Onto_BiolFunction_UniRef50_allSpecies_Gent24a
IMPORTED: Gent_Onto_BiolFunction_UniRef50_allSpecies_Gent24b
IMPORTED: Gent_Onto_BiolFunction_UniRef50_allSpecies_Gent24c
IMPORTED: Gent_Onto_BiolFunction_UniRef50_allSpecies_Gent25a
IMPORTED: Gent_Onto_BiolFunction_UniRef50_allSpecies_Gent25b
IMPORTED: Gent_Onto_BiolFunction_UniRef50_allSpecies_Gent25c
IMPORTED: Gent_Onto_BiolFunction_UniRef50_allSpecies_Gent26a
IMPORTED: Gent_Onto_BiolFunction_UniRef50_allSpecies_Gent26b
IMPORTED: Gent_Onto_BiolFunction_UniRef50_allSpecies_Gent26c
IMPORTED: Gent_Onto_BiolFunction_UniRef50_allSpecies_Gent27a
IMPORTED: Gent_Onto_BiolFunction_UniRef50_allSpecies_Gent27b
IMPORTED: Gent_Onto_BiolFunction_UniRef50_allSpecies_Gent27c
IMPORTED: Gent_Onto_BiolFunction_UniRef50_allSpecies_Gent28a
IMPORTED: Gent_Onto_BiolFunction_UniRef50_allSpecies_Gent28b
IMPORTED: Gent_Onto_BiolFunction_UniRef50_allSpecies_Gent28c
IMPORTED: Gent_Onto_BiolFunction_UniRef50_allSpecies_Gent29a
IMPORTED: Gent_Onto_BiolFunction_UniRef50_allSpecies_Gent29b
IMPORTED: Gent_Onto_BiolFunction_UniRef50_allSpecies_Gent29c
IMPORTED: Gent_Onto_BiolFunction_UniRef50_allSpecies_Gent30a
IMPORTED: Gent_Onto_BiolFunction_UniRef50_allSpecies_Gent30b
IMPORTED: Gent_Onto_BiolFunction_UniRef50_allSpecies_Gent30c
IMPORTED: Gent_Onto_BiolFunction_UniRef50_allSpecies_Gent31a
IMPORTED: Gent_Onto_BiolFunction_UniRef50_allSpecies_Gent31b
IMPORTED: Gent_Onto_BiolFunction_UniRef50_allSpecies_Gent31c
IMPORTED: Gent_Onto_BiolFunction_UniRef50_allSpecies_Gent32a
IMPORTED: Gent_Onto_BiolFunction_UniRef50_allSpecies_Gent32b
IMPORTED: Gent_Onto_BiolFunction_UniRef50_allSpecies_Gent32c
IMPORTED: Gent_Onto_BiolFunction_UniRef50_allSpecies_Gent33a
IMPORTED: Gent_Onto_BiolFunction_UniRef50_allSpecies_Gent33b
IMPORTED: Gent_Onto_BiolFunction_UniRef50_allSpecies_Gent33c
IMPORTED: Gent_Onto_BiolFunction_UniRef50_allSpecies_Gent34a
IMPORTED: Gent_Onto_BiolFunction_UniRef50_allSpecies_Gent34b
IMPORTED: Gent_Onto_BiolFunction_UniRef50_allSpecies_Gent34c
IMPORTED: Gent_Onto_BiolFunction_UniRef50_allSpecies_Gent35a
IMPORTED: Gent_Onto_BiolFunction_UniRef50_allSpecies_Gent35b
IMPORTED: Gent_Onto_BiolFunction_UniRef50_allSpecies_Gent35c
IMPORTED: Gent_Onto_BiolFunction_UniRef50_allSpecies_Gent36a
IMPORTED: Gent_Onto_BiolFunction_UniRef50_allSpecies_Gent36b
IMPORTED: Gent_Onto_BiolFunction_UniRef50_allSpecies_Gent36c
IMPORTED: Gent_Onto_BiolFunction_UniRef50_allSpecies_Gent37a
IMPORTED: Gent_Onto_BiolFunction_UniRef50_allSpecies_Gent37b
IMPORTED: Gent_Onto_BiolFunction_UniRef50_allSpecies_Gent37c
IMPORTED: Gent_Onto_BiolFunction_UniRef50_allSpecies_Gent38a
IMPORTED: Gent_Onto_BiolFunction_UniRef50_allSpecies_Gent38b
IMPORTED: Gent_Onto_BiolFunction_UniRef50_allSpecies_Gent38c
IMPORTED: Gent_Onto_BiolFunction_UniRef50_allSpecies_Gent39a
IMPORTED: Gent_Onto_BiolFunction_UniRef50_allSpecies_Gent39b
IMPORTED: Gent_Onto_BiolFunction_UniRef50_allSpecies_Gent39c
IMPORTED: Gent_Onto_BiolFunction_UniRef50_allSpecies_Gent40a
IMPORTED: Gent_Onto_BiolFunction_UniRef50_allSpecies_Gent40b
IMPORTED: Gent_Onto_BiolFunction_UniRef50_allSpecies_Gent40c
Ontology files successfully read:
Matrix was normalized and filtered by threshold 0.01 successfully
CrossCorrelation with Spearman matrix successfully calculated
```

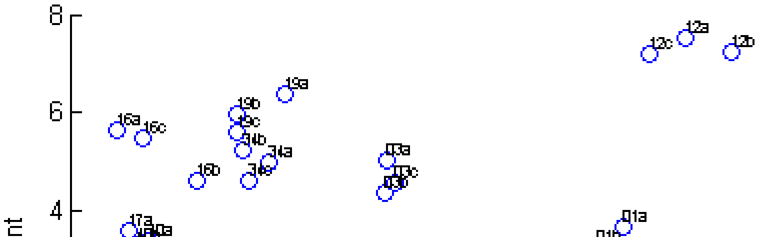

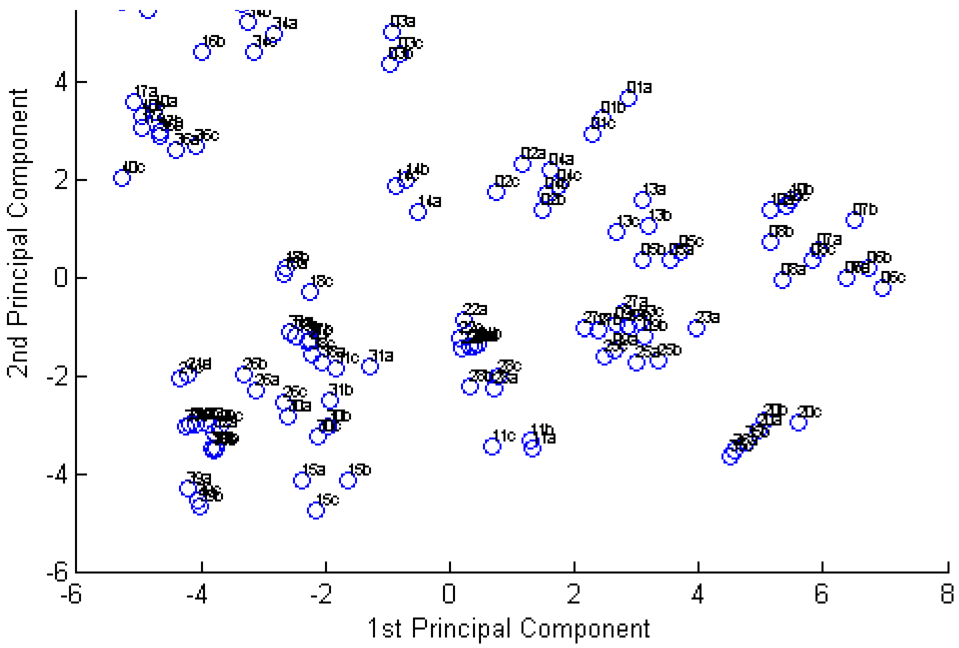
2nd Principal Component
1st Principal Component
4
2
0
-2
-4
-6
-6
-4
-2
0
2
4
6
8

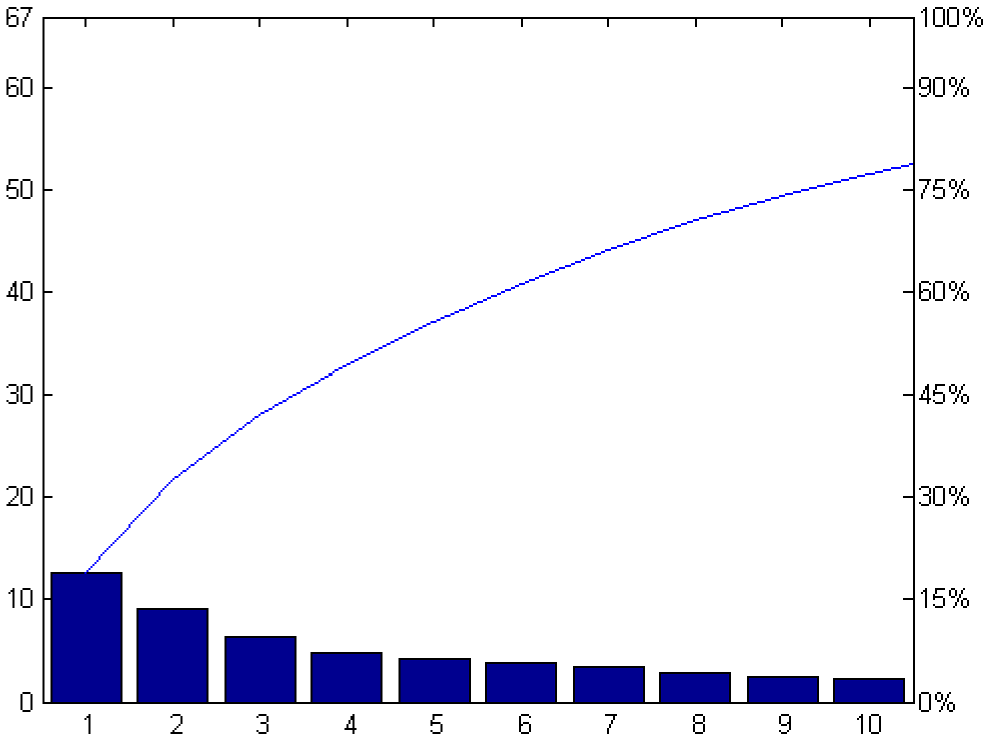
67
60
50
40
30
20
10
0
100%
90%
75%
60%
45%
30%
15%
0%
1
2
3
4
5
6
7
8
9
10

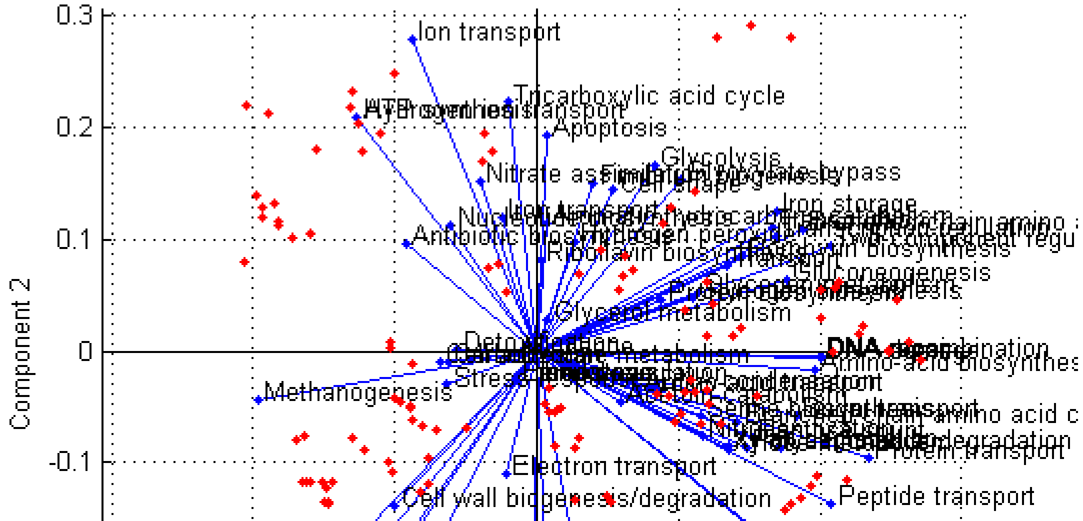
Component 2
0.3
0.2
0.1
0
-0.1
Ion transport
Tricarboxylic acid cycle
Apoptosis
Glycolysis
Iron storage
Riboflavin biosynthesis
Gluconeogenesis
Glycerol metabolism
Methanogenesis
Electron transport
Cell wall biogenesis/degradation
Peptide transport

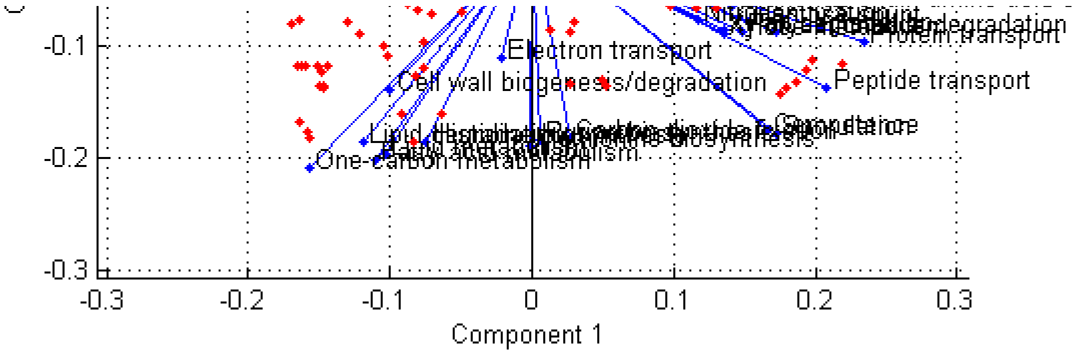
Electron transport
Cell wall biogenesis/degradation
Peptide transport
One-carbon metabolism
-0.1
-0.2
-0.3
-0.3
-0.2
-0.1
0
0.1
0.2
0.3
Component 1

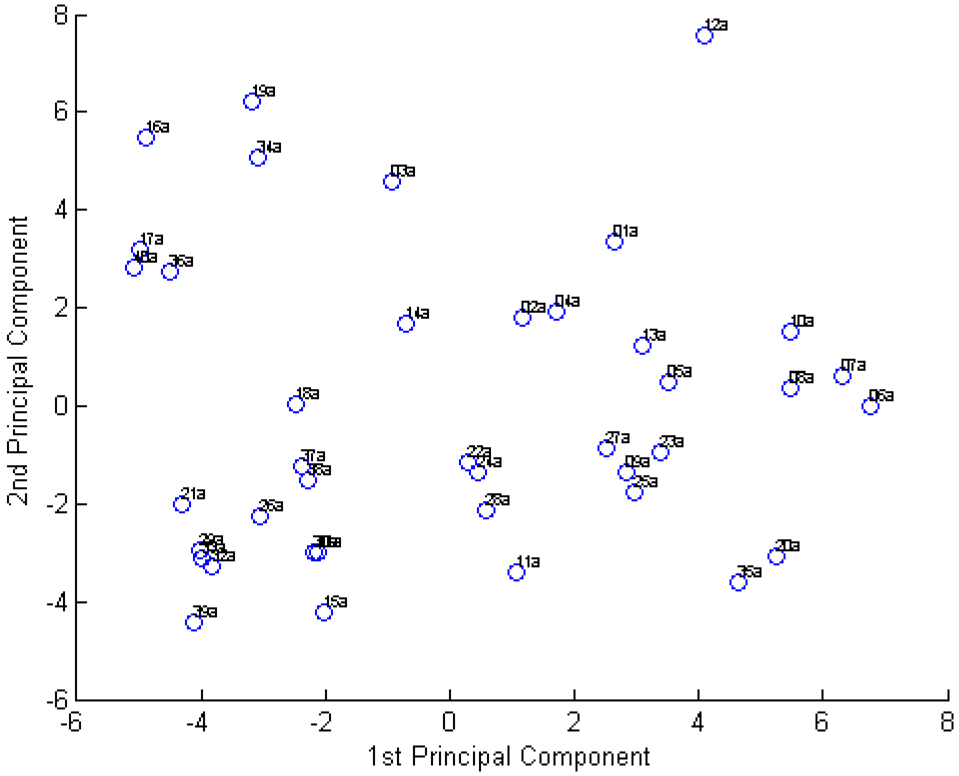
2nd Principal Component
1st Principal Component
8
6
4
2
0
-2
-4
-6
-6
-4
-2
0
2
4
6
8

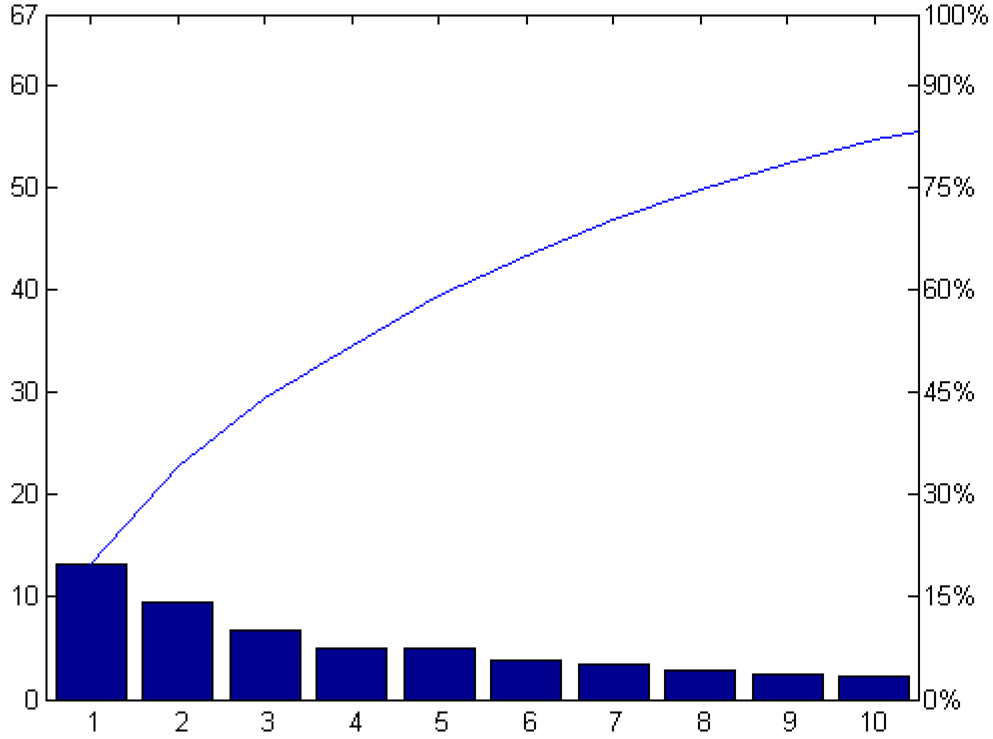
67
60
50
40
30
20
10
0
100%
90%
75%
60%
45%
30%
15%
0%
1
2
3
4
5
6
7
8
9
10

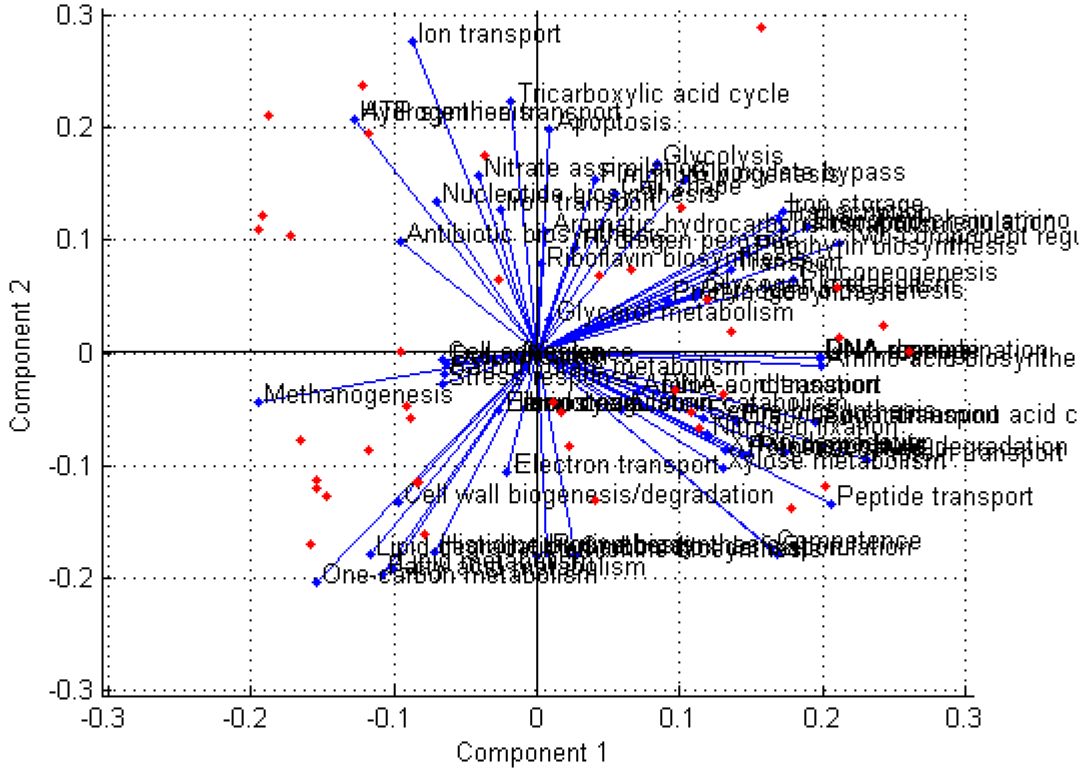

Metaprotein Data

```
% 1. Read metaprotein data
[mp_matrix_original, MatrixPlusDescription] = ReadMetaProteinFiles();

% 2. Shorten the entry in the type list and shorten the identifier
for entry = 2: size(mp_matrix_original, 2)
    str = cell2mat(mp_matrix_original(1, entry));
    mp_matrix_original(1, entry) = {regexprep(str,'Gent_MP_UniRef50_allSpecies_Gent','')} ;
end
clearvars str entry;

% 3. Remove "Unknown"-entry
for unknown = 1: size(MatrixPlusDescription, 1)
    if (strcmp(MatrixPlusDescription(unknown,1),  'UNKNOWN'))
        mp_matrix_original(unknown,:) = [];
        MatrixPlusDescription(unknown,:) = [];
        break;       % 'UNKNOWN' is only once in the dataset
    end
end
clearvars unknown;

% 4. Remove "Keratin"-entries and shorten the identifier
remove = [];
for filtered = 2: size(MatrixPlusDescription, 1)
    if (isempty(strfind(cell2mat(MatrixPlusDescription(filtered,2)),'Keratin')) == false);
        remove(end+1,1) = filtered;
    end
    % Shorten the identifer by removing UniRef50_
    str = cell2mat(MatrixPlusDescription(filtered,1));
    MatrixPlusDescription(filtered,1) = {regexprep(str,'UniRef50_','')};
    mp_matrix_original(filtered,1) = {regexprep(str,'UniRef50_','')};
end
remove = sort(remove, 'descend');
```

```
    mp_matrix_original(filtered,1) = {regexprep(str,'UniRef50_','')};
end
remove = sort(remove, 'descend');
for r = 1 :length(remove)
    mp_matrix_original(remove(r),:) = [];
    MatrixPlusDescription(remove(r),:) = [];
end
clearvars unknown r  remove filtered;

% 5. Filter and normalize the mp_matrix
[mp_matrix_filtered] = FilterAndNormalize(mp_matrix_original, 0.01);
mp_matrix_filtered_desc = cell(size(mp_matrix_filtered,1), size(mp_matrix_filtered,2)+14);
mp_matrix_filtered_desc(:,1) = mp_matrix_filtered(:,1);
mp_matrix_filtered_desc(:,16:end) = mp_matrix_filtered(:,2:end);

% 6. Create average matrix
[mp_average_matrix_filtered]= AverageMatrix(mp_matrix_filtered);
% Add description to the mp_average_matrix
mp_average_matrix_filtered_desc = cell(size(mp_average_matrix_filtered,1), size(mp_average_ma
mp_average_matrix_filtered_desc(:,1) = mp_average_matrix_filtered(:,1);
mp_average_matrix_filtered_desc(:,16:end) = mp_average_matrix_filtered(:,2:end);
% % Add Metainformation
for mp = 2: size(mp_average_matrix_filtered,1)
   for entries = 2 : size(MatrixPlusDescription, 1)
       if (isequal(mp_average_matrix_filtered_desc(mp,1), MatrixPlusDescription(entries,1)) )
           mp_average_matrix_filtered_desc(mp,2) = MatrixPlusDescription(entries,2); % Descri
           mp_average_matrix_filtered_desc(mp,3) = MatrixPlusDescription(entries,3); % Taxono
           mp_average_matrix_filtered_desc(mp,4) = MatrixPlusDescription(entries,4); % Superk
           mp_average_matrix_filtered_desc(mp,5) = MatrixPlusDescription(entries,5); % Kingdo
           mp_average_matrix_filtered_desc(mp,6) = MatrixPlusDescription(entries,6); % Phylum
           mp_average_matrix_filtered_desc(mp,7) = MatrixPlusDescription(entries,7); % Class
           mp_average_matrix_filtered_desc(mp,8) = MatrixPlusDescription(entries,8); % Order
           mp_average_matrix_filtered_desc(mp,9) = MatrixPlusDescription(entries,9); % Famili
           mp_average_matrix_filtered_desc(mp,10) = MatrixPlusDescription(entries,10); % Genu
           mp_average_matrix_filtered_desc(mp,11) = MatrixPlusDescription(entries,11); % Spec
           mp_average_matrix_filtered_desc(mp,12) = MatrixPlusDescription(entries,12); % UniR
           mp_average_matrix_filtered_desc(mp,13) = MatrixPlusDescription(entries,13); % UniR
           mp_average_matrix_filtered_desc(mp,14) = MatrixPlusDescription(entries,14); % KO
           mp_average_matrix_filtered_desc(mp,15) = MatrixPlusDescription(entries,15); % EC
       end
   end
end
clearvars mp entries;

% 7. The correlation matrix
 [ mp_correlationMatrix2 ] = CalculateCorrelation_V2(mp_average_matrix_filtered, mp_average_m
%  Add description to the correlation matrix
mp_correlationMatrix2_desc = cell(size(mp_correlationMatrix2,1), size(mp_correlationMatrix2,2
mp_correlationMatrix2_desc(:,1:15) = mp_average_matrix_filtered_desc(:,1:15);
mp_correlationMatrix2_desc(:,16:end) = mp_correlationMatrix2(:,2:end);

% 8. Perform PCA
[coeff,Scores,latent]=PerformPCA(mp_matrix_filtered, mp_matrix_filtered(1, 2:end),'MP_');
PerformPCA(mp_average_matrix_filtered, mp_average_matrix_filtered(1, 2:end),'MP_Norm');

% Perform Clustergram
 figure('Name', 'MP:Clustergram');
 clustergram(zscore(cell2mat(mp_matrix_filtered(2:end, 2:end))),  'RowLabels', mp_matrix_filt
```

```
IMPORTED: Gent_MP_UniRef50_allSpecies_Gent01a
IMPORTED: Gent_MP_UniRef50_allSpecies_Gent01b
IMPORTED: Gent_MP_UniRef50_allSpecies_Gent01c
IMPORTED: Gent_MP_UniRef50_allSpecies_Gent02a
```

```
[illegible]
IMPORTED: Gent_MP_UniRef50_allSpecies_Gent01b
IMPORTED: Gent_MP_UniRef50_allSpecies_Gent01c
IMPORTED: Gent_MP_UniRef50_allSpecies_Gent02a
IMPORTED: Gent_MP_UniRef50_allSpecies_Gent02b
IMPORTED: Gent_MP_UniRef50_allSpecies_Gent02c
IMPORTED: Gent_MP_UniRef50_allSpecies_Gent03a
IMPORTED: Gent_MP_UniRef50_allSpecies_Gent03b
IMPORTED: Gent_MP_UniRef50_allSpecies_Gent03c
IMPORTED: Gent_MP_UniRef50_allSpecies_Gent04a
IMPORTED: Gent_MP_UniRef50_allSpecies_Gent04b
IMPORTED: Gent_MP_UniRef50_allSpecies_Gent04c
IMPORTED: Gent_MP_UniRef50_allSpecies_Gent05a
IMPORTED: Gent_MP_UniRef50_allSpecies_Gent05b
IMPORTED: Gent_MP_UniRef50_allSpecies_Gent05c
IMPORTED: Gent_MP_UniRef50_allSpecies_Gent06a
IMPORTED: Gent_MP_UniRef50_allSpecies_Gent06b
IMPORTED: Gent_MP_UniRef50_allSpecies_Gent06c
IMPORTED: Gent_MP_UniRef50_allSpecies_Gent07a
IMPORTED: Gent_MP_UniRef50_allSpecies_Gent07b
IMPORTED: Gent_MP_UniRef50_allSpecies_Gent07c
IMPORTED: Gent_MP_UniRef50_allSpecies_Gent08a
IMPORTED: Gent_MP_UniRef50_allSpecies_Gent08b
IMPORTED: Gent_MP_UniRef50_allSpecies_Gent08c
IMPORTED: Gent_MP_UniRef50_allSpecies_Gent09a
IMPORTED: Gent_MP_UniRef50_allSpecies_Gent09b
IMPORTED: Gent_MP_UniRef50_allSpecies_Gent09c
IMPORTED: Gent_MP_UniRef50_allSpecies_Gent10a
IMPORTED: Gent_MP_UniRef50_allSpecies_Gent10b
IMPORTED: Gent_MP_UniRef50_allSpecies_Gent10c
IMPORTED: Gent_MP_UniRef50_allSpecies_Gent11a
IMPORTED: Gent_MP_UniRef50_allSpecies_Gent11b
IMPORTED: Gent_MP_UniRef50_allSpecies_Gent11c
IMPORTED: Gent_MP_UniRef50_allSpecies_Gent12a
IMPORTED: Gent_MP_UniRef50_allSpecies_Gent12b
IMPORTED: Gent_MP_UniRef50_allSpecies_Gent12c
IMPORTED: Gent_MP_UniRef50_allSpecies_Gent13a
IMPORTED: Gent_MP_UniRef50_allSpecies_Gent13b
IMPORTED: Gent_MP_UniRef50_allSpecies_Gent13c
IMPORTED: Gent_MP_UniRef50_allSpecies_Gent14a
IMPORTED: Gent_MP_UniRef50_allSpecies_Gent14b
IMPORTED: Gent_MP_UniRef50_allSpecies_Gent14c
IMPORTED: Gent_MP_UniRef50_allSpecies_Gent15a
IMPORTED: Gent_MP_UniRef50_allSpecies_Gent15b
IMPORTED: Gent_MP_UniRef50_allSpecies_Gent15c
IMPORTED: Gent_MP_UniRef50_allSpecies_Gent16a
IMPORTED: Gent_MP_UniRef50_allSpecies_Gent16b
IMPORTED: Gent_MP_UniRef50_allSpecies_Gent16c
IMPORTED: Gent_MP_UniRef50_allSpecies_Gent17a
IMPORTED: Gent_MP_UniRef50_allSpecies_Gent17b
IMPORTED: Gent_MP_UniRef50_allSpecies_Gent17c
IMPORTED: Gent_MP_UniRef50_allSpecies_Gent18a
IMPORTED: Gent_MP_UniRef50_allSpecies_Gent18b
IMPORTED: Gent_MP_UniRef50_allSpecies_Gent18c
IMPORTED: Gent_MP_UniRef50_allSpecies_Gent19a
IMPORTED: Gent_MP_UniRef50_allSpecies_Gent19b
IMPORTED: Gent_MP_UniRef50_allSpecies_Gent19c
IMPORTED: Gent_MP_UniRef50_allSpecies_Gent20a
IMPORTED: Gent_MP_UniRef50_allSpecies_Gent20b
IMPORTED: Gent_MP_UniRef50_allSpecies_Gent20c
IMPORTED: Gent_MP_UniRef50_allSpecies_Gent21a
IMPORTED: Gent_MP_UniRef50_allSpecies_Gent21c
IMPORTED: Gent_MP_UniRef50_allSpecies_Gent21d
IMPORTED: Gent_MP_UniRef50_allSpecies_Gent22a
IMPORTED: Gent_MP_UniRef50_allSpecies_Gent22b
```

```
IMPORTED: Gent_MP_UniRef50_allSpecies_Gent21d
IMPORTED: Gent_MP_UniRef50_allSpecies_Gent22a
IMPORTED: Gent_MP_UniRef50_allSpecies_Gent22b
IMPORTED: Gent_MP_UniRef50_allSpecies_Gent22c
IMPORTED: Gent_MP_UniRef50_allSpecies_Gent23a
IMPORTED: Gent_MP_UniRef50_allSpecies_Gent23b
IMPORTED: Gent_MP_UniRef50_allSpecies_Gent23c
IMPORTED: Gent_MP_UniRef50_allSpecies_Gent24a
IMPORTED: Gent_MP_UniRef50_allSpecies_Gent24b
IMPORTED: Gent_MP_UniRef50_allSpecies_Gent24c
IMPORTED: Gent_MP_UniRef50_allSpecies_Gent25a
IMPORTED: Gent_MP_UniRef50_allSpecies_Gent25b
IMPORTED: Gent_MP_UniRef50_allSpecies_Gent25c
IMPORTED: Gent_MP_UniRef50_allSpecies_Gent26a
IMPORTED: Gent_MP_UniRef50_allSpecies_Gent26b
IMPORTED: Gent_MP_UniRef50_allSpecies_Gent26c
IMPORTED: Gent_MP_UniRef50_allSpecies_Gent27a
IMPORTED: Gent_MP_UniRef50_allSpecies_Gent27b
IMPORTED: Gent_MP_UniRef50_allSpecies_Gent27c
IMPORTED: Gent_MP_UniRef50_allSpecies_Gent28a
IMPORTED: Gent_MP_UniRef50_allSpecies_Gent28b
IMPORTED: Gent_MP_UniRef50_allSpecies_Gent28c
IMPORTED: Gent_MP_UniRef50_allSpecies_Gent29a
IMPORTED: Gent_MP_UniRef50_allSpecies_Gent29b
IMPORTED: Gent_MP_UniRef50_allSpecies_Gent29c
IMPORTED: Gent_MP_UniRef50_allSpecies_Gent30a
IMPORTED: Gent_MP_UniRef50_allSpecies_Gent30b
IMPORTED: Gent_MP_UniRef50_allSpecies_Gent30c
IMPORTED: Gent_MP_UniRef50_allSpecies_Gent31a
IMPORTED: Gent_MP_UniRef50_allSpecies_Gent31b
IMPORTED: Gent_MP_UniRef50_allSpecies_Gent31c
IMPORTED: Gent_MP_UniRef50_allSpecies_Gent32a
IMPORTED: Gent_MP_UniRef50_allSpecies_Gent32b
IMPORTED: Gent_MP_UniRef50_allSpecies_Gent32c
IMPORTED: Gent_MP_UniRef50_allSpecies_Gent33a
IMPORTED: Gent_MP_UniRef50_allSpecies_Gent33b
IMPORTED: Gent_MP_UniRef50_allSpecies_Gent33c
IMPORTED: Gent_MP_UniRef50_allSpecies_Gent34a
IMPORTED: Gent_MP_UniRef50_allSpecies_Gent34b
IMPORTED: Gent_MP_UniRef50_allSpecies_Gent34c
IMPORTED: Gent_MP_UniRef50_allSpecies_Gent35a
IMPORTED: Gent_MP_UniRef50_allSpecies_Gent35b
IMPORTED: Gent_MP_UniRef50_allSpecies_Gent35c
IMPORTED: Gent_MP_UniRef50_allSpecies_Gent36a
IMPORTED: Gent_MP_UniRef50_allSpecies_Gent36b
IMPORTED: Gent_MP_UniRef50_allSpecies_Gent36c
IMPORTED: Gent_MP_UniRef50_allSpecies_Gent37a
IMPORTED: Gent_MP_UniRef50_allSpecies_Gent37b
IMPORTED: Gent_MP_UniRef50_allSpecies_Gent37c
IMPORTED: Gent_MP_UniRef50_allSpecies_Gent38a
IMPORTED: Gent_MP_UniRef50_allSpecies_Gent38b
IMPORTED: Gent_MP_UniRef50_allSpecies_Gent38c
IMPORTED: Gent_MP_UniRef50_allSpecies_Gent39a
IMPORTED: Gent_MP_UniRef50_allSpecies_Gent39b
IMPORTED: Gent_MP_UniRef50_allSpecies_Gent39c
IMPORTED: Gent_MP_UniRef50_allSpecies_Gent40a
IMPORTED: Gent_MP_UniRef50_allSpecies_Gent40b
IMPORTED: Gent_MP_UniRef50_allSpecies_Gent40c
READ: Gent_MP_UniRef50_allSpecies_Gent01a
READ: Gent_MP_UniRef50_allSpecies_Gent01b
READ: Gent_MP_UniRef50_allSpecies_Gent01c
READ: Gent_MP_UniRef50_allSpecies_Gent02a
READ: Gent_MP_UniRef50_allSpecies_Gent02b
READ: Gent_MP_UniRef50_allSpecies_Gent02c
```

```
READ: Gent_MP_UniRef50_allSpecies_Gent02a
READ: Gent_MP_UniRef50_allSpecies_Gent02b
READ: Gent_MP_UniRef50_allSpecies_Gent02c
READ: Gent_MP_UniRef50_allSpecies_Gent03a
READ: Gent_MP_UniRef50_allSpecies_Gent03b
READ: Gent_MP_UniRef50_allSpecies_Gent03c
READ: Gent_MP_UniRef50_allSpecies_Gent04a
READ: Gent_MP_UniRef50_allSpecies_Gent04b
READ: Gent_MP_UniRef50_allSpecies_Gent04c
READ: Gent_MP_UniRef50_allSpecies_Gent05a
READ: Gent_MP_UniRef50_allSpecies_Gent05b
READ: Gent_MP_UniRef50_allSpecies_Gent05c
READ: Gent_MP_UniRef50_allSpecies_Gent06a
READ: Gent_MP_UniRef50_allSpecies_Gent06b
READ: Gent_MP_UniRef50_allSpecies_Gent06c
READ: Gent_MP_UniRef50_allSpecies_Gent07a
READ: Gent_MP_UniRef50_allSpecies_Gent07b
READ: Gent_MP_UniRef50_allSpecies_Gent07c
READ: Gent_MP_UniRef50_allSpecies_Gent08a
READ: Gent_MP_UniRef50_allSpecies_Gent08b
READ: Gent_MP_UniRef50_allSpecies_Gent08c
READ: Gent_MP_UniRef50_allSpecies_Gent09a
READ: Gent_MP_UniRef50_allSpecies_Gent09b
READ: Gent_MP_UniRef50_allSpecies_Gent09c
READ: Gent_MP_UniRef50_allSpecies_Gent10a
READ: Gent_MP_UniRef50_allSpecies_Gent10b
READ: Gent_MP_UniRef50_allSpecies_Gent10c
READ: Gent_MP_UniRef50_allSpecies_Gent11a
READ: Gent_MP_UniRef50_allSpecies_Gent11b
READ: Gent_MP_UniRef50_allSpecies_Gent11c
READ: Gent_MP_UniRef50_allSpecies_Gent12a
READ: Gent_MP_UniRef50_allSpecies_Gent12b
READ: Gent_MP_UniRef50_allSpecies_Gent12c
READ: Gent_MP_UniRef50_allSpecies_Gent13a
READ: Gent_MP_UniRef50_allSpecies_Gent13b
READ: Gent_MP_UniRef50_allSpecies_Gent13c
READ: Gent_MP_UniRef50_allSpecies_Gent14a
READ: Gent_MP_UniRef50_allSpecies_Gent14b
READ: Gent_MP_UniRef50_allSpecies_Gent14c
READ: Gent_MP_UniRef50_allSpecies_Gent15a
READ: Gent_MP_UniRef50_allSpecies_Gent15b
READ: Gent_MP_UniRef50_allSpecies_Gent15c
READ: Gent_MP_UniRef50_allSpecies_Gent16a
READ: Gent_MP_UniRef50_allSpecies_Gent16b
READ: Gent_MP_UniRef50_allSpecies_Gent16c
READ: Gent_MP_UniRef50_allSpecies_Gent17a
READ: Gent_MP_UniRef50_allSpecies_Gent17b
READ: Gent_MP_UniRef50_allSpecies_Gent17c
READ: Gent_MP_UniRef50_allSpecies_Gent18a
READ: Gent_MP_UniRef50_allSpecies_Gent18b
READ: Gent_MP_UniRef50_allSpecies_Gent18c
READ: Gent_MP_UniRef50_allSpecies_Gent19a
READ: Gent_MP_UniRef50_allSpecies_Gent19b
READ: Gent_MP_UniRef50_allSpecies_Gent19c
READ: Gent_MP_UniRef50_allSpecies_Gent20a
READ: Gent_MP_UniRef50_allSpecies_Gent20b
READ: Gent_MP_UniRef50_allSpecies_Gent20c
READ: Gent_MP_UniRef50_allSpecies_Gent21a
READ: Gent_MP_UniRef50_allSpecies_Gent21c
READ: Gent_MP_UniRef50_allSpecies_Gent21d
READ: Gent_MP_UniRef50_allSpecies_Gent22a
READ: Gent_MP_UniRef50_allSpecies_Gent22b
READ: Gent_MP_UniRef50_allSpecies_Gent22c
READ: Gent_MP_UniRef50_allSpecies_Gent23a
```

```
READ: Gent_MP_UniRef50_allSpecies_Gent22b
READ: Gent_MP_UniRef50_allSpecies_Gent22c
READ: Gent_MP_UniRef50_allSpecies_Gent23a
READ: Gent_MP_UniRef50_allSpecies_Gent23b
READ: Gent_MP_UniRef50_allSpecies_Gent23c
READ: Gent_MP_UniRef50_allSpecies_Gent24a
READ: Gent_MP_UniRef50_allSpecies_Gent24b
READ: Gent_MP_UniRef50_allSpecies_Gent24c
READ: Gent_MP_UniRef50_allSpecies_Gent25a
READ: Gent_MP_UniRef50_allSpecies_Gent25b
READ: Gent_MP_UniRef50_allSpecies_Gent25c
READ: Gent_MP_UniRef50_allSpecies_Gent26a
READ: Gent_MP_UniRef50_allSpecies_Gent26b
READ: Gent_MP_UniRef50_allSpecies_Gent26c
READ: Gent_MP_UniRef50_allSpecies_Gent27a
READ: Gent_MP_UniRef50_allSpecies_Gent27b
READ: Gent_MP_UniRef50_allSpecies_Gent27c
READ: Gent_MP_UniRef50_allSpecies_Gent28a
READ: Gent_MP_UniRef50_allSpecies_Gent28b
READ: Gent_MP_UniRef50_allSpecies_Gent28c
READ: Gent_MP_UniRef50_allSpecies_Gent29a
READ: Gent_MP_UniRef50_allSpecies_Gent29b
READ: Gent_MP_UniRef50_allSpecies_Gent29c
READ: Gent_MP_UniRef50_allSpecies_Gent30a
READ: Gent_MP_UniRef50_allSpecies_Gent30b
READ: Gent_MP_UniRef50_allSpecies_Gent30c
READ: Gent_MP_UniRef50_allSpecies_Gent31a
READ: Gent_MP_UniRef50_allSpecies_Gent31b
READ: Gent_MP_UniRef50_allSpecies_Gent31c
READ: Gent_MP_UniRef50_allSpecies_Gent32a
READ: Gent_MP_UniRef50_allSpecies_Gent32b
READ: Gent_MP_UniRef50_allSpecies_Gent32c
READ: Gent_MP_UniRef50_allSpecies_Gent33a
READ: Gent_MP_UniRef50_allSpecies_Gent33b
READ: Gent_MP_UniRef50_allSpecies_Gent33c
READ: Gent_MP_UniRef50_allSpecies_Gent34a
READ: Gent_MP_UniRef50_allSpecies_Gent34b
READ: Gent_MP_UniRef50_allSpecies_Gent34c
READ: Gent_MP_UniRef50_allSpecies_Gent35a
READ: Gent_MP_UniRef50_allSpecies_Gent35b
READ: Gent_MP_UniRef50_allSpecies_Gent35c
READ: Gent_MP_UniRef50_allSpecies_Gent36a
READ: Gent_MP_UniRef50_allSpecies_Gent36b
READ: Gent_MP_UniRef50_allSpecies_Gent36c
READ: Gent_MP_UniRef50_allSpecies_Gent37a
READ: Gent_MP_UniRef50_allSpecies_Gent37b
READ: Gent_MP_UniRef50_allSpecies_Gent37c
READ: Gent_MP_UniRef50_allSpecies_Gent38a
READ: Gent_MP_UniRef50_allSpecies_Gent38b
READ: Gent_MP_UniRef50_allSpecies_Gent38c
READ: Gent_MP_UniRef50_allSpecies_Gent39a
READ: Gent_MP_UniRef50_allSpecies_Gent39b
READ: Gent_MP_UniRef50_allSpecies_Gent39c
READ: Gent_MP_UniRef50_allSpecies_Gent40a
READ: Gent_MP_UniRef50_allSpecies_Gent40b
READ: Gent_MP_UniRef50_allSpecies_Gent40c
MetaProtein files successfully read:
Matrix was normalized and filtered by threshold 0.01 successfully
CrossCorrelation with Spearman matrix successfully calculated
```

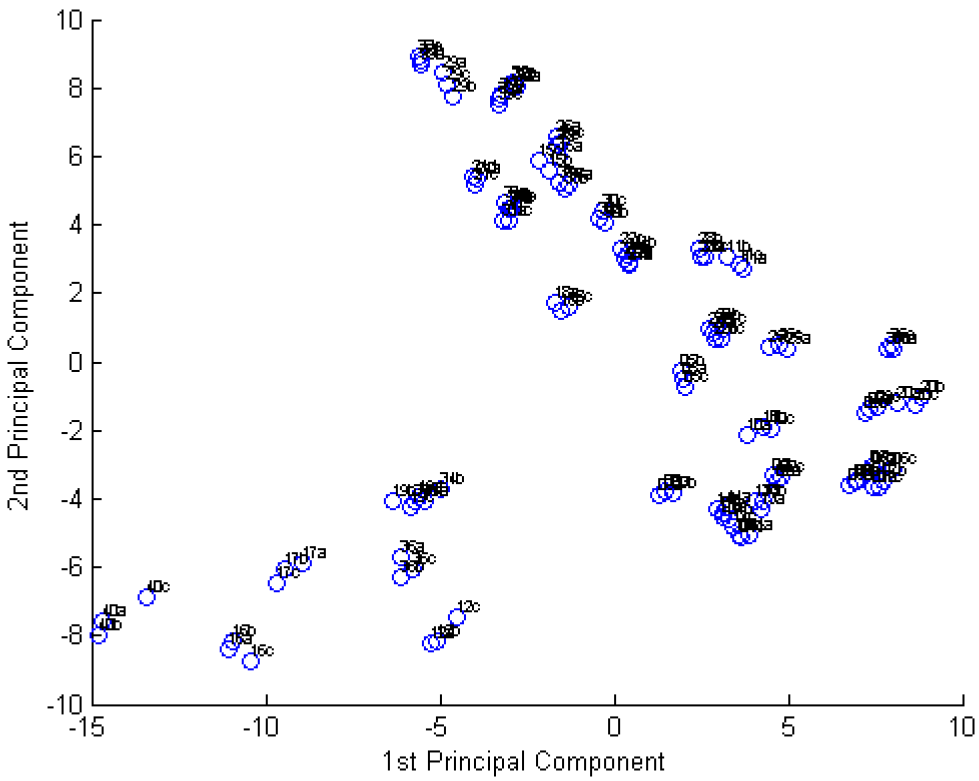
2nd Principal Component
1st Principal Component
10
8
6
4
2
0
-2
-4
-6
-8
-10
-15
-10
-5
0
5
10

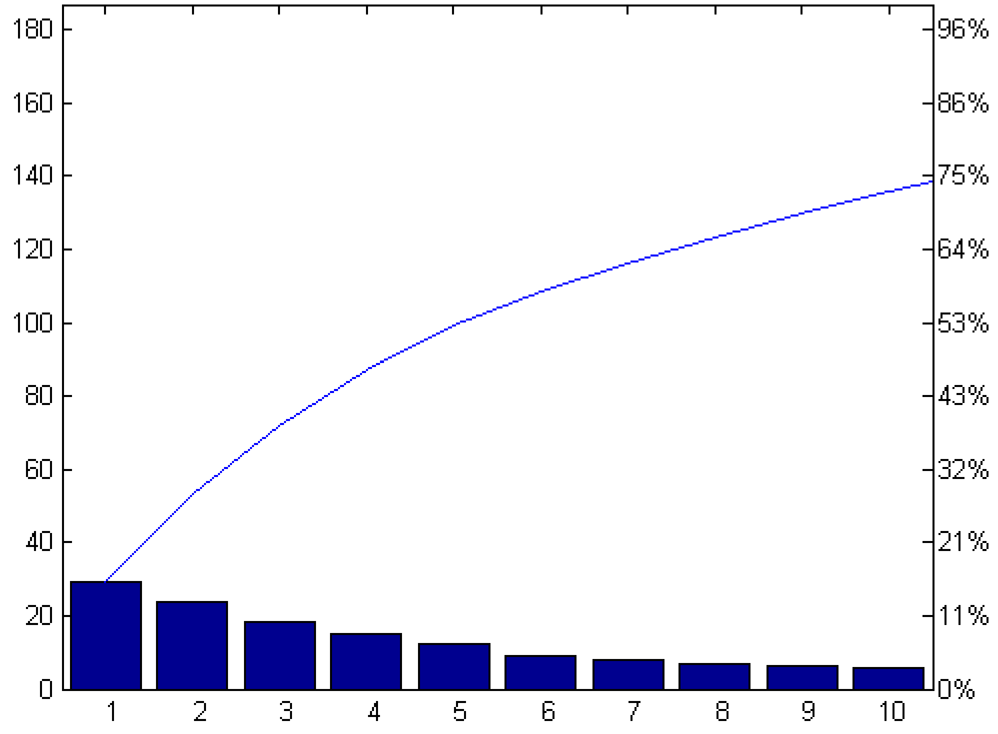
180
160
140
120
100
80
60
40
20
0
96%
86%
75%
64%
53%
43%
32%
21%
11%
0%
1
2
3
4
5
6
7
8
9
10

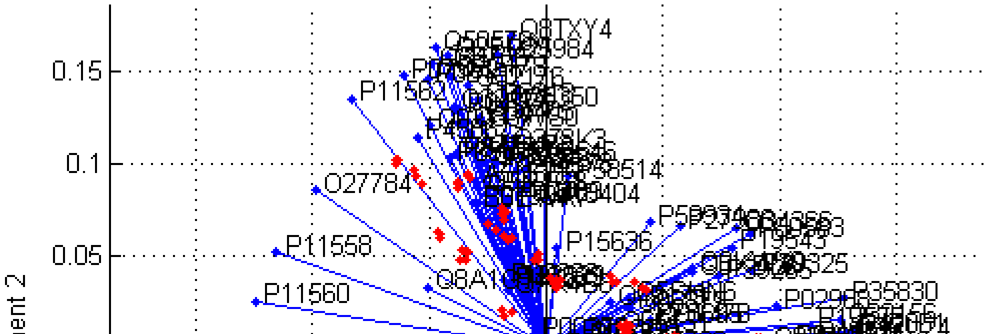
0.15
0.1
0.05
ent 2
Q8TXY4
P11562
O27784
P11558
P11560
P15636
P35830

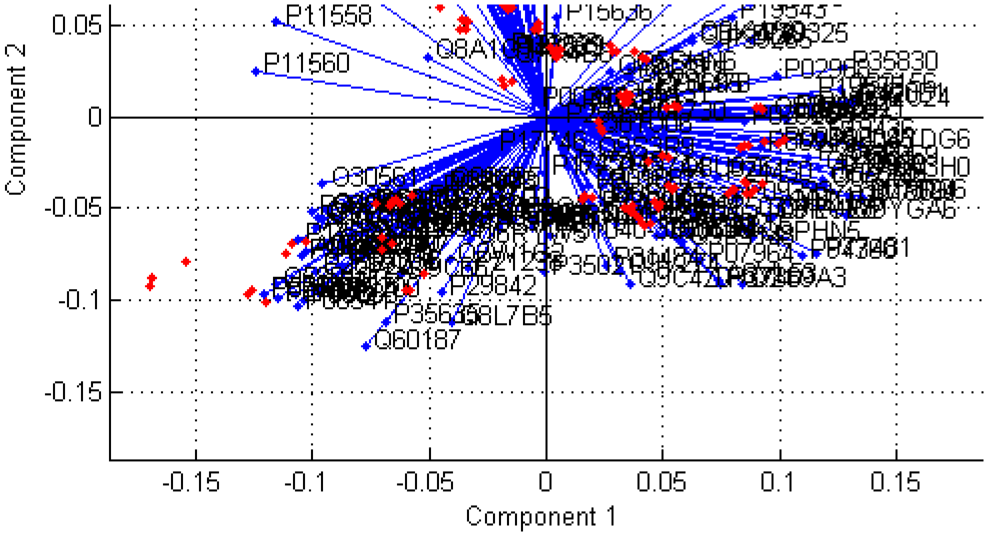
Component 2
Component 1
0.05
0
-0.05
-0.1
-0.15
-0.15
-0.1
-0.05
0
0.05
0.1
0.15
P11558
P11560
P35830
Q60187
P29842

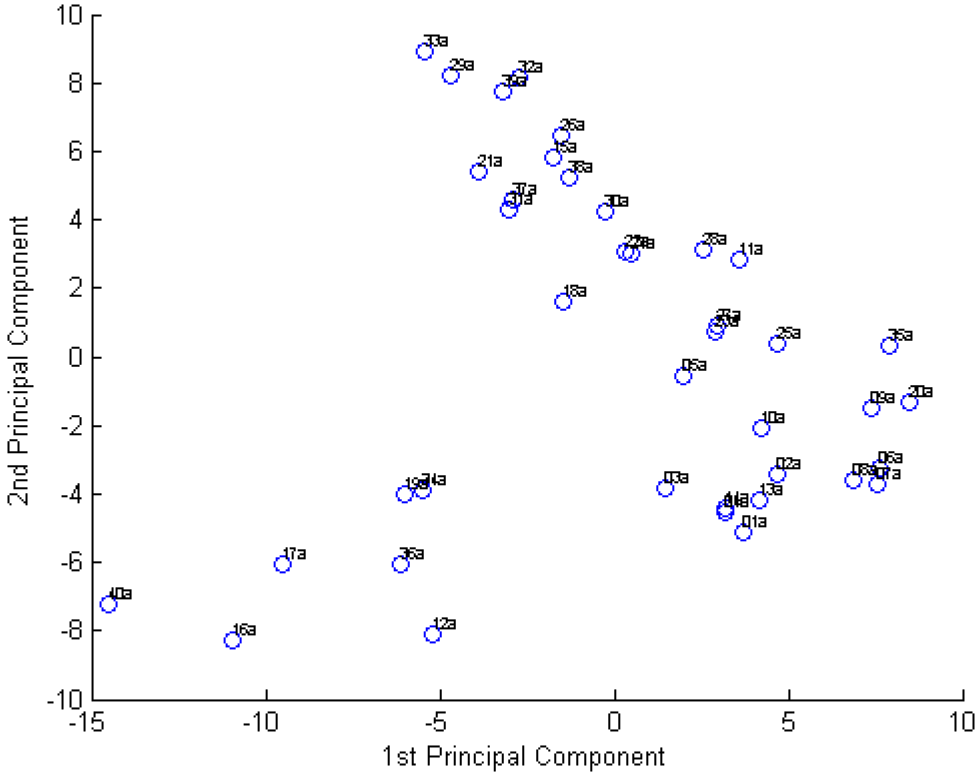
2nd Principal Component
1st Principal Component
10
8
6
4
2
0
-2
-4
-6
-8
-10
-15
-10
-5
0
5
10

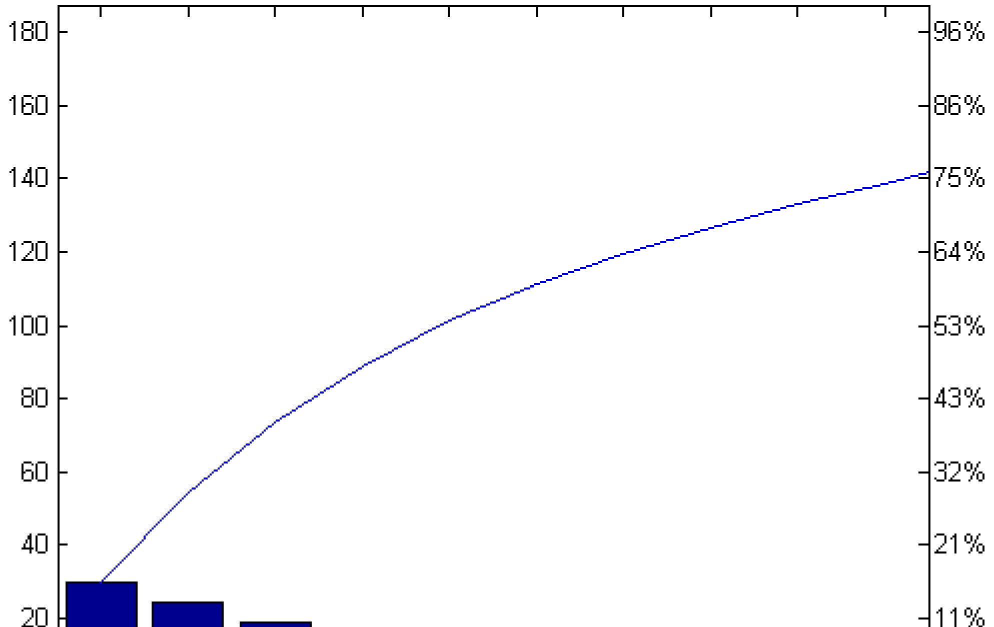
180
160
140
120
100
80
60
40
20
96%
86%
75%
64%
53%
43%
32%
21%
11%

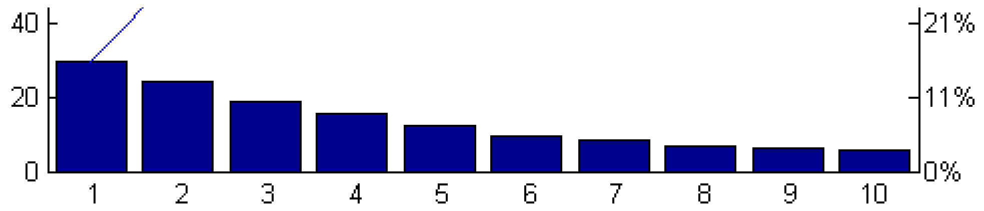

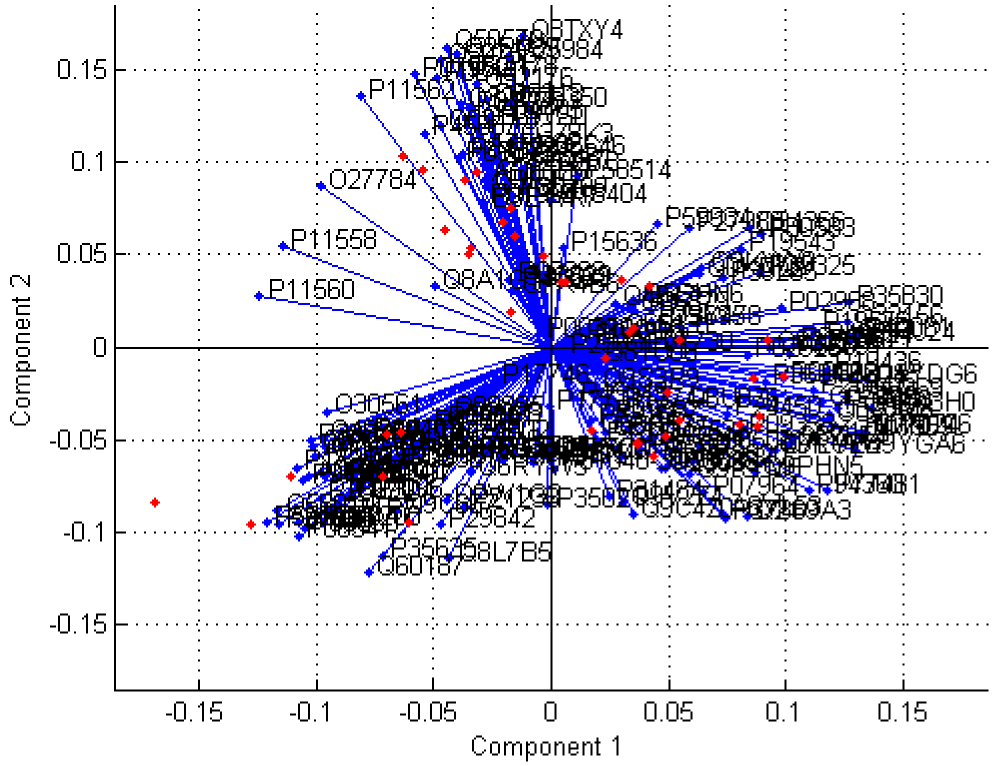

- Sto... Proteobacteria – Wiki... Fachagentur Nachwac... Basisdaten Bioen
File Tools Desktop Window Help

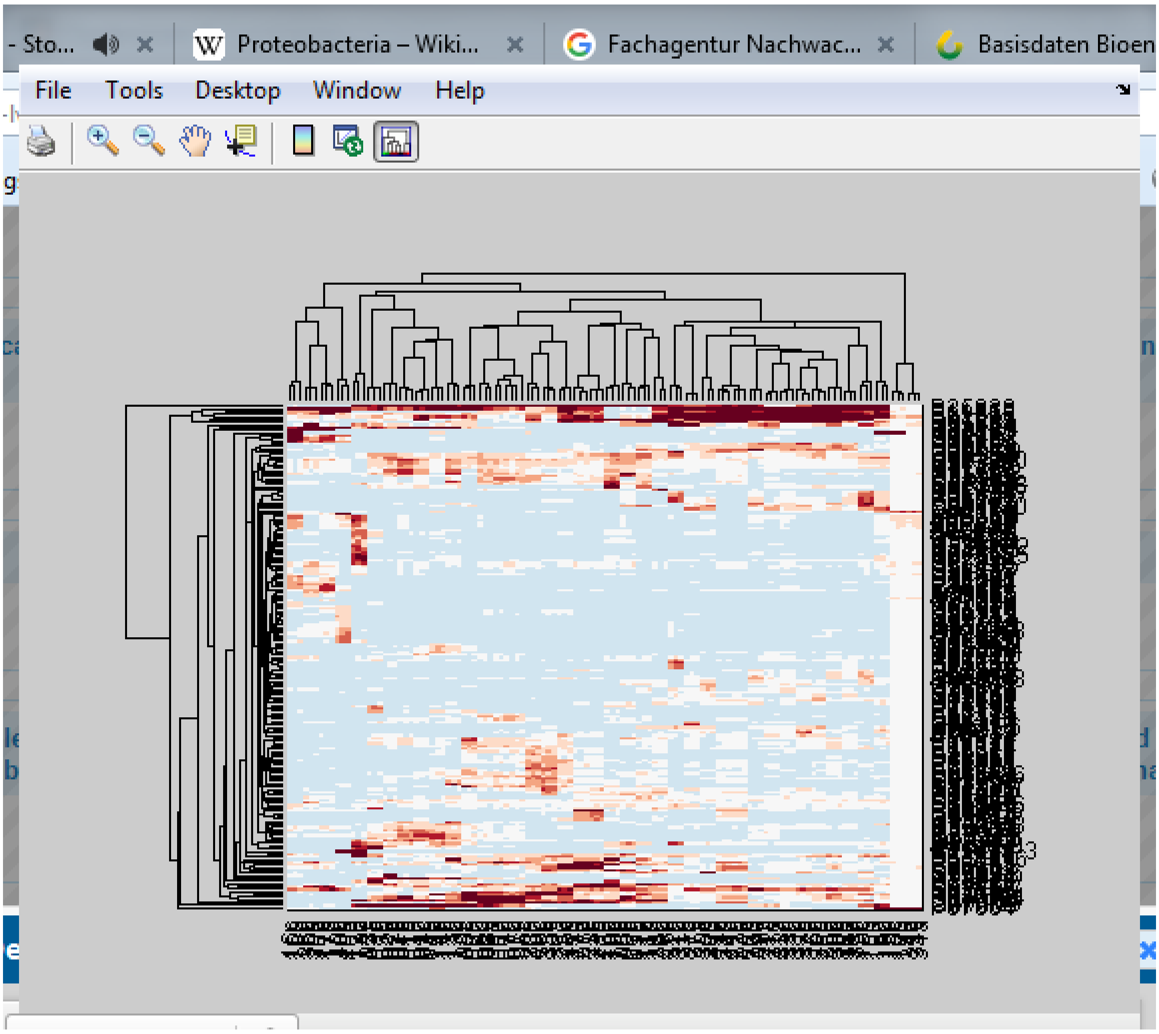

Perform mixed correlations necessary that both matrixes has the same number of entries

Metadata

```
[meta_taxo_correlationMatrix ] = CalculateCorrelation_V2(meta_matrix, tax_average_matrix_filt
[meta_onto_correlationMatrix ] = CalculateCorrelation_V2(meta_matrix, onto_average_matrix_fil
[meta_mp_correlationMatrix ] = CalculateCorrelation_V2(meta_matrix, mp_average_matrix_filtere
% Taxodata
[taxo_meta_correlationMatrix ] = CalculateCorrelation_V2(tax_average_matrix_filtered, meta_ma
[taxo_onto_correlationMatrix ] = CalculateCorrelation_V2(tax_average_matrix_filtered, onto_av
[taxo_mp_correlationMatrix ] = CalculateCorrelation_V2(tax_average_matrix_filtered, mp_averag

% Ontodata
[onto_meta_correlationMatrix] = CalculateCorrelation_V2(onto_average_matrix_filtered,meta_mat
[onto_taxo_correlationMatrix ] = CalculateCorrelation_V2(onto_average_matrix_filtered,tax_ave
[onto_mp_correlationMatrix ] = CalculateCorrelation_V2(onto_average_matrix_filtered, mp_avera

% MPdata
[mp_meta_correlationMatrix ] = CalculateCorrelation_V2(mp_average_matrix_filtered,meta_matrix
[mp_taxo_correlationMatrix ] = CalculateCorrelation_V2(mp_average_matrix_filtered,tax_average
[mp_onto_correlationMatrix ] = CalculateCorrelation_V2(mp_average_matrix_filtered, onto_avera
```

```
CrossCorrelation with Spearman matrix successfully calculated
CrossCorrelation with Spearman matrix successfully calculated
CrossCorrelation with Spearman matrix successfully calculated
CrossCorrelation with Spearman matrix successfully calculated
CrossCorrelation with Spearman matrix successfully calculated
CrossCorrelation with Spearman matrix successfully calculated
CrossCorrelation with Spearman matrix successfully calculated
CrossCorrelation with Spearman matrix successfully calculated
```

```
CrossCorrelation with Spearman matrix successfully calculated
CrossCorrelation with Spearman matrix successfully calculated
CrossCorrelation with Spearman matrix successfully calculated
CrossCorrelation with Spearman matrix successfully calculated
CrossCorrelation with Spearman matrix successfully calculated
CrossCorrelation with Spearman matrix successfully calculated
CrossCorrelation with Spearman matrix successfully calculated
```

FINISH

```
disp('Finish')
```

```
Finish
```

Published with MATLAB® R2014a
